# Regulating the Assembly of γ‑Cyclodextrin Host and Polyoxometalate-Based Guests toward Light-Responsive Hybrid Rotaxanes

**DOI:** 10.1021/jacs.5c06495

**Published:** 2025-07-29

**Authors:** Wu-Ji Chen, Chun-Yan Liu, Yun-Jing Mu, Yi-An Yin, Chang-Gen Lin, De-Liang Long, Leroy Cronin, Yu-Fei Song

**Affiliations:** † State Key Laboratory of Chemical Resource Engineering, 47832 Beijing University of Chemical Technology, Beijing 100029, P. R. China; ‡ School of Chemistry, 3526 The University of Glasgow, Glasgow G11 6EW, U.K.

## Abstract

The rational design of hybrid organic–inorganic rotaxanes is crucial for advancing molecular machines and functional nanomaterials, yet the integration of metal-oxo clusters into interlocked systems remains challenging. Herein, we present a precision synthesis strategy for hybrid rotaxanes combining γ-cyclodextrin (γ-CD) hosts with Anderson-type polyoxometalate (POM)-based guests. This approach utilizes covalent POM modification coupled with strategically anchored organic functionalities to control supramolecular assembly, enabling the construction of *pseudo*-[2]-, [3]-, and [4]­rotaxanes with controlled structural variations. Significantly, we achieved the first single-crystal examples of supramolecular *pseudo*-[4]­rotaxanes featuring γ-CD dimers threaded by two organo-POM units. A key breakthrough was achieved through light-induced single-crystal-to-single-crystal transformation of these *pseudo*-[4]­rotaxanes, producing hybrid [3]­rotaxanes containing uniquely arranged *anti*-head-to-tail anthracene dimers—the first reported photoresponsive architecture of this type. These structural transformations demonstrate the dynamic, stimuli-responsive character of these hybrid systems. This work establishes a new paradigm for the precision engineering of rotaxanes using organo-POM building blocks, revealing their remarkable potential for creating smart materials with programmable structural changes. The successful integration of covalent modification, supramolecular templating, and photoresponsive components provides a powerful platform for developing next-generation multifunctional molecular machines and adaptive nanomaterials with precisely controlled properties.

## Graphical abstract

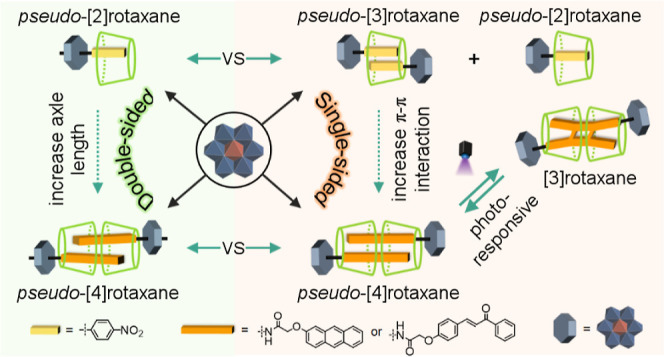

## Introduction

Rotaxanes, a key subclass of mechanically interlocked molecules (MIMs),^1^ consist of macrocyclic “wheels” threaded onto dumbbell-shaped “axles” capped with sterically bulky “stoppers” that prevent dethreading.^2^ The [*n*]­rotaxane nomenclature indicates a system of *n* interlocked components, while *pseudo*-rotaxanes represent their dynamic, stopper-lacking supramolecular analogues. These architectures have found widespread applications in catalysis,^3^ molecular machines,^4^ room-temperature phosphorescence,^5^ and smart materials^6^ due to their unique interlocked structures and stimuli-responsive behavior. A landmark achievement in this field is the γ-cyclodextrin (γ-CD)-templated enantioselective photodimerization of anthracene derivatives, which achieved up to 32% enantiomeric excess (ee) for the *syn*-head-to-tail configuration (Figure 1a).^7^ Recent progress has focused on incorporating metal-coordination motifs as wheels to create hybrid rotaxanes, enabling novel functionalities in qubits, sensing, and nonlinear optical materials.^8–14^ Despite significant advances in the rational design of macrocyclic wheels and axles in MIMs, the functional role of stoppers remains poorly understood, particularly their impact on solution-phase dynamics and solid-state structural organizations.

**Figure 1:**
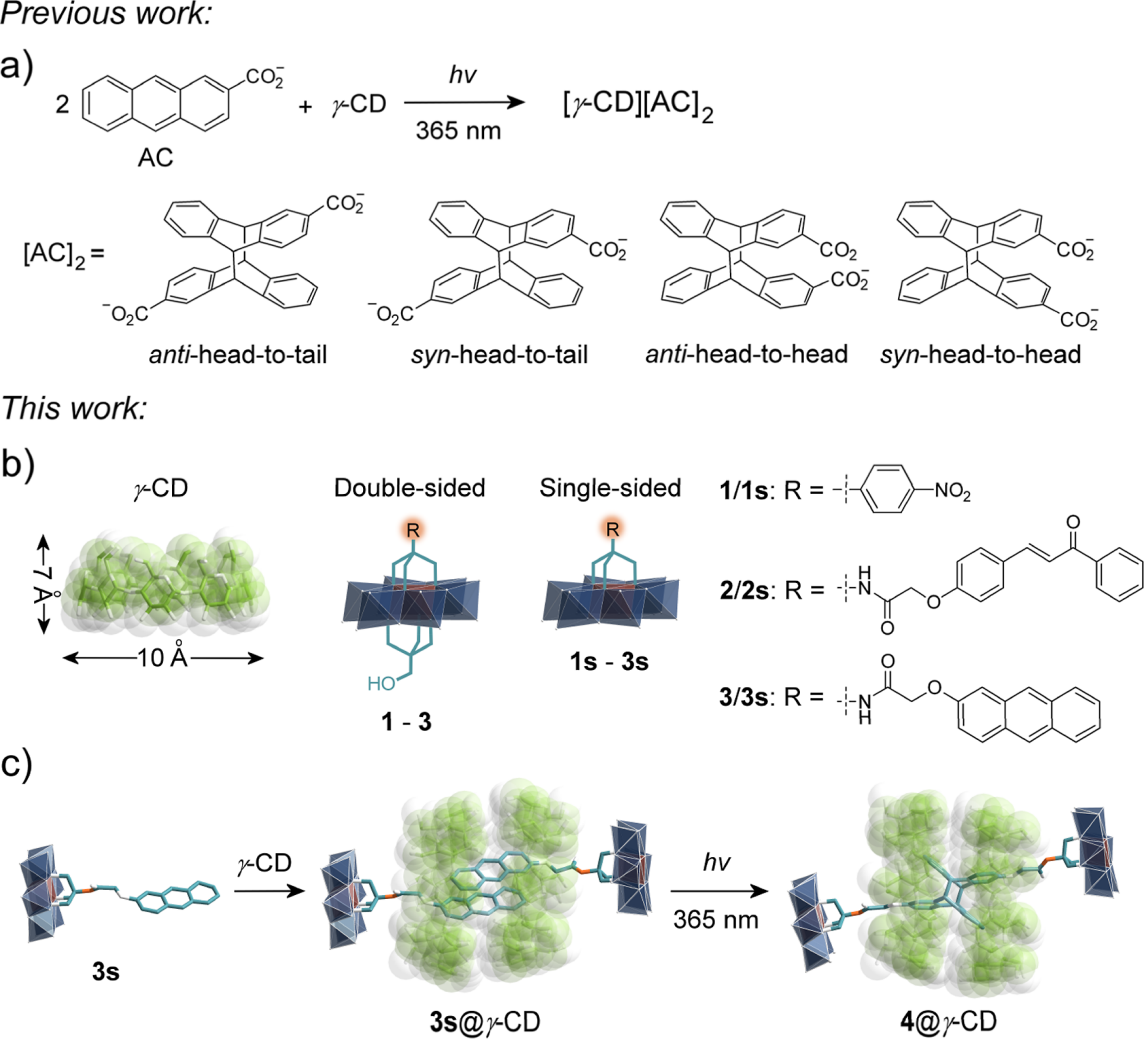
Schematic representation of (a) the photodimerization in MIMs formed by 2-anthracenecarboxylates and γ-CDs,^7^ (b) the structures of γ-CD and asymmetric Anderson-type POM {AlMo_6_O_24_} hybrids, and (c) the *pseudo*-[4]­rotaxane and photodimerized [3]­rotaxane formed by POM hybrid **3s** and γ-CD. Color code: {MoO_6_}, dark blue octahedron; {AlO_6_}, brown octahedron; C, green in γ-CD and teal in POM hybrids; N, orange; and O, white.

We therefore identified polyoxometalates (POMs)—discrete anionic metal-oxo clusters of early transition metals in their highest oxidation states^15–24^ —as ideal inorganic stoppers for hybrid (*pseudo*-)­rotaxane construction. POMs offer two key advantages: (i) their capacity for site-specific covalent functionalization enables rational design of hybrid architectures with programmable functionality, and (ii) their well-defined steric bulk provides effective stopper performance.^25–29^ Previous works have demonstrated that organo-POM hybrids can be incorporated into rotaxane systems by utilizing their organic moieties as threading axles while employing the inorganic clusters as steric barriers, with macrocyclic hosts like CDs serving as effective templates.^30–34^

The supramolecular recognition between organo-POMs and CDs occurs through hydrophobic guest–host interactions, fundamentally differing from the chaotropic interactions that drive unmodified POM-CD complexation (where POM’s low charge density disrupts water structure).^35–48^ This distinction led us to hypothesize that two key variables could synergistically control assembly thermodynamics and topology: (i) the covalent modification patterns of the POM cluster and (ii) the chemical nature of anchored organic units. By systematically tuning these parameters, we predicted precise control over POM stoppers’ steric and recognition properties.

We investigated how site-specific covalent modification of Al-Anderson-type POMs {AlMo_6_O_24_} ^49–51^ dictates their recognition with γ-CDs (Figure 1b). Through comparative studies of double-sided versus single-sided asymmetric functionalization, we elucidated the distinct roles of steric effects and hydrogen-bonding interactions (mediated by free μ_3_-O sites) in directing assembly pathways. Incorporating photoresponsive chalcone and anthracene derivatives enabled light-modulated supramolecular behavior. This strategy produced a series of well-defined POM-CD complexes, including *pseudo*-[2], [3], and [4]­rotaxanes, all characterized by single-crystal X-ray diffraction. Our most significant achievement was the photoinduced single-crystal-to-single-crystal transformation of a *pseudo*-[4]­rotaxane to a [3]­rotaxane via anthracene dimerization under mild irradiation (Figure 1c). Our work establishes two landmark advances: (i) the first *pseudo*-[4]­rotaxanes with γ-CD dimers threaded by two organo-POM axles; (ii) the first [3]­rotaxane system containing pure *anti*-head-to-tail anthracene dimers—a configuration inaccessible through conventional synthesis.

## Results and Discussion

In our previous work, we reported the assembly of symmetrically functionalized Anderson hybrids with γ-CD, which characterized a poly-*pseudo*-rotaxane structure with two nitrobenzene units partially encapsulated in γ-CD cavities.^34^ Here, using the double-sided asymmetric **1** (Figure 1b), we anticipated a *pseudo*-[3]­rotaxane with a 2:1 (**1**:γ-CD) stoichiometry. Surprisingly, crystallization—even with excess γ-CD—yielded instead a 1:1 *pseudo*-[2]­rotaxane, **1**@γ-CD, as unequivocally demonstrated by single-crystal X-ray diffraction (Figure 2a). The structure reveals close contacts between protons H_a_/H_b_ of **1** and γ-CD’s bridging oxygen atoms. The **1**@γ-CD assembly was further confirmed by ^1^H NMR, 2D nuclear Overhauser effect spectroscopy (NOESY), electrospray ionization time-of-flight mass spectroscopy (ESI-TOF-MS), and isothermal titration calorimetry (ITC) (Figures S11–S15). Notably, in the crystal lattice, potassium ions bridge **1**@γ-CD units into triangular oligomers (Figure 2b).

**Figure 2:**
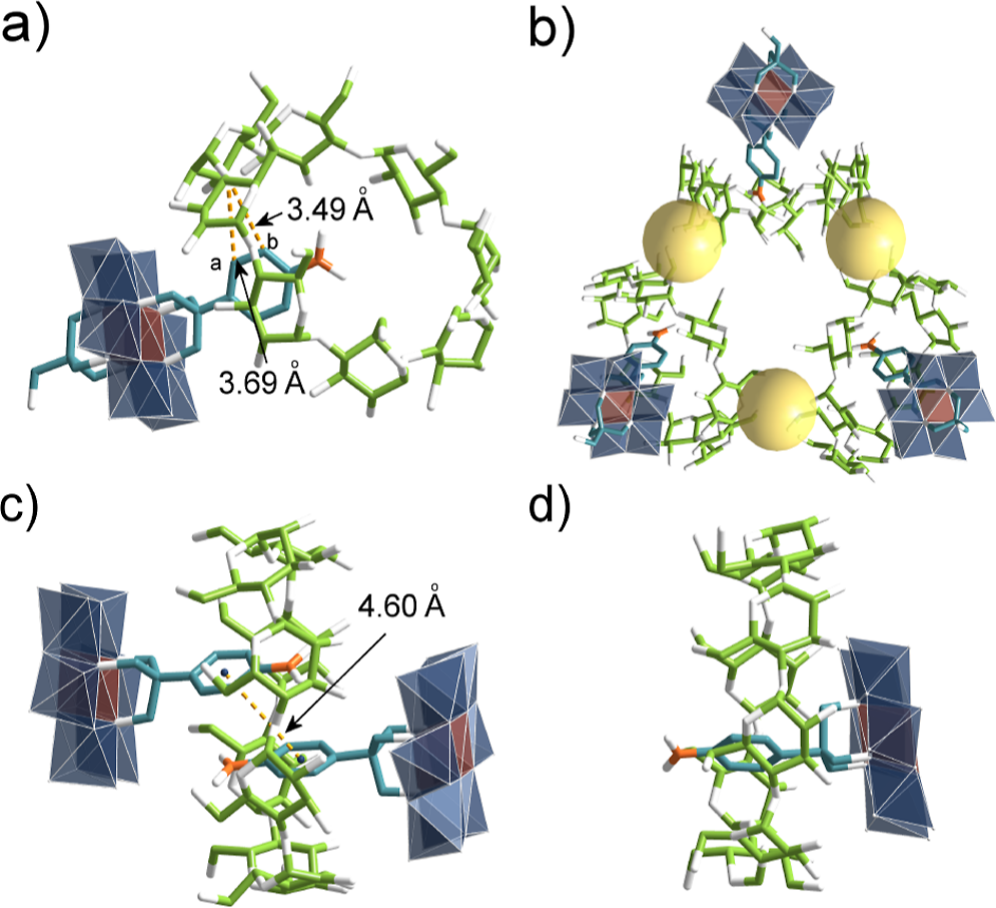
(a) The single-crystal structure of complex **1**@γ-CD, (b) the triangle oligomer of complex **1**@γ-CD linked by K^+^ ions, and (c) the *pseudo*-[3]­rotaxane structure and (d) the *pseudo*-[2]­rotaxane in complex **1s**@γ-CD. Color code: {MoO_6_}, dark blue octahedron; {AlO_6_}, brown octahedron; C, green in γ-CD and teal in **1**; N, orange; O, white; and K, yellow sphere.

Slow evaporation of an aqueous solution containing near-equimolar amounts of single-sided hybrid **1s** and γ-CD yielded X-ray quality crystals of **1s**@γ-CD. Unlike the **1**@γ-CD complex, the **1s**@γ-CD contains both *pseudo*-[3]­rotaxane and *pseudo*-[2]­rotaxane species (Figure 2c,d). In the *pseudo*-[3]­rotaxane, two nitrobenzene units are deeply inserted into the γ-CD cavity (separation: 4.60 Å), significantly closer than the 6.22 Å distance observed in our previously reported symmetric system.^34^ The *pseudo*-[2]­rotaxane shows complete nitrobenzene inclusion, evidenced by strong NOESY correlations between H_a_/H_b_ of **1s** and γ-CD’s H_5_/H_6_ protons (Figures S18, S19). The asymmetric unit contains two *pseudo*-[3]­rotaxanes, one *pseudo*-[2]­rotaxane, and one free γ-CD, yielding an overall 5:4 (**1s**:γ-CD) stoichiometry (Figure S20).

This distinct assembly behavior stems from **1s**′s accessible μ_3_-O sites, which form strong hydrogen bonding with γ-CD hydroxyl groups (Figure S20). In contrast, double-sided **1** lacks these interactions. The effect of hydrogen bonding has been confirmed by ^27^Al NMR, where **1s**@γ-CD exhibited a clear upfield shift (Δδ = −0.27 ppm) when compared with single-sided **1s** (Figure S22), while a negligible shift was observed in the case of **1**@γ-CD (Figure S21). ESI-TOF-MS confirmed both *pseudo*-[2] and [3]­rotaxane species in **1s**@γ-CD (Figure S23), while ITC measurements (*K* = 7.36 × 10^3^ M^–1^) verified the 5:4 stoichiometry of **1s**@γ-CD and revealed twice the binding affinity of **1**@γ-CD (Figure S24), consistent with preferential *pseudo*-[3]­rotaxane formation in **1s**@γ-CD (Table S1).

Encouraged by these results, we next examined γ-CD complexation with chalcone-functionalized hybrids **2** and **2s**, anticipating UV-triggered [2 + 2] cycloaddition on the π-conjugated chalcone groups.^52^ Herein, we focus on a discussion of the details for **2**. Contrary to our hypothesis of 2:1 *pseudo*-[3]­rotaxane formation, multiple lines of evidence indicated 1:1 complexation of **2** with γ-CD in solution: (i) ^1^H NMR showed significant upfield shifts of the chalcone protons upon γ-CD addition, particularly for H_d_ and H_c_ (Δδ = −0.66 and −0.44, respectively) of the α, β-unsaturated carbonyl group and H_i_ (Δδ = −0.57) (Figure 3a, S27), suggesting there may be a deep threading of the chalcone moiety in γ-CD; (ii) the Job plot analysis (UV–vis at 340 nm) showed maximum complexation at 1:1 ratio (Figure 3b); and (iii) ITC confirmed 1:1 stoichiometry (*K* = 1.38 × 10^3^ M^–1^) (Figure 3c and Table S1). While these results clearly demonstrate 1:1 complex formation, precise intermolecular interactions require further investigation.

**Figure 3:**
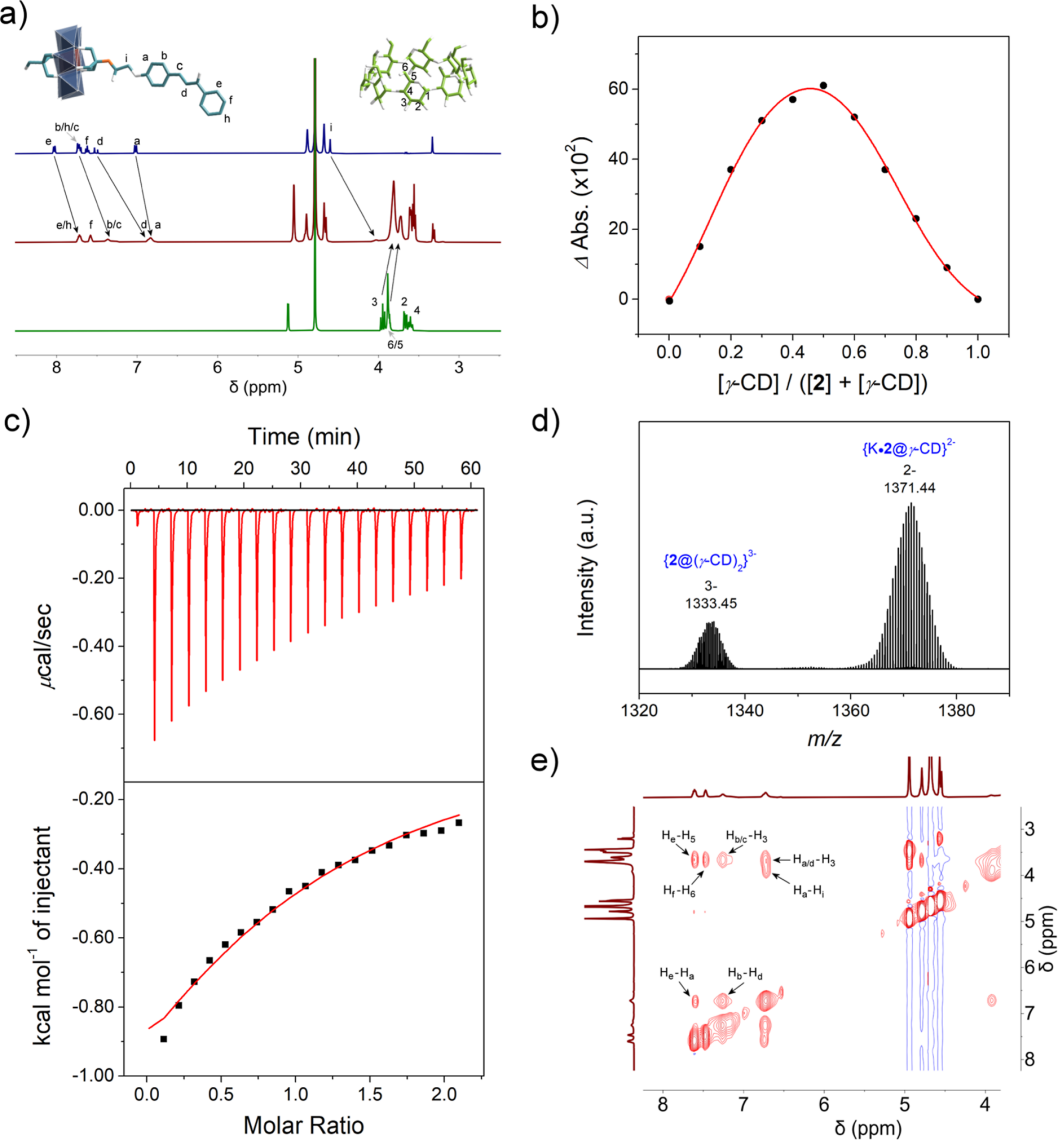
(a) The ^1^H NMR spectrum of **2** (top), **2**@γ-CD (middle), and γ-CD (bottom) in D_2_O; (b) the Job plot of the UV–vis absorbance changes at 340 nm showing the molar ratio of γ-CD and **2** upon complexation; (c) the ITC thermogram (top) and isotherm (bottom) of **2**@γ-CD, dots and lines correspond to experimental and theoretical heat values, respectively; (d) the ESI-TOF-MS of **2**@γ-CD at *m*/*z* = 1371.44 and 1333.45; and e) the 2D NOESY ^1^H NMR spectrum of **2**@γ-CD in D_2_O.

Through slow evaporation of an aqueous solution containing **2** and 10 equiv of γ-CD, we obtained single crystals of the **2**@γ-CD complex. X-ray analysis revealed that **2**@γ-CD crystallizes in an orthorhombic system with a *P*2_1_2_1_2_1_ space group, two asymmetric **2** and two γ-CDs, forming a *pseudo*-[4]­rotaxane structure of 2:2 adduct in the asymmetric unit (Figure 4a). Based on the conformations of the oxy-acetamide groups, *i*.*e*., the positions of the two oxygen atoms (highlighted in Figures 4a, S28), the two asymmetric **2** molecules can be classified into *Z*- and *E*-isomers. Notably, the chalcone moieties of the isomers thread into a “head-to-head” γ-CD dimer. While previous reports have described *pseudo*-[3]­rotaxanes with α-CD dimers^53,54^ or β-CD dimers,^55,56^ **2**@γ-CD represents the first case of *pseudo*-[4]­rotaxane structure featuring two guest molecules threaded through a γ-CD pair. The observed 1:1 molar ratio in complex **2**@γ-CD agrees perfectly with our titration experiments.

**Figure 4:**
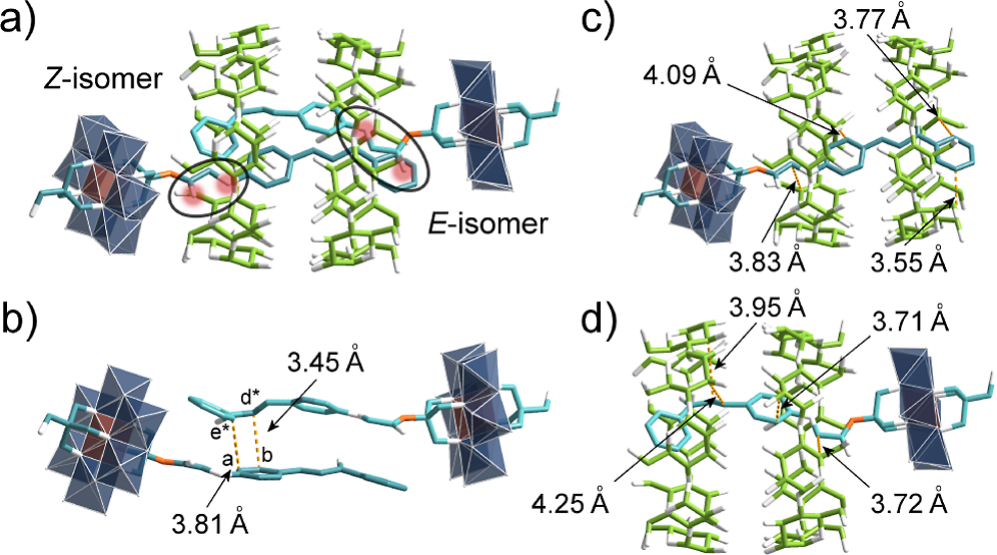
(a) The single-crystal structure of complex **2**@γ-CD (the *Z*- and *E*-isomers are labeled), (b) the intermolecular distances (in yellow dotted lines) between the chalcone molecules in complex **2**@γ-CD, and (c and d) the intermolecular distances between γ-CD and the *Z*- and *E*-isomers of **2**. Color code: the same as that in Figure 2, and carbon in *E*-isomer is represented in cyan.

ESI-TOF-MS analysis of **2**@γ-CD (Figures 3d, S29) revealed two major fragments: a 1:1 adduct of **2** and γ-CD, and a 1:2 adduct. The absence of a 2:2 adduct likely results from fragmentation during ionization. The presence of the 1:2 adduct, such as {**2**@(γ-CD)_2_}^3–^ species, suggests these complexes may exist transiently in solution, though the thermodynamically favored 2:2 adduct crystallizes in the solid state exclusively, as confirmed by crystallization experiments across various stoichiometric ratios (1:1 to 1:10).

The *Z*- and *E*-isomers in **2**@γ-CD exhibit close through-space contacts, with C_b_···C_d*_ and C_a_···C_e*_ distances measured at 3.45 and 3.81 Å, respectively (Figure 4b). These interactions were corroborated by 2D NOESY, showing spatial correlations between H_b_-H_d_ and H_a_-H_e_ (Figure 3e). Additional interactions between γ-CD and the two isomers were observed (Figure 4c,d), including close C_i_-C_5_ contacts (3.83 Å for *Z*-isomer and 3.72 Å for *E*-isomer) that explain the upfield shift of H_i_ in ^1^H NMR (Figure 3a). Despite these proximities, the α, β-unsaturated carbonyl groups remain separated by 4.46–5.36 Å—beyond the 4.2 Å Schmidt distance^57^ for [2 + 2] photocycloaddition (Figure S28). Parallel studies with single-sided **2s** yielded similar inclusion complexes (Figures S31–S35), though we were unable to obtain X-ray quality crystals of **2s**@γ-CD.

To further validate the formation of *pseudo*-[4]­rotaxanes between γ-CD and asymmetric Anderson hybrids, while also pursuing the targeted synthesis of [3]­rotaxanes through photoinduced single-crystal-to-single-crystal transformation, we focused on the asymmetric hybrid **3**, which contains a more π-conjugated anthracene moiety. The flat and π-conjugated anthracene was strategically incorporated to enhance the π–π interactions, thereby facilitating the reduction of intermolecular distances required to meet the Schmidt’s criteria for photodimerization. Using ^1^H NMR titration to monitor the complexation between **3** and γ-CD, we observed significantly more complex assembly behavior compared to the analogous system with hybrid **2**.

As shown in Figure 5, titration of γ-CD into a 6 mM solution of **3** induced characteristic upfield shifts of anthracene proton signals, accompanied by significant peak broadening indicative of rapid host–guest exchange dynamics. The complexation process has three distinct stages. The first stage is the initial binding (0–0.5 equiv of γ-CD), where progressive upfield shifts are observed for all anthracene protons except H_g_ and H_f_. This is consistent with the partial inclusion of the anthracene moiety within γ-CD cavities. The second stage is the structural reorganization (0.5–1 equiv of γ-CD), where chemical shift changes are plateaued and there is emergence of signal splitting for key protons: H_f_ bifurcated into downfield (f’) and upfield components, H_c_ developed new upfield resonance (c’), suggesting an equilibrium shift between multiple complexation modes. The third stage is the secondary complex formation (>1 equiv of γ-CD), where broad peaks (i’, d’) appear at chemical shifts matching free **3**, indicating the formation of new species in γ-CD-rich environment. This multistage evolution of NMR signatures reveals complex, concentration-dependent equilibria between distinct host–guest assemblies, with the observed splitting patterns being particularly suggestive of structural reorganization at intermediate γ-CD concentrations.

**Figure 5:**
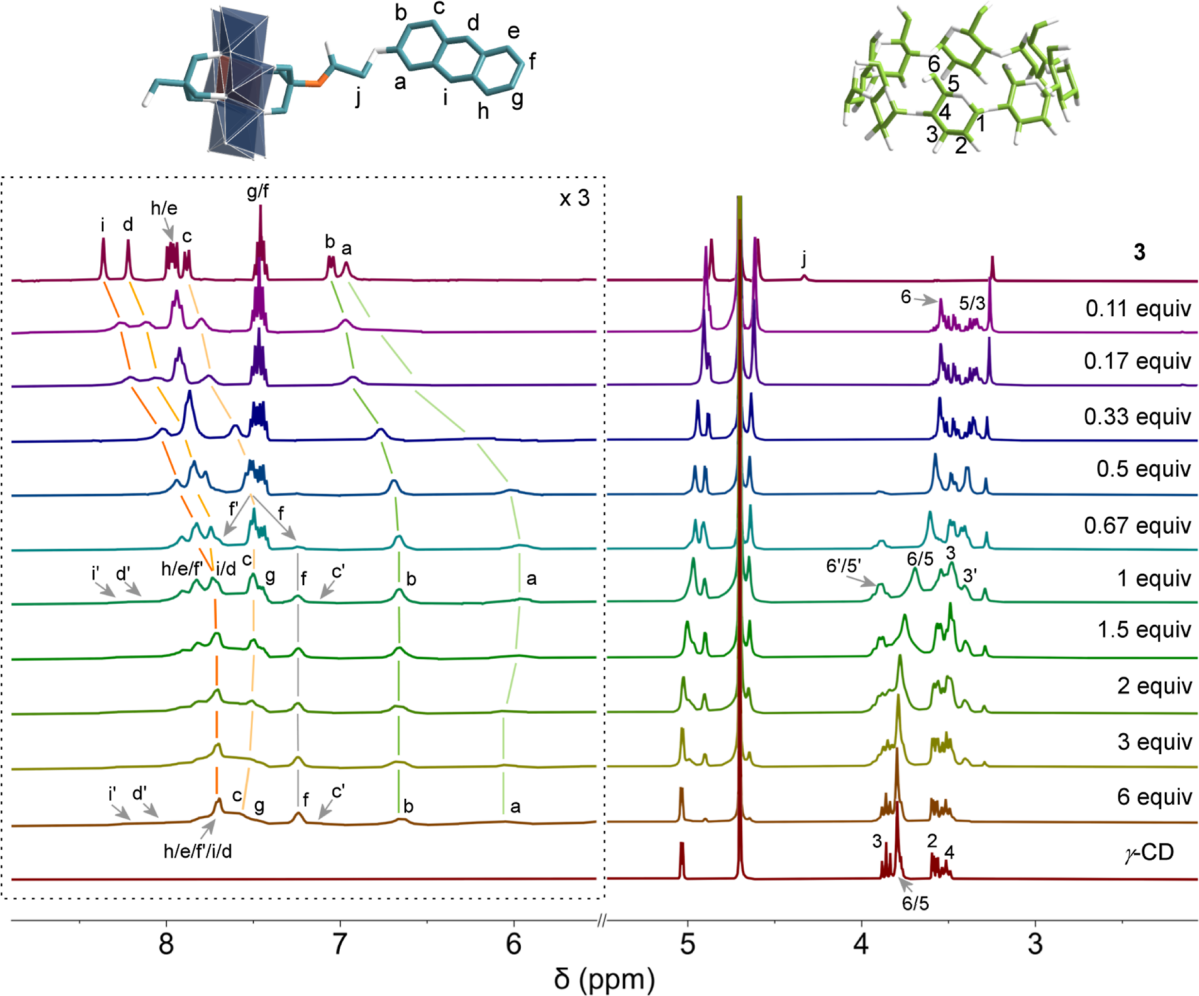
^1^H NMR titration of the supramolecular assembly of γ-CD and **3** upon successive addition of γ-CD into **3** in D_2_O (*c* = 6 mmol/L). The equivalent amounts of γ-CD to **3** are labeled.

Along with the proton shifting and splitting in the aromatic region, the inner γ-CD protons also experienced similar changes. In the first stage, H_3_, H_5_, and H_6_ proton signals of γ-CD all shift upfield relative to free γ-CD in D_2_O. During the second stage, these protons shifted downfield, while H_6_ and H_5_ underwent splitting, generating new resonances at 3.90 ppm (designated 6′/5′, Figure 5). The intensity of these split peaks increased before diminishing as additional γ-CD was introduced (1–6 equiv). In the last stage, with excess γ-CD, all H_3_, H_5_, and H_6_ proton signals returned to their original chemical shifts, matching those of uncomplexed γ-CD.

To better understand the complexation process during titration, we attempted to crystallize assembly intermediates by preparing aqueous mixtures of **3** and γ-CD at 3:1, 1:1, and 1:3 molar ratios. Despite varying the stoichiometry, all crystallization attempts yielded single crystals of 2:2 adducts, mirroring the behavior observed for **2**@γ-CD. X-ray crystallography revealed that **3**@γ-CD adopts a *pseudo*-[4]­rotaxane structure, with both the *Z*- and *E*-isomers of **3** threading through a γ-CD dimer (Figure 6a). The anthracene moieties in both isomers display strong intermolecular interactions with the γ-CD dimer (Figure 6b,c), while also exhibiting close contacts between themselves. As denoted in Figure 6e, key intermolecular distances include C_c_···C_d*_ (3.55 Å) and C_c*_···C_d_ (3.54 Å), with C_f*_ of the *E*-isomer positioned near the *Z*-isomer’s carbonyl group (*d* = 3.56 Å). These structural features explain the H_c_ and H_f_ splitting observed during the second stage of NMR titration, confirming the presence of 2:2 adducts in solution. Additional short contacts between the C_5_/C_6_ atoms of γ-CDs and the oxygen atoms of **3** (Figure 6f,g) account for the observed H_5_/H_6_ splitting in the low-field NMR region. ITC (Figure S41) and UV–vis titration (Figure S42) further confirmed the 1:1 stoichiometry of complex **3**@γ-CD in solution. While previous studies have reported anthracene anchoring on Mn-Anderson clusters^58^ and proposed 2:1 anthracene-γ-CD adducts,^59^ our work provides definitive structural evidence for 2:2 adduct formation, likely attributable to distinct structural features of the Anderson hybrids. Although the anthracene spatial distances are at 9 (C_*d*_···C_d*_) and 10 (C_*i*_···C_i*_) positions (Figure S43) satisfy Schmidt’s criterion, no dimerization of **3**@γ-CD was observed, presumably due to the big twist angle (θ = 39.44°) between anthracene moieties (Figure 6d).

**Figure 6:**
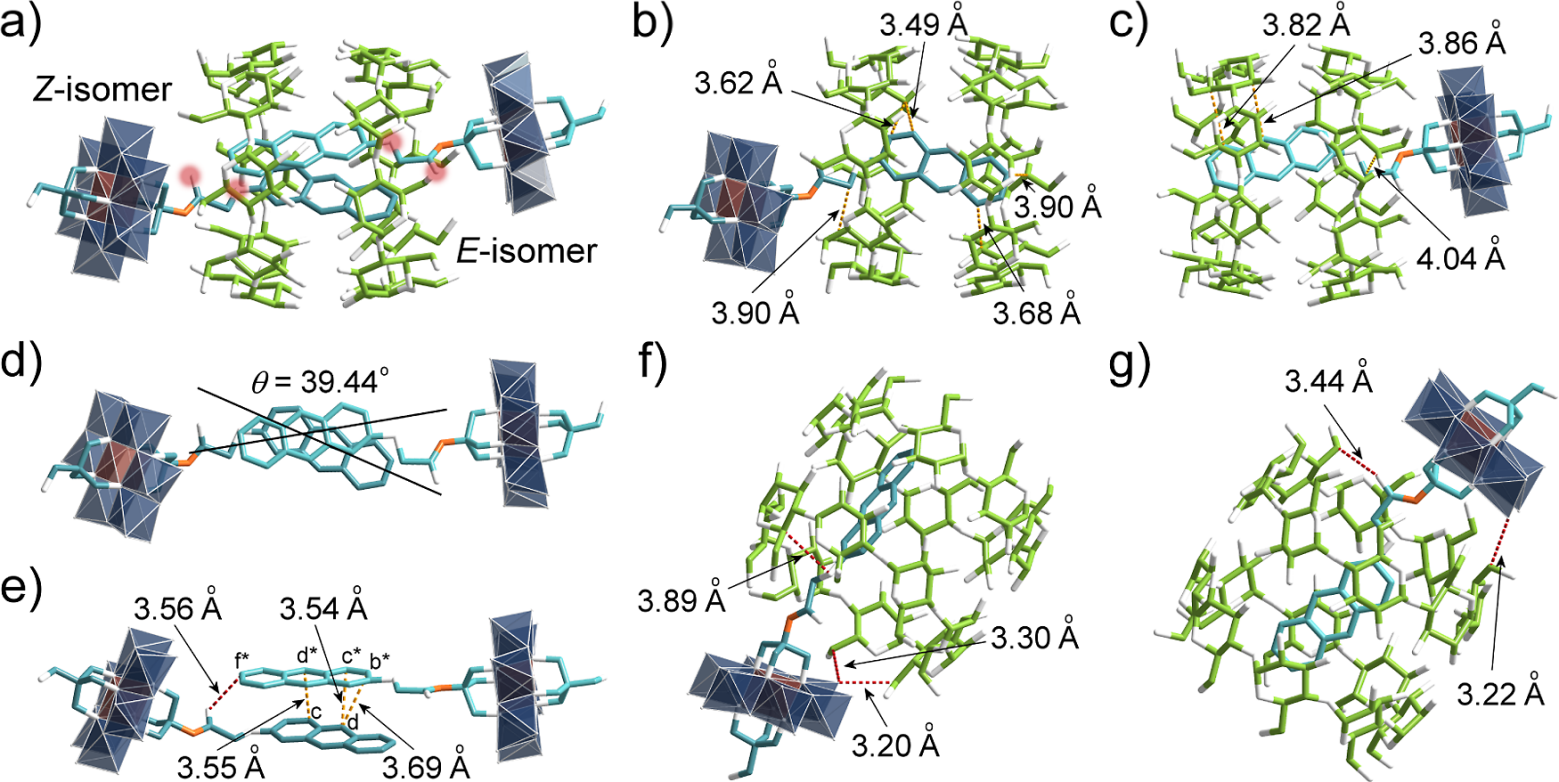
(a) The single-crystal structure of complex **3**@γ-CD, (b) and (c) the intermolecular distances (in yellow dotted lines) between γ-CD and the *Z*- and *E*-isomers of **3**, (d) the twisted angle and (e) the intermolecular distances between the anthracene molecules in complex **3**@γ-CD, and (f and g) the close distances (in red dotted lines) between C_5_/C_6_ of γ-CDs and the oxygen atoms in **3**. Color code: the same as in Figure 2, and carbon in *E*-isomer is represented in cyan.

Based on combined analyses of the ^1^H NMR titration and X-ray data, we proposed the following assembly pathway: initial addition of γ-CD into the solution of **3** generates 2:1 adducts, reaching maximum formation at 0.5 equiv of γ-CD. As the γ-CD ratio increases to 1 equiv, these 2:1 adducts gradually convert to 2:2 complexes. Further γ-CD addition (beyond 1 equiv) promotes formation of new 1:2 species (Figure S38a). 2D NOESY studies at varying molar ratios support this mechanism. For the 3:1 mixture (excess **3**), observed spatial correlations between H_e_-H_a_ and H_i_-H_c_ of anthracene units indicate a significant twisting angle between π-conjugated moieties (Figure S38b). Additional through-space correlations between anthracene (H_i_/H_h_/H_e_/H_c_) and γ-CD (H_3_/H_5_) protons closely resemble those reported for 2:1 adducts of 2-anthracenecarboxylates and γ-CDs.^7^ The 1:1 mixture shows distinct behavior, with only H_d_-H_c’_ spatial correlation between threaded anthracenes (Figure S39), matching the X-ray structure (Figure 6b,c). In the 1:3 mixture (excess γ-CD), the disappearance of intermolecular correlations and appearance of solely *J*3-couplings suggest the inclusion of one anthracene molecule in the cavities of the γ-CD dimer (Figure S38c). ESI-TOF-MS analysis of **3**@γ-CD (Figure S40) further confirms solution-phase equilibria, showing a dominant 1:1 adduct, secondary 1:2 species (similar to the case of **2**@γ-CD), minor 2:1 adducts such as {K_3_·(**3**)_2_@γ-CD}^3–^ and {K_2_·(**3**)_2_@γ-CD}^4–^, and trace 2:2 fragments K_3_·(**3**)_2_@(γ-CD)_2_}^3–^ (Table S2).

We subsequently examined single-sided **3s**, which exhibited nearly identical solution-phase assembly behavior with γ-CD as observed for **3**, as confirmed by multiple characterization techniques (Figures S45–S49). Single crystals of **3s**@γ-CD were obtained through slow evaporation of an aqueous solution containing near-equimolar amounts of **3s** and γ-CD. Unlike the structures of **2**@γ-CD and **3**@γ-CD that contain both *Z*- and *E*-isomers, X-ray diffractions revealed that **3s**@γ-CD crystallizes exclusively with the *Z*-isomer in a 2:2 *pseudo*-[4]­rotaxane arrangement (Figure 7a). This structural distinction likely arises from strong intermolecular hydrogen bonding between γ-CD and the μ_3_-O sites of single-sided **3s**, as evidenced by short O···O contact distances in the crystal packing (Figure S50). The structure also shows close anthracene-γ-CD interactions (Figure S51), consistent with 2D NOESY observations (Figure S46). Importantly, the twist angle between anthracene moieties decreases significantly to 12.61° (Figure 7b), while maintaining favorable C_9_···C_9*_ (3.67 Å) and C_10_···C_10*_ (3.72 Å) distances (Figure S52)—parameters that satisfy both geometric and electronic requirements for successful [4 + 4] photodimerization via single-crystal-to-single-crystal transformation.

**Figure 7:**
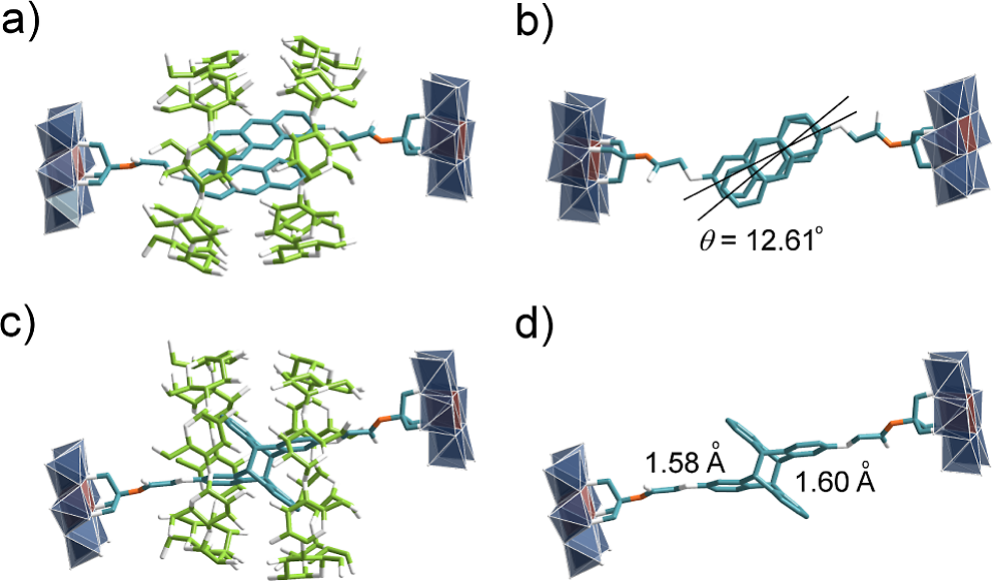
(a) The single-crystal structure of complex **3s**@γ-CD, (b) the twisted angle between the anthracene molecules in complex **3s**@γ-CD, (c) the single-crystal structure of complex **4**@γ-CD, and (d) the bond distances between 9 and 10 positions of the anthracene-dimer in complex **4**@γ-CD. Color code: the same as in Figure 2.

To verify the photodimerization, single crystals of **3s**@γ-CD were coated with Paratone oil and irradiated with 365 nm UV light. Following extensive optimization, high-quality single crystals of **4**@γ-CD were obtained after 8 h of irradiation (Figure S59). X-ray analysis revealed **4**@γ-CD to be a [3]­rotaxane, representing—to our knowledge—the first structurally characterized single-crystal rotaxanes incorporating an anthracene dimer within γ-CDs (Figure 7c). The unit cell of **4**@γ-CD slightly expanded (orthorhombic, *a* = 24.6852(8), *b* = 27.9765(9), *c* = 39.4067(13), *V* = 27214.5(15) Å^3^), compared with that of **3s**@γ-CD (orthorhombic, *a* = 24.7051(5), *b* = 27.7438(6), *c* = 38.6548(10), *V* = 26494.5(10) Å^3^) (Table S3). The cell volume increased by nearly 3% after photodimerization. The bond distances (Figure 7d) between the 9 and 10 positions of anthracene-dimer match those reported for polymerized anthracene 2D polymers.^60^ In a recent report by Shen and Stoddart et al.,^61^ 1-anthracenecarboxylates were incorporated into γ-CD-MOFs for the selective formation of *anti*-head-to-head anthracene dimers in *ca*. 80% *ee* yield. However, crystal structures of such *anti*-head-to-head dimers have not been reported. Here in our system, the constrained geometry of **3s** enforces an exclusive *anti*-head-to-tail alignment in both **3s**@γ-CD and its photoproduct **4**@γ-CD.

The photodimerization was further characterized by ^1^H NMR and 2D NOESY, despite the significantly reduced water solubility of **4**@γ-CD. The ^1^H NMR spectrum (Figure S54) revealed dramatic changes for H_i_ and H_d_ protons, which shifted upfield by Δδ = −3.28 and −3.43 ppm, respectively, moving from the aromatic to aliphatic region. The 2D NOESY spectrum of **4**@γ-CD showed distinct *J*3-couplings between H_i_/H_d_ and their neighboring protons (Figure S55), contrasting sharply with the through-space correlations (H_e_-H_a_ and H_h_-H_b_) observed for **3s**@γ-CD (Figure S46). Solid-state UV–vis spectroscopy confirmed the photodimerization through complete disappearance of the characteristic anthracene absorption at 255 nm and the weak fine absorptions in the range 330–380 nm (Figure S56). Fluorescence studies provided additional evidence with the broad emission band (400–500 nm) of **3s**@γ-CD being completely absent in **4**@γ-CD (Figure S57).

The anthracene dimerization proved to be thermally reversible. Heating bulk samples of **4**@γ-CD at 150 °C for 2 h (Figure S60) regenerated the monomeric form, as evidenced by (i) disappearance of the aliphatic H_i_ and H_d_ signals and (ii) restoration of proton chemical shifts matching those in original **3s**@γ-CD (Figure S61). Recrystallization of the thermally treated material from water successfully regenerated **3s**@γ-CD single crystals, completing the reversible cycle.

## Conclusions

In summary, we successfully demonstrated the supramolecular assembly of γ-CDs with asymmetrically functionalized Anderson-type POMs to construct novel *pseudo*-rotaxane architectures. The covalent modification pattern of Anderson POMs proved to be an effective strategy for designing new POM-CD complexes, enabling the fabrication of a series of organic–inorganic hybrid rotaxanes, including *pseudo-*[2]-, [3]-, and [4]­rotaxane structures. Notably, the *pseudo-*[4]­rotaxanes represent the first single-crystal examples of γ-CD dimers threaded by two organo-POMs. Through photoinduced single-crystal-to-single-crystal transformation, we achieved conversion of these *pseudo*-[4]­rotaxanes to hybrid [3]­rotaxanes featuring: (i) a γ-CD dimer as the wheel, (ii) a photopolymerized anthracene dimer as the axle, and (iii) two Anderson POMs as stoppers. This system also provides the first single-crystal structural characterization of an *anti*-head-to-tail anthracene dimer arrangement within a rotaxane framework. Current efforts in our laboratory are focused on extending this approach to construct 2D/3D MIM frameworks using organo-POM building blocks with the goal of developing functional materials for chiral adsorption, catalysis, and stimuli-responsive applications.

## Supplementary Material



## Notes

Synthetic procedures, NMR, ITC, crystallography, and other materials (PDF)
