# Supplementary material for: Regulating the Assembly of γ‑Cyclodextrin Host and Polyoxometalate-Based Guests toward Light-Responsive Hybrid Rotaxanes
Source: J Am Chem Soc. 2025 Jul 29;147(32):28903–11. doi: 10.1021/jacs.5c06495 (PMC12670418; doi:10.1021/jacs.5c06495)
Supplement: Supplementary file 1 [file ja5c06495_si_001.pdf]

Supporting Information:

Regulating the Assembly of γ-Cyclodextrin Host and Polyoxometalate-Based Guests towards Light-Responsive Hybrid Rotaxanes

Wu-Ji Chen,[†#] Chun-Yan Liu,[†#] Yun-Jing Mu,[†] Yi-An Yin,[†] Chang-Gen Lin,[†*] De-Liang Long,[‡*] Leroy Cronin,[‡*] Yu-Fei Song[†*]

[†]State Key Laboratory of Chemical Resource Engineering, Beijing University of Chemical Technology, Beijing 100029, P. R. China.

E-mail: linchg@mail.buct.edu.cn, songyf@mail.buct.edu.cn

[‡]School of Chemistry, The University of Glasgow, Glasgow G11 6EW, UK.

E-mail: deliang.long@glasgow.ac.uk, lee.cronin@glasgow.ac.uk

[#]These authors contributed equally to this work.

List of Contents:

Section 1. Materials and measurements 3

Section 2. Synthetic procedures and characterizations 4

Section 3. The ^{1}H-NMR, 2D NOESY ^{1}H NMR, Crystal Structures, ESI-TOF-MS, ITC, and TGA of all the hybrid (*pseudo*-)rotaxanes 16

Section 4. The UV-Vis and fluorescence spectra of [3]rotaxane **4**@*γ*-CD 40

Section 5. Single-crystal images of all the hybrid (*pseudo*-)rotaxanes 41

Section 6. The reversible transition of **4**@*γ*-CD to **3s**@*γ*-CD 43

Section 7. Crystallographic data 44

Section 8. References 45

Section 1. Materials and measurements

All the chemicals were analytical grade, purchased from Shanghai Energy Chemical, and used without further purification. $Na_3[Al(OH)_6Mo_6O_{18}]\cdot(H_2O)_6\cdot2H_2O$,[1] $C_6H_5CO(CH)_2C_6H_4OCH_2CO$-$NHC(CH_2OH)_3$ (Chalcone-Tris),[2] $[(n\text{-}C_4H_9)_4N]_3\{Al(OH)_3Mo_6O_{18}[(OCH_2)_3CCH_2OH]\}$ (Al-OH),[3] $Na_3\{Al(OH)_3Mo_6O_{18}[(OCH_2)_3CC_6H_4NO_2]\}$ (Na-**1s**) and $Na_3\{AlMo_6O_{18}[(OCH_2)_3CCH_2OH][(OC$-$H_2)_3CC_6H_4NO_2]\}$ (Na-**1**)[4] were prepared and characterized according to published procedures.

Single-crystal X-ray diffraction data were collected either on a Rigaku Supernova Dual Cu at home/near AtlasS2 diffractometer or a Bruker D8 Venture instrument ($\lambda(Mo_{K\alpha})$ = 0.71073 Å or $\lambda(Cu_{K\alpha})$ = 1.54178 Å). Structure solution and refinement were carried out with SHELXT[5] and SHELXL[6] using Olex2 software package.[7] All the non-hydrogen atoms were anisotropically refined by the full-matrix least-squares method and included in the model. Hydrogen atoms on the carbon atoms were placed in a theoretical riding mode and included in the refinement. Hydrogen atoms on the oxygen atoms of *γ*-CDs and crystalline water molecules were not included in the model.

Fourier transform infrared (FT-IR) spectra were carried out on a Bruker Vector 22 infrared spectrometer using KBr pellet method. Element analyses of C, N and H contents were determined by the elementar UNICUBE instrument. Element analyses of Na, K, Al and Mo contents were measured by inductively coupled plasma optical emission spectrometer (ICP-OES) using a Thermo ICAP PRO instrument. Thermogravimetric (TG) analyses were performed on Mettler Toledo 1/110 SF under N_2 flow with a heating rate of 10 ^oC min^{-1}.

^1H-NMR and 2D NOESY ^1H NMR were recorded on a Bruker AVANCE 400 MHz NMR instrument 24 hours after the sample preparations to guarantee the host-guest exchange equilibrium. Isothermal titration calorimetry (ITC) measurements were performed in degassed deionized water at 25 ^oC. Solutions of *γ*-CD (10 mM) was added into the guest POM solution (1.0 mM) by injecting 2 μL of titrant successively over 4 s with a 180 s interval between injections. The electrospray ionization time-of-flight mass spectroscopy (ESI-TOF-MS) was conducted on a Thermo Scientific Q Exactive mass spectrometer, and all experiments were performed in negative mode. UV-Vis was carried out on a TU-1901 double-beam ultraviolet-visible spectrophotometer. Fluorescence spectra were recorded on a Hitachi F-7000 luminescence spectrometer with a Xe lamp as the excitation source (λ_{ex} = 255 or 365 nm, Ex and Em slit 5 nm).

Section 2. Synthetic procedures and characterizations

Synthesis of $[(n\text{-}C_4H_9)_4N]_3[Al(OH)_6Mo_6O_{18}]$ (TBA-Al)

$Na_3[Al(OH)_6Mo_6O_{18}]\cdot(H_2O)_6\cdot 2H_2O$ (1.8 g, 1.5 mmol), dissolved in 9 mL of H_2O upon heating, was slowly added into the solution of tetrabutylammonium bromide (TBA-Br, 20 g, 62 mmol) in 20 mL of H_2O. The solution was stirred for 15 mins, and microcrystals began to form during this period. The mixture was then left undisturbed at room temperature for slow evaporation. After one day, colorless rod-like crystals were obtained. The product was collected and used without further characterization.

Synthesis of $[(n\text{-}C_4H_9)_4N]_3\{AlMo_6O_{18}[(OCH_2)_3CCH_2OH][(OCH_2)_3CNHCOCH_2OC_6H_4(CH)_2\text{-}COC_6H_5]\}$ (TBA-**2**)

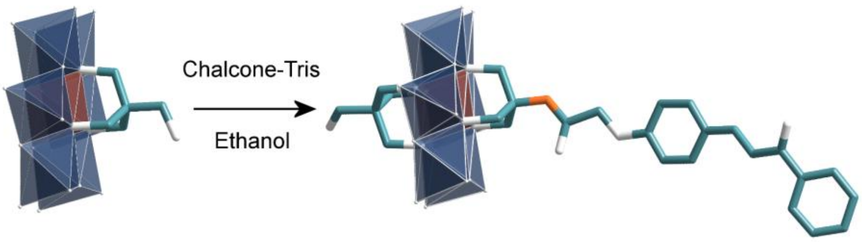

Scheme S1. The synthetic procedure of TBA-**2**. The TBA counter-cations and hydrogens have been omitted for clarity.

Al-OH (1.80 g, 1 mmol) was dissolved in ethanol (60 mL) upon heating and stirring. Chalcone-Tris (0.42 g, 1.1 mmol) was suspended in ethanol (6 mL) and added into the above solution. The resulting mixture was refluxed for 8 hours. The crude product was filtered and dried in air. For purification, the crude product was then dissolved in 40 mL of acetonitrile, and cation-exchanged into sodium salts by adding sodium perchlorate (1.4 g, 12 mmol) into the solution. After stirred for 10 mins, the precipitates were collected by centrifuge and dried in air. The sodium salts of the crude product were dissolved in acetonitrile/water (v/v = 1/1) solution (16 mL), and then TBA-Br (0.3 g, 1.0 mmol) was added. After stirring for 10 mins, the resulting solution was kept at room temperature for slow evaporation for a day. Afterwards, a small portion of crystals were filtered out, and the filtrate was collected. TBA-Br (1.0 g, 3.0 mmol) was added into the filtrate and followed by 80 mL of water. The resulting precipitates were collected, washed with water, and dried in air. Yield: 1.25 g of 59% . ^{1}H-NMR (400 MHz, DMSO-d_6): δ = 8.22 - 8.07 (m, 2H), 7.95 - 7.46 (m, 8H), 6.96 (d, J = 8.7 Hz, 2H), 4.72 (t, J = 5.6 Hz, 1H), 4.65 (s, 6H), 4.49 (s, 2H), 4.37 (d, J = 12.9 Hz, 6H), 3.22 - 3.12 (m, 24H), 2.97 (d, J = 5.5 Hz, 2H), 1.56 (q, J = 8.1, 7.6 Hz, 24H), 1.33 (p, J = 7.3 Hz, 24H), 0.94 (t, J = 7.3 Hz, 36H). ESI-TOF-MS (acetonitrile, neg. mode): m/z = 1891.09 ($\{(TBA)_2\bullet\mathbf{2}\}^{1-}$, *calcd*. 1891.18), 824.33 ($\{(TBA)\bullet\mathbf{2}\}^{2-}$, *calcd*. 824.36). FT-IR (KBr, cm^{-1}): 3472 (s), 3073 (w), 3015 (s), 2921 (m), 2838 (w), 2784 (w), 2735 (w), 2596 (s), 2517 (s), 2493 (s), 2344 (s), 1825 (m), 1645 (m), 1597 (w), 1485 (s), 1399

(m), 1337 (w), 1296 (w), 1223 (m), 1175 (m), 1124 (m), 1074 (w), 1014 (m), 947 (s), 831 (w), 661 (s), 449 (m).

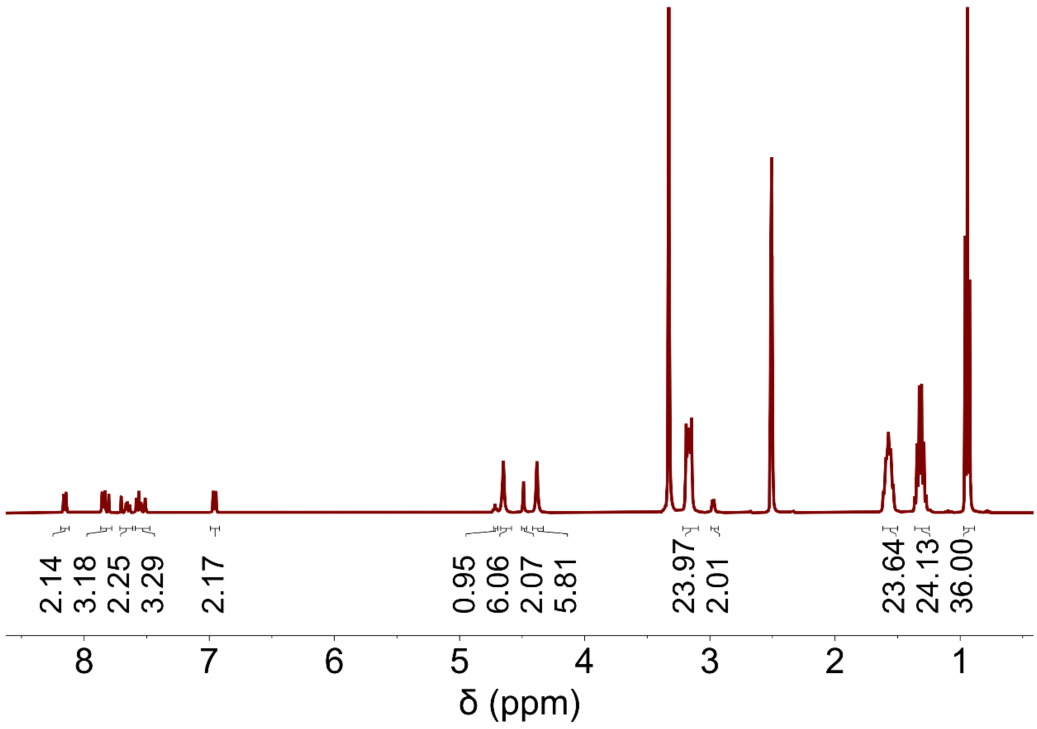

Figure S1. The ^{1}H-NMR spectrum of TBA-**2** in DMSO-d_6.

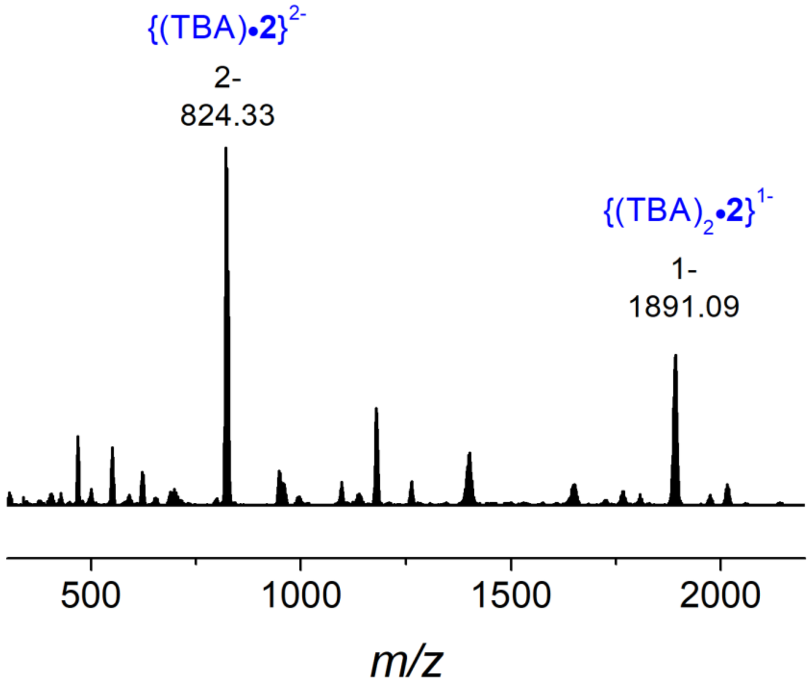

Figure S2. The ESI-TOF-MS spectrum of TBA-**2** in acetonitrile in negative mode.

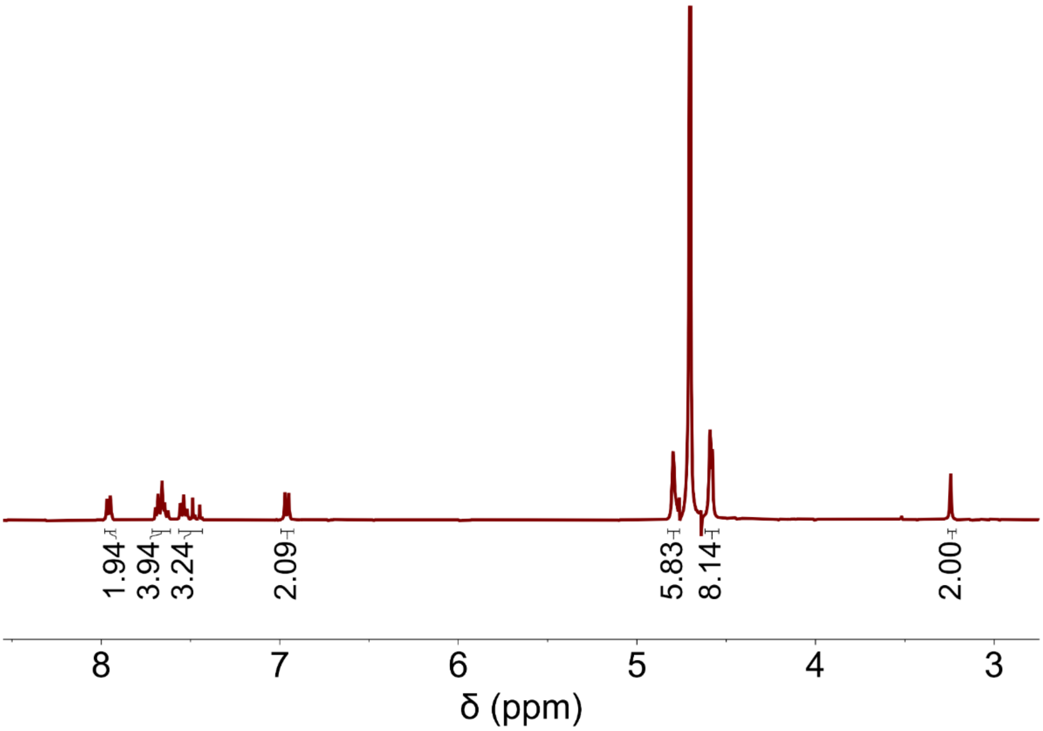

Figure S3. The ^{1}H-NMR spectrum of Na-**2** in D_2O.

Synthesis of $Na_3\{AlMo_6O_{18}[(OCH_2)_3CCH_2OH][(OCH_2)_3CNHCOCH_2OC_6H_4(CH)_2COC_6H_5]\}$ (Na-**2**)

TBA-**2** (2.13 g, 1 mmol) was dissolved in acetonitrile (60 mL). Sodium perchlorate (2.4 g, 20 mmol) in acetonitrile (5 mL) was added into the above solution. After stirred for 10 mins, the precipitates were collected and washed several times by acetonitrile. Yield: 1.0 g of 76%. ^{1}H-NMR (400 MHz, D_2O): δ = 8.02 (d, J =7.4 Hz, 2H), 7.95 - 7.46 (m, 7H), 6.96 (d, J = 6.9 Hz, 2H), 4.80 (m, 6H), 4.29 (m, 8H), 3.19 (s, 2H). FT-IR (KBr, cm^{-1}): 3446 (s), 2930 (w), 2886 (w), 1647 (s), 1595 (m), 1509 (m), 1451 (w), 1339 (w), 1223 (m), 1176 (m), 1123 (m), 1015 (s), 941 (s), 828 (m), 661 (s), 578 (w), 449 (w).

Synthesis of $[(n\text{-}C_4H_9)_4N]_3\{Al(OH)_3Mo_6O_{18}[(OCH_2)_3CNHCOCH_2OC_6H_4(CH)_2COC_6H_5]\}$ (TBA-**2s**)

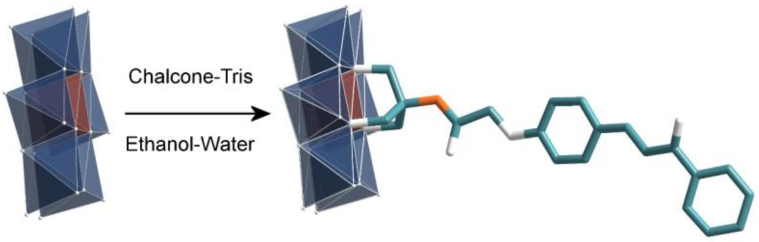

Scheme S2. The synthetic procedure of TBA-**2s**. The TBA counter-cations and hydrogens have been omitted for clarity.

TBA-Al (1.72 g, 1 mmol) was dissolved in ethanol/water (v/v = 4/1) solution (100 mL) with heating and stirring. Chalcone-Tris (0.42 g, 1.1 mmol) suspended in ethanol (6 mL) was added into the above solution. The resulting mixture was heated at reflux for 24 hours. After cooled down to room temperature, the white precipitates were filtered off and the filtrate was evaporated. The resulting residue was washed several times using THF, and then re-dissolved in a mixture of acetonitrile (20 mL) and water (40 mL). The undissolved solids were removed by centrifuge, and the resulting solution was evaporated to give pure products. Yield: 0.92 g of 45%. ^{1}H-NMR (400 MHz, DMSO-d_6): δ = 8.20 - 8.11 (m, 2H), 7.87 - 7.79 (m, 3H), 7.74 - 7.51 (m, 5H), 7.40 (s, 1H), 6.97 (d, J = 8.7 Hz, 2H), 4.66 (s, 6H), 4.49 (s, 2H), 3.57 (s, 3H), 3.21 - 3.06 (m, 24H), 1.56 (q, J = 8.2, 7.6 Hz, 24H), 1.32 (h, J = 7.4 Hz, 24H), 0.94 (t, J = 7.3 Hz, 36H). FT-IR (KBr, cm^{-1}): 3474 (s), 3069 (m), 3015 (m), 2953 (m), 2785 (m), 2463 (m), 2344 (m), 1830 (m), 1649 (m), 1559 (m), 1487 (s), 1399 (m), 958 (s), 661 (s), 447 (m).

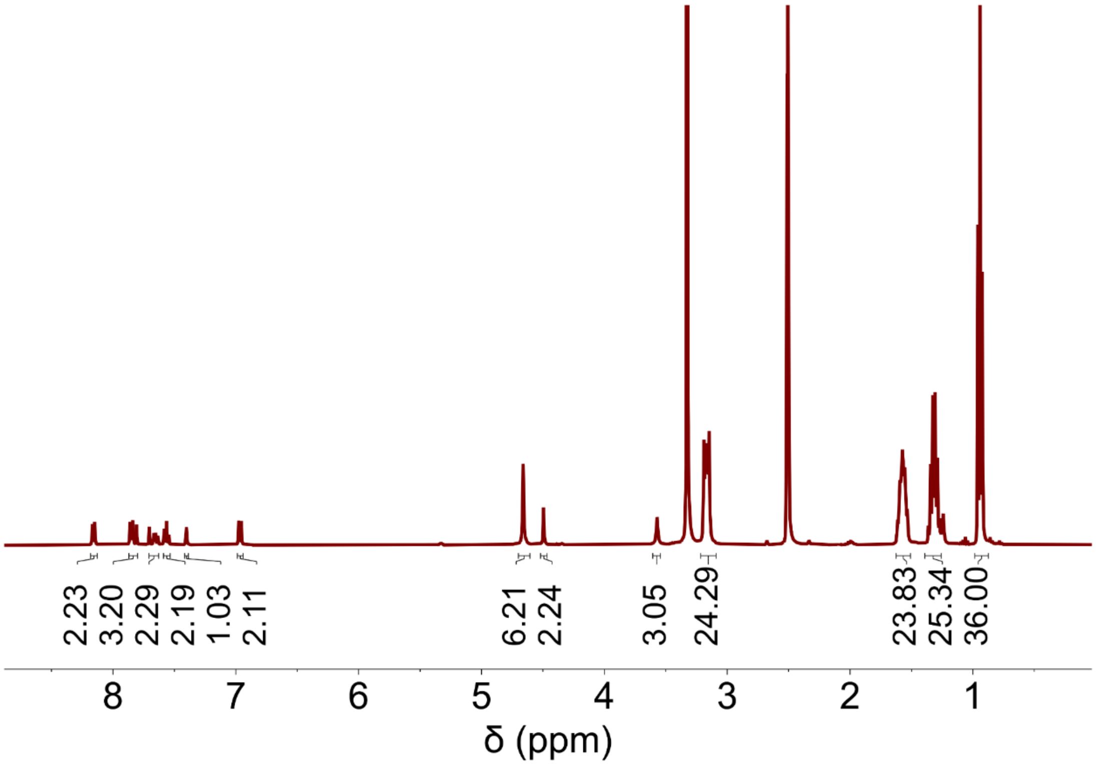

Figure S4. The ^{1}H-NMR spectrum of TBA-**2s** in DMSO-d_6.

Synthesis of $Na_3\{Al(OH)_3Mo_6O_{18}[(OCH_2)_3CNHCOCH_2OC_6H_4(CH)_2COC_6H_5]\}$ (Na-**2s**)

TBA-**2s** (2.13 g, 1 mmol) was dissolved in acetonitrile (60 mL) with stirring. Sodium perchlorate (2.4 g, 20 mmol) in 5 mL of water was added to the solution. After stirred for 10 mins, the precipitates were collected, and washed several times by acetonitrile. Yield: 1.04 of 76%. ^{1}H-NMR (400 MHz, DMSO-d_6): δ = 8.20 - 8.11 (m, 2H), 7.87 - 7.79 (m, 3H), 7.74 - 7.51 (m, 5H), 7.40 (s, 1H), 6.97 (d, J = 8.7 Hz, 2H), 4.66 (s, 6H), 4.49 (s, 2H), 3.57 (s, 3H). FT-IR (KBr, cm^{-}

1): 3428 (s), 1647 (w), 1595 (m), 1508 (m), 1223 (m), 1076 (m), 931 (s), 661 (s), 578 (w), 451 (m).

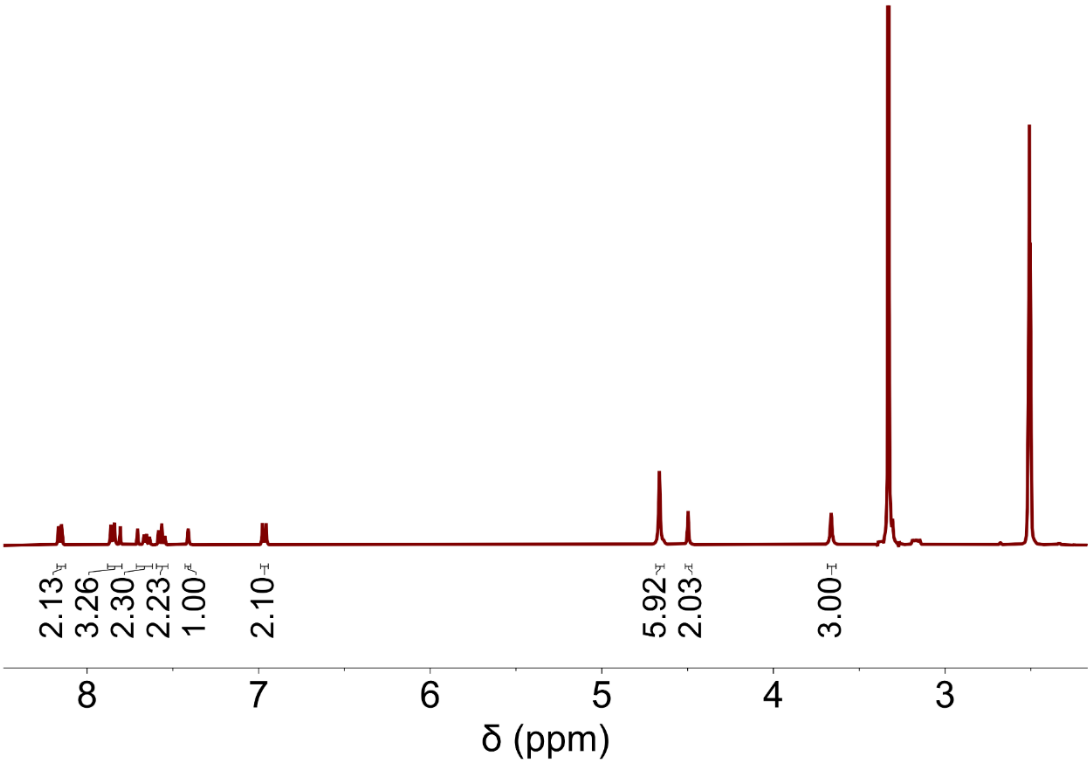

Figure S5. The ^{1}H-NMR spectrum of Na-**2s** in DMSO-*d*6.

Synthesis of $C_{14}H_9OCH_2COOCH_2CH_3$

Anthracen-2-ol (1.94 g, 10 mmol) was dissolved in acetonitrile (80 mL) with heating and stirring. K_2CO_3 (1.64 g, 12 mmol) and KI (0.08 g, 0.5mmol) was then added, followed by slowly adding of ethyl bromoacetate (2.0 g, 12 mmol). The resulting mixture was heated at reflux for 24 hours. After cooled down to room temperature, the precipitates were removed by centrifuge and the resulting solution was evaporated, and the residue was washed several times by hexane. Yield: 2.38 g of 85%. ^{1}H-NMR (400 MHz, DMSO-d_6): δ = 8.52 (s, 1H), 8.39 (s, 1H), 8.04 (t, J = 8.6 Hz, 3H), 7.48 (m, 2H), 7.36 (d, J = 2.5 Hz, 1H), 7.26 (dd, J = 9.2, 2.5 Hz, 1H), 4.96 (s, 2H), 4.23 (q, J = 7.1 Hz, 2H), 1.25 (t, J = 7.1 Hz, 3H). FT-IR (KBr, cm^{-1}): 3438 (m), 2982 (m), 1759 (s), 1626 (s), 1433 (m), 1268 (m), 1201 (s), 1072 (m), 1018 (s), 881 (s), 810 (w), 742 (s), 594 (m), 471 (m).

Synthesis of $C_{14}H_9OCH_2CONHC(CH_2OH)_3$ (Anthracene-Tris)

$C_{14}H_9OCH_2COOCH_2CH_3$ (0.28 g, 1 mmol), Tris (0.13 g, 1.1 mmol), and K_2CO_3 (0.16 g, 1.1 mmol) were suspended in dry DMSO (10 mL) and stirred at room temperature for 24 hours under nitrogen. After removing the solid by centrifuge, the supernatant was evaporated under reduced pressure, and washed several times by water and ethanol. Yield: 0.31 g of 88%. ^{1}H-

NMR(DMSO-d_6): δ = 3.60 (d, 6H), 4.58 (s, 2H), 4.76 (t, 3H), 7.06 (d, 2H), 7.23 (s, 1H), 7.58 (t, 2H), 7.69 (t, 1H), 7.73 (d, 1H), 7.84 (d, 1H), 7.88 (d, 2H), 8.15 (d, 2H). FT-IR (KBr, cm^{-1}): 3372 (s), 2936 (m), 2624 (m), 1663 (s), 1537 (m), 1408 (s), 1271 (w), 1210 (s), 1061 (m), 1010 (m), 889 (m), 835 (m), 745 (m), 702 (m), 594 (w), 468 (m).

Synthesis of $[(n\text{-}C_4H_9)_4N]_3\{AlMo_6O_{18}[(OCH_2)_3CCH_2OH][(OCH_2)_3CNHCOCH_2OC_{14}H_9]\}$ (TBA-**3**)

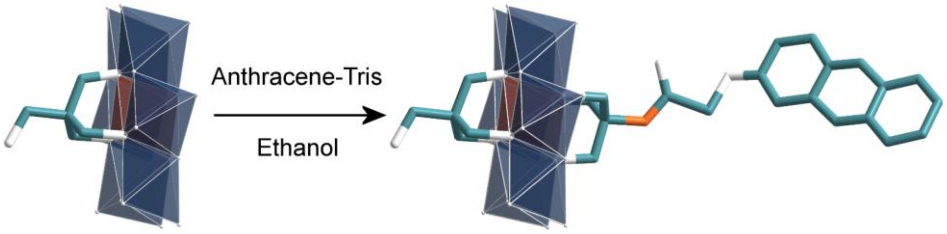

Scheme S3. The synthetic procedure of TBA-**3**. The TBA counter-cations and hydrogens have been omitted for clarity.

Al-OH (1.80 g, 1 mmol) was dissolved in ethanol (60 mL), and heated to reflux for 30 mins. Anthracene-Tris (0.39 g, 1.1 mmol) suspended in ethanol (6 mL) was then added. The resulting solution was heated at reflux for 6 hours. After cooled down to room temperature, the crude product was filtered off and dried in air. For purification, the crude product was dissolved in acetonitrile/water (v/v = 3/1, 150 mL), and any undissolved solids were removed by centrifuge. Pure product was obtained after rotary evaporation of the acetonitrile/water mixture. Yield: 1.30 g of 62%. ^{1}H-NMR (400 MHz, DMSO-d_6): δ = 8.48 (s, 1H), 8.39 (s, 1H), 8.01 (t, J = 8.8 Hz, 3H), 7.55 (s, 1H), 7.49 - 7.40 (m, 2H), 7.29 - 7.23 (m, 2H), 4.75 (d, J = 5.7 Hz, 1H), 4.70 (s, 6H), 4.55 (s, 2H), 4.39 (s, 6H), 3.22 - 3.09 (m, 24H), 2.98 (d, J = 5.6 Hz, 2H), 1.67 - 1.47 (m, 24H), 1.31 (h, J = 7.3 Hz, 24H), 0.93 (t, J = 7.3 Hz, 36H). ESI-TOF-MS (acetonitrile, neg. mode): *m*/*z* = 1861.06 ($\{(TBA)_2\bullet\mathbf{3}\}^{1-}$, *calcd*. 1861.15), 809.44 ($\{(TBA)\bullet\mathbf{3}\}^{2-}$, *calcd*. 809.60). FT-IR (KBr, cm^{-1}): 3401 (s), 2960 (m), 2874 (m), 1699 (w), 1636 (w), 1478 (m), 1379 (w), 1207 (w), 1132 (w), 1070 (w), 1020 (m), 931 (s), 669 (s), 573 (w), 470 (w).

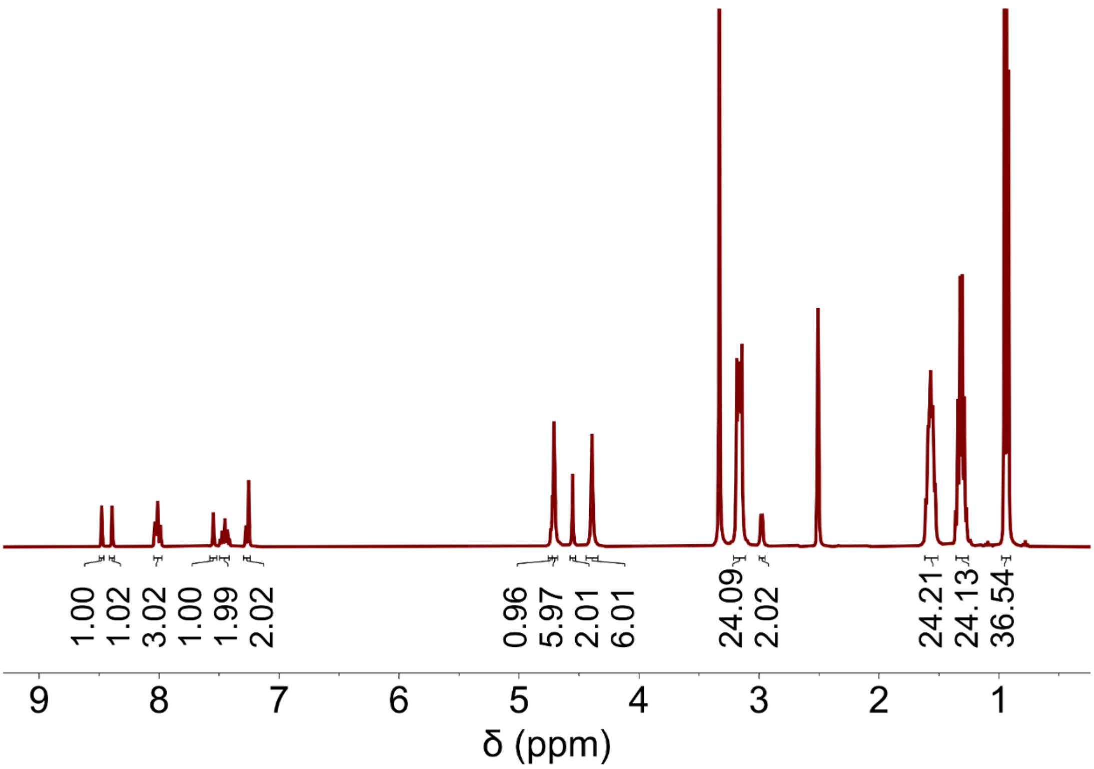

Figure S6. The ^{1}H-NMR spectrum of TBA-**3** in DMSO-d_6.

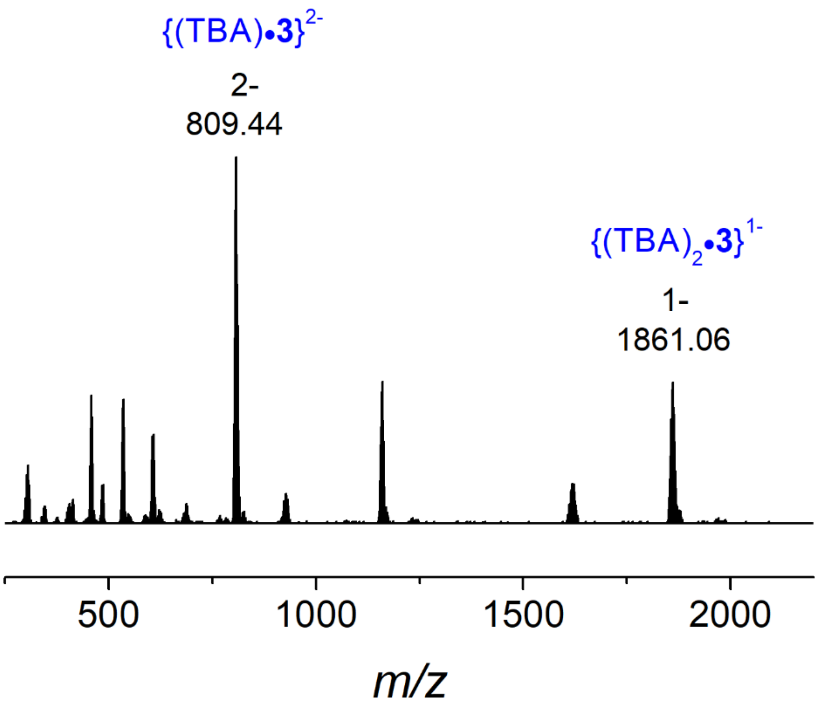

Figure S7. The ESI-TOF-MS spectrum of TBA-**3** in acetonitrile in negative mode.

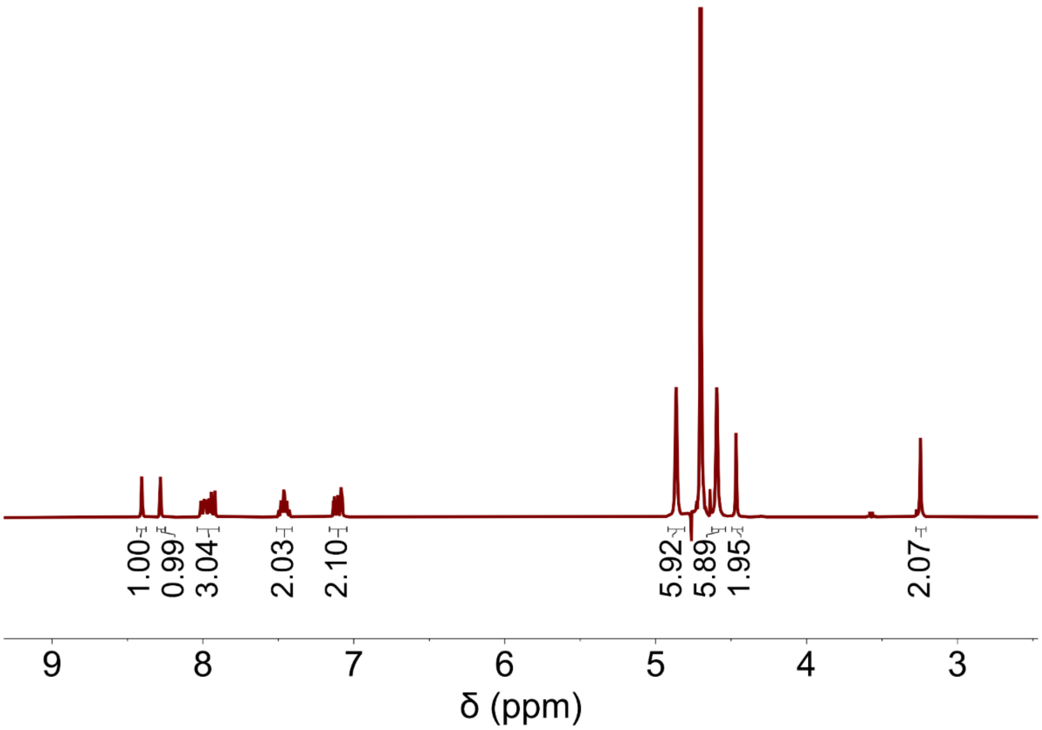

Figure S8. The ^{1}H-NMR spectrum of Na-**3** in D_2O.

Synthesis of $Na_3\{AlMo_6O_{18}[(OCH_2)_3CCH_2OH][(OCH_2)_3CNHCOCH_2OC_{14}H_9\}$ (Na-**3**)

TBA-**3** (2.10 g, 1 mmol) was dissolved in acetonitrile (60 mL) with stirring. Sodium perchlorate (2.4 g, 20 mmol) in acetonitrile (5 mL) was added drop-wise into the above solution. After stirred for 10 mins, the precipitates were collected and washed several times by acetonitrile. Yield: 1.07g of 74%. ^{1}H-NMR (400 MHz, D_2O): δ = 8.40 (s, 1H), 8.31 (s, 1H), 8.04 - 7.92 (m, 3H), 7.46 (m, 2H), 7.14 - 7.02 (m, 2H), 4.86 (s, 6H), 4.59 (s, 6H), 4.44 (s, 2H), 3.21 (s, 2H). FT-IR (KBr, cm^{-1}): 3453 (s), 2924 (w), 1628 (m), 1538 (w), 1464 (w), 1201 (w) 1122 (w), 1012 (m), 941 (s), 663 (s), 468 (m).

Synthesis of $[(n\text{-}C_4H_9)_4N]_3\{Al(OH)_3Mo_6O_{18}[(OCH_2)_3CNHCOCH_2OC_{14}H_9\}$ (TBA-**3s**)

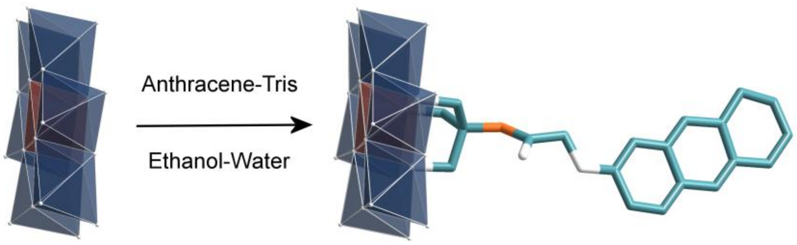

Scheme S4. The synthetic procedure of TBA-**3s**. The TBA counter-cations and hydrogens have been omitted for clarity.

TBA-Al (1.72 g, 1 mmol) was dissolved in ethanol/water (4/1) solution (100 mL) with heating and stirring. Anthracene-Tris (0.39 g, 1.1 mmol) suspended in ethanol (6 mL) was added into

the above solution. The resulting mixture was refluxed for 24 hours. After cooled down to room temperature, the precipitate was removed by centrifuge and the supernatant was evaporated, and washed several times by THF, and water/acetonitrile (v/v = 3/1) mixture consecutively to give the pure product. Yield: 0.80 g of 40%. ^{1}H-NMR (400 MHz, DMSO-d_6): δ = 8.48 (s, 1H), 8.40 (s, 1H), 8.06 - 7.98 (m, 3H), 7.50 - 7.41 (m, 3H), 7.27 (d, J = 8.4 Hz, 2H), 4.72 (s, 6H), 4.56 (s, 2H), 3.57 (s, 3H), 3.24 - 3.08 (m, 24H), 1.57 (p, J = 8.7, 7.8 Hz, 24H), 1.32 (h, J = 7.4 Hz, 24H), 0.94 (t, J = 7.3 Hz, 36H). FT-IR (KBr, cm^{-1}): 3447 (s), 2961 (w), 1632 (m), 1458 (w), 1265 (w), 1076 (m), 928 (s), 661 (s), 453 (w).

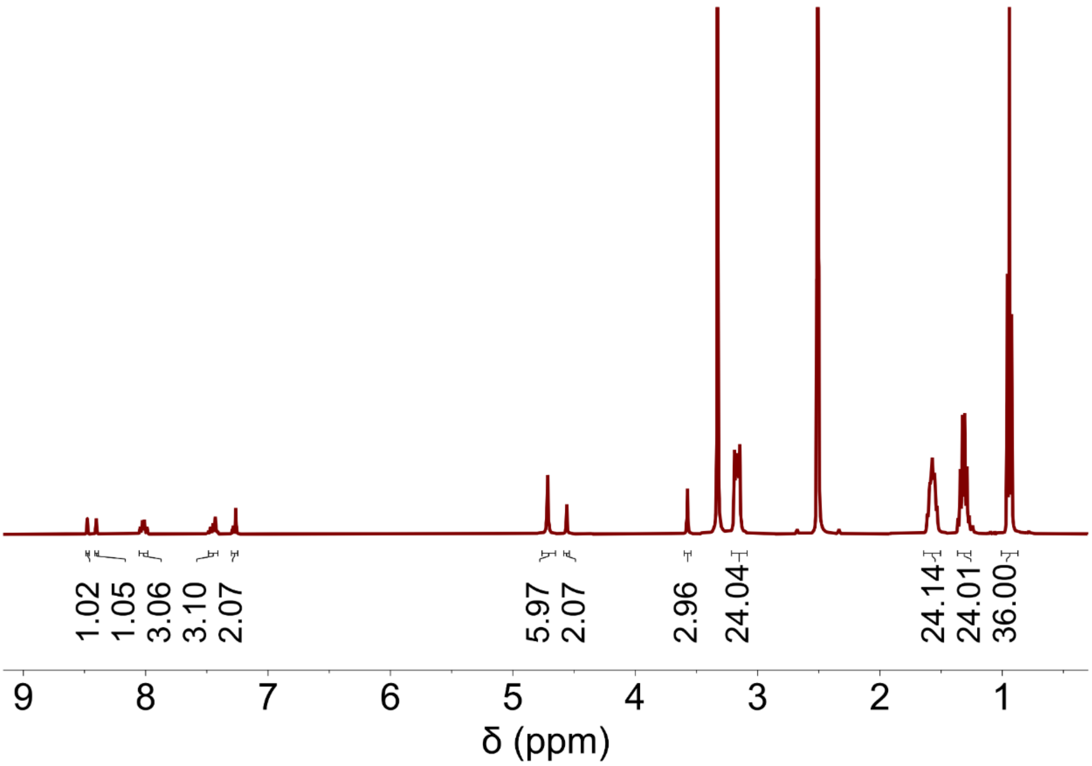

Figure S9. The ^{1}H-NMR spectrum of TBA-**3s** in DMSO-d_6.

Synthesis of $Na_3\{Al(OH)_3Mo_6O_{18}[(OCH_2)_3CNHCOCH_2OC_{14}H_9]\}$ (Na-**3s**)

TBA-**3s** (2.10 g, 1 mmol) was dissolved in acetonitrile (60 mL) with stirring. Sodium perchlorate (2.4 g, 20 mmol) in water (5 mL) was added drop-wise. After stirred for 10 mins, the precipitate was collected by centrifuge, and washed several times using acetonitrile. Yield: 0.98 g of 71%. ^{1}H-NMR (400 MHz, DMSO-d_6): δ = 8.48 (s, 1H), 8.40 (s, 1H), 8.06 - 7.98 (m, 3H), 7.50 - 7.41 (m, 3H), 7.27 (d, J = 8.4 Hz, 2H), 4.72 (s, 6H), 4.56 (s, 2H), 3.57 (s, 3H). FT-IR (KBr, cm^{-1}): 3451 (s), 2934 (w), 1630 (m), 1537 (w), 1456 (w), 1267 (w), 1205 (w), 1072 (m), 918 (s), 655 (s), 455 (m).

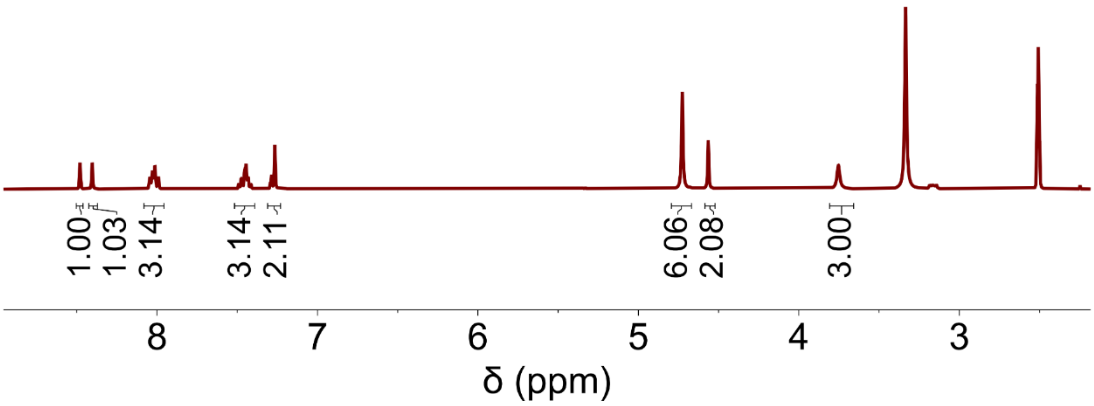

Figure S10. The ^{1}H-NMR spectrum of Na-**3s** in DMSO-*d*$_6$.

Synthesis of $K_3(C_{48}H_{80}O_{40})\{AlMo_6O_{18}[(OCH_2)_3CCH_2OH][(OCH_2)_3CC_6H_4NO_2]\}$ (**1**@*γ*-CD)

Na-**1** (14 mg, 10 *μ*mol) was dissolved in 800 *μ*L of acetonitrile/water (v/v = 1/1) at 40 °C. To this solution *γ*-CD (32 mg, 25 *μ*mol) and KCl (48 mg, 650 *μ*mol) was added. The resulting mixture was kept at room temperature for slow evaporation. Colorless plate crystals were obtained after one day (Figure S58). Yield: 10 mg of 39%. ^{1}H-NMR (400 MHz, D_2O): *δ* = 8.21 - 8.18 (m, 2H), 7.62 - 7.59 (m, 2H), 5.03 (d, *J* = 3.9 Hz, 8H), 4.94 (s, 6H), 4.64 (d, *J* = 2.5 Hz, 6H), 3.86 (t, *J* = 9.6 Hz, 8H), 3.78 (d, *J* = 9.0 Hz, 24H), 3.58 (dd, *J* = 9.9, 3.8 Hz, 8H), 3.51 (t, *J* = 9.5 Hz, 8H), 3.27 (s, 2H). ESI-TOF-MS (H_2O, neg. mode): *m*/*z* = 1292.39 ($\{K \bullet \mathbf{1}@\gamma\text{-CD}\}^{2-}$, *calcd*. 1292.13), 1288.40 ($\{K \bullet H \bullet \mathbf{1}\}^{1-}$, *calcd*. 1288.40), 1249.44 ($\{H_2 \bullet \mathbf{1}\}^{1-}$, *calcd*. 1250.05), 848.61 ($\{\mathbf{1}@\gamma\text{-CD}\}^{3-}$, *calcd*. 848.39). FT-IR (KBr, cm^{-1}): 3422 (s), 2928 (m), 1630 (m), 1516 (m), 1456 (w), 1354 (m), 1159 (w), 1122 (w), 1016 (m), 933 (s), 856 (w), 667 (s), 576 (w), 528 (w), 447 (w). Elemental analysis (%) calcd for $C_{63}H_{99}AlK_3Mo_6NO_{67}$ with 8 H_2O solvent molecules: K 4.18, Al 0.96, Mo, 20.51, C 26.96, H 4.12, N 0.50; found: K 3.86, Al 0.87, Mo, 21.42, C 27.78, H 4.06, N 0.43.

Synthesis of $Na_{15}(C_{48}H_{80}O_{40})_4\{Al(OH)_3Mo_6O_{18}[(OCH_2)_3CC_6H_4NO_2]\}_5$ (**1s**@*γ*-CD)

Na-**1s** (14 mg, 10 *μ*mol), NaCl (15 mg, 250 *μ*mol), and *γ*-CD (16 mg, 12 *μ*mol) were dissolved in 400 mL of water. The resulting solution was kept at room temperature for slow evaporation. Colorless flake crystals began to form after 3 days. Yield: 13 mg of 47%. ^{1}H-NMR (400 MHz, D_2O): *δ* = 8.22 - 8.18 (m, 2.5H), 7.65 - 7.61 (m, 2.5H), 5.03 (d, *J* = 4.1 Hz, 8H), 4.96 (s, 7.5H), 3.86 (t, *J* = 9.5 Hz, 8H), 3.78 (d, *J* = 8.4 Hz, 24H), 3.58 (dd, *J* = 9.9, 3.8 Hz, 8H), 3.55 - 3.47 (m, 8H). ESI-TOF-MS (H_2O, neg. mode): *m*/*z* = 1665.16 ($\{Na_3 \bullet (\mathbf{1s})_2@(\gamma\text{-CD})_2\}^{3-}$, *calcd*. 1665.04),

1253.41 ({**1s**@(γ-CD)$_2$}$^{3-}$, *calcd.* 1253.40), 1243.40 ({Na•**1s**@γ-CD}$^{2-}$, *calcd.* 1243.02), 1232.75 ({Na$_3$•(**1s**)$_2$@γ-CD}$^{3-}$, *calcd.* 1232.67), 1232.40 ({H•**1s**@γ-CD}$^{2-}$, *calcd.* 1232.03), 820.60 (**1s**@γ-CD}$^{3-}$, *calcd.* 821.02). FT-IR (KBr, cm^{-1}): 3395 (s), 2930 (m), 1634 (m), 1545 (w), 1460 (w), 1419 (w), 1377 (w), 1333 (w), 1261 (w), 1157 (m), 1026 (s), 937 (s), 657 (s), 576 (w), 530 (w), 470 (w). Elemental analysis (%) calcd for $C_{242}H_{385}Al_5Mo_{30}O_{290}N_5Na_{15}$ with 50 H_2O solvent molecules: Na 2.81, Al 1.10, Mo 23.47, C 23.70, H 3.99, N 0.57; found: Na 2.95, Al 1.16, Mo 25.26, C 24.50, H 4.41, N 0.48.

Synthesis of $K_3(C_{48}H_{80}O_{40})\{AlMo_6O_{18}[(OCH_2)_3CCH_2OH][(OCH_2)_3CNHCOCH_2OC_6H_4(CH)_2$-$COC_6H_5]\}$ (**2**@γ-CD)

Na-**2** (15 mg, 10 μmol) was dissolved in 500 μL of water at 40 °C. To this solution, γ-CD (130 mg, 100 μmol) and KCl (100 mg, 1.34 mmol) dissolved in 1.5 mL of water was added. Finally, 200 μL of acetonitrile was added, and the resulting solution was kept at room temperature for slow evaporation. Pale-yellow rod crystals were obtained after one day. Yield: 11 mg of 39%. ^{1}H-NMR (400 MHz, D_2O): δ = 7.62 (m, 3H), 7.64 (m, 2H), 7.27 - 7.19 (m, 3H), 6.66 (m, 3H), 4.95 (m, 8H), 4.80 (m, 6H), 4.58 - 4.55 (m, 6H), 3.93 (br, 2H), 3.72 - 3.64 (m, 32H), 3.52 - 3.44 (m, 18H), 3.23 (d, 2H). ESI-TOF-MS (H_2O, neg. mode): *m/z* = 2019.66 ({K•**2**@(γ-CD)$_2$}$^{2-}$, *calcd.* 2019.79), 1371.44 ({K•**2**@γ-CD}$^{2-}$, *calcd.* 1371.23), 1333.45 ({**2**@(γ-CD)$_2$}$^{3-}$, *calcd.* 1333.49), 901.31 ({**2**@γ-CD}$^{3-}$, *calcd.* 901.12). FT-IR (KBr, cm^{-1}): 3390 (vs), 2922 (m), 1663 (m), 1417 (m), 1335 (m), 1151 (s), 1024 (s), 924 (s), 670 (s), 582 (m), 461 (w). Elemental analysis (%) calcd. for $C_{74}H_{109}AlK_3Mo_6O_{68}N$ with 12 H_2O solvent molecules: K 3.86, Al 0.89, Mo 18.96, C 29.27, H 4.41, N 0.46; found: K 3.67, Al 0.84, Mo 19.42, C 28.98, H 4.11, N 0.72.

Synthesis of $K_3(C_{48}H_{80}O_{40})\{Al(OH)_3Mo_6O_{18}[(OCH_2)_3CNHCOCH_2OC_6H_4(CH)_2COC_6H_5]\}$ (**2s**@γ-CD)

Na-**2s** (14 mg, 10 μmol) was dissolved in 500 μL of water at 40 °C. To this solution γ-CD (16 mg, 12.3 μmol) and KCl (15 mg, 200 μmol) in 340 μL of water were added. The resulting mixture was kept at room temperature for slow evaporation. Pale-yellow plate crystals were obtained after one day. Yield: 15 mg of 56%. ^{1}H-NMR (400 MHz, D_2O): δ = 7.69 (m, 3H), 7.54 (m, 3H), 7.35 - 7.28 (m, 3H), 6.90 (m, 1H), 6.82 (m, 2H), 5.01 (m, 8H), 4.88 (s, 6H), 4.07 (br, 2H), 3.78 - 3.67 (m, 32H), 3.57 - 3.48 (m, 16H). ESI-TOF-MS (H_2O, neg. mode): *m/z* = 1979.68 ({Na•**2s**@(γ-CD)$_2$•H_2O}$^{2-}$, *calcd.* 1979.69), 1970.64 ({Na•**2s**@(γ-CD)$_2$}$^{2-}$, *calcd.* 1970.69), 1959.66 ({H•**2s**@(γ-CD)$_2$}$^{2-}$, *calcd.* 1959.70), 1738.57 ({**2s**@(γ-CD)$_3$}$^{3-}$, *calcd.* 1738.51), 1330.42 ({K•**2s**@γ-CD}$^{2-}$, *calcd.* 1330.18), 1311.44 ({H•**2s**@γ-CD)}$^{2-}$, *calcd.* 1311.13), 1306.44 ({**2s**@(γ-CD)$_2$}$^{2-}$, *calcd.* 1306.13). FT-IR (KBr, cm^{-1}): 3309 (vs), 2924 (m), 1657 (m), 1600 (m), 1513 (w), 1426 (m), 1225 (s), 1018 (s), 931 (s), 661 (s), 442 (m). Elemental analysis (%) calcd for $C_{69}H_{103}AlK_3Mo_6O_{67}N$ with 8 H_2O solvent molecules: K 4.07, Al 0.94, Mo 19.96, C 28.75, H 4.16, N 0.49; found: K 3.88, Al 0.86, Mo 20.21, C 28.64, H 4.65, N 0.52.

Synthesis of $Na_{0.5}K_{2.5}(C_{48}H_{80}O_{40})\{AlMo_6O_{18}[(OCH_2)_3CCH_2OH][(OCH_2)_3CNHCOCH_2OC_{14}H_9]\}$ (**3**@*γ*-CD)

Na-**3** (30 mg, 20 *μ*mol) was dissolved in 500 *μ*L of water at 40 °C. To this solution *γ*-CD (26 mg, 20 *μ*mol) and KCl (15 mg, 200 *μ*mol) in 500 *μ*L water was added. The resulting mixture was kept at room temperature for slow evaporation. Light-brown rod/block crystals (Figure S58) began to form after one day. Yield: 34 mg of 51%. ^{1}H-NMR (400 MHz, D_2O): *δ* = 8.02 - 7.66 (m, 4H), 7.66 - 7.02 (m, 3H), 6.67 (br, 1H), 5.90 (br, 1H), 4.96 - 4.90 (m, 14H), 4.64 (s, 6H), 3.96 - 3.40 (m, 50H), 3.28 (m, 2H). ESI-TOF-MS (H_2O, neg. mode): *m/z* = 2004.64 ($\{K•\mathbf{3}@(\gamma\text{-CD})_2\}^{2-}$, *calcd*. 2004.78), 1821.26 ($\{K_3•(\mathbf{3})_2@(\gamma\text{-CD})_2\}^{3-}$, *calcd*. 1821.32), 1755.90 ($\{\mathbf{3}@(\gamma\text{-CD})_3\}^{3-}$, *calcd*. 1755.86), 1388.46 ($\{K_3•(\mathbf{3})_2@\gamma\text{-CD}\}^{3-}$, *calcd*. 1388.95), 1356.47 ($\{K•\mathbf{3}@\gamma\text{-CD}\}^{2-}$, *calcd*. 1356.22), 1337.48 ($\{H•\mathbf{3}@\gamma\text{-CD}\}^{2-}$, *calcd*. 1337.46), 1323.46 ($\{\mathbf{3}@(\gamma\text{-CD})_2\}^{3-}$, *calcd*. 1323.49), 1031.82 ($\{K_2•(\mathbf{3})_2@\gamma\text{-CD}\}^{4-}$, *calcd*. 1031.94), 890.97 ($\{\mathbf{3}@\gamma\text{-CD}\}^{3-}$, *calcd*. 891.11). FT-IR (KBr, cm^{-1}): 3397 (s), 2930 (m), 1632 (m), 1541 (w), 1456 (w), 1337 (w), 1263 (w), 1157 (w), 1024 (s), 937 (s), 663 (s), 580 (w), 474 (m). Elemental analysis (%) calcd for $C_{73}H_{107}AlMo_6O_{67}NNa_{0.5}K_{2.5}$ with 6 H_2O solvent molecules: Na 0.40, K 3.38, Al 0.93, Mo 19.92, C 30.33, H 4.15, N 0.48; found: Na 0.24, K 3.78, Al 0.88, Mo 20.84, C 29.95, H 4.22, N 0.54.

Synthesis of $Na_3(C_{48}H_{80}O_{40})\{Al(OH)_3Mo_6O_{18}[(OCH_2)_3CNHCOCH_2OC_{14}H_9]\}$ (**3s**@*γ*-CD)

Na-**3s** (14 mg, 10 *μ*mol) was dissolved in 500 *μ*L of water at 40 °C. To this solution *γ*-CD (14 mg, 11 *μ*mol) in 600 *μ*L of acetonitrile/water (v/v = 1/1) solution was added. The resulting mixture was kept at room temperature for slow evaporation. Light-brown block crystals were obtained after one day. Yield: 14 mg of 54%. ^{1}H-NMR (400 MHz, D_2O): *δ* = 7.73 - 7.37 (m, 6H), 7.19 (s, 1H), 6.69 (br, 1H), 5.77 (br, 1H), 4.92 - 4.83 (m, 14H), 3.85 - 3.39 (m, 50H). ESI-TOF-MS (H_2O, neg. mode): *m/z* = 1964.68 ($\{Na•\mathbf{3s}@(\gamma\text{-CD})_2•H_2O\}^{2-}$, *calcd*. 1964.68), 1955.64 ($\{Na•\mathbf{3s}@(\gamma\text{-CD})_2\}^{2-}$, *calcd*. 1955.68), 1944.63 ($\{H•\mathbf{3s}@(\gamma\text{-CD})_2\}^{2-}$, *calcd*. 1944.69), 1728.55 ($\{\mathbf{3s}@(\gamma\text{-CD})_3\}^{3-}$, *calcd*. 1728.49), 1316.42 ($\{Na•\mathbf{3s}@\gamma\text{-CD}•H_2O\}^{2-}$, *calcd*. 1316.12), 1307.43 ($\{Na•\mathbf{3s}@\gamma\text{-CD})\}^{2-}$, *calcd*. 1307.11), 1296.10 ($\{\mathbf{3s}@(\gamma\text{-CD})_2\}^{3-}$, *calcd*. 1296.12), 1295.97 ($\{H•\mathbf{3s}@\gamma\text{-CD}\}^{2-}$, *calcd*. 1296.12). FT-IR (KBr, cm^{-1}): 3366 (s), 2926 (m), 2052 (m), 1634 (m), 1553 (w), 1418 (w), 1337 (w), 1263 (w), 1159 (m), 1030 (s), 929 (s), 663 (s), 581 (m), 447 (m). Elemental analysis (%) calcd for $C_{68}H_{101}AlMo_6O_{66}NNa_3$ with 6 H_2O solvent molecules: Na 2.49, Al 0.97, Mo 20.80, C 29.50, H 4.11, N 0.51; found: Na 2.32, Al 0.90, Mo 20.45, C 28.79, H 4.28, N 0.59.

Synthesis of 4@*γ*-CD (photodimerization of **3s**@*γ*-CD)

Single crystals of **3s**@*γ*-CD in mother liquid were immersed in Paratone oil and placed in a homemade photo-device. The device was filled with nitrogen gas and cooled to -10 °C before UV irradiation using LED lights (50 W, *λ* = 365 nm) with output power of 5-80 mW/cm^2. The crystal color changed to dark brown after irradiation (Figure S59).

Section 3. The ^{1}H-NMR, 2D NOESY ^{1}H NMR, Crystal Structures, ESI-TOF-MS, ITC, and TGA of all the hybrid (*pseudo*-)rotaxanes

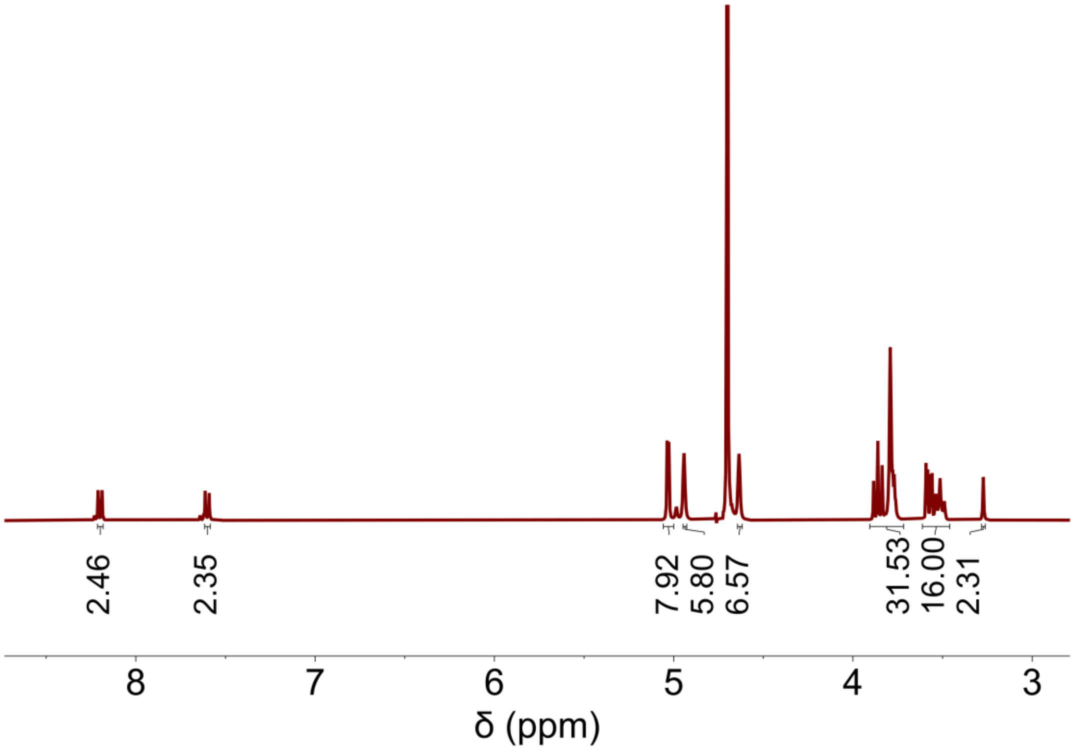

Figure S11. The ^{1}H-NMR spectrum of **1**@γ-CD in D_2O.

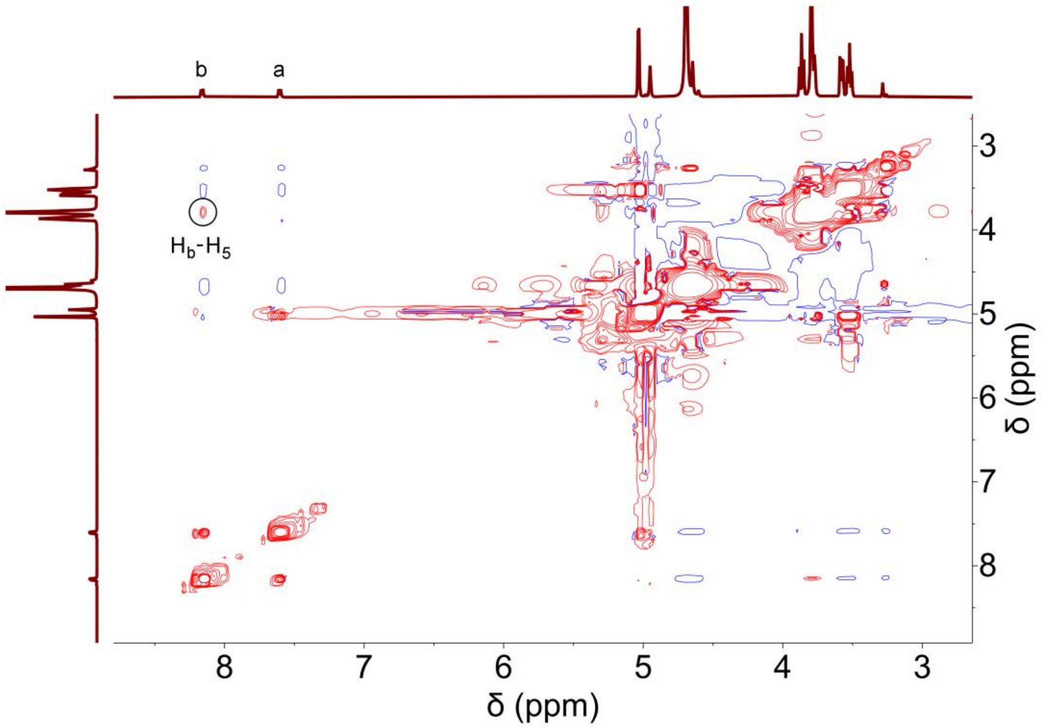

Figure S12. The 2D NOESY ^{1}H NMR spectrum of **1**@γ-CD in D_2O.

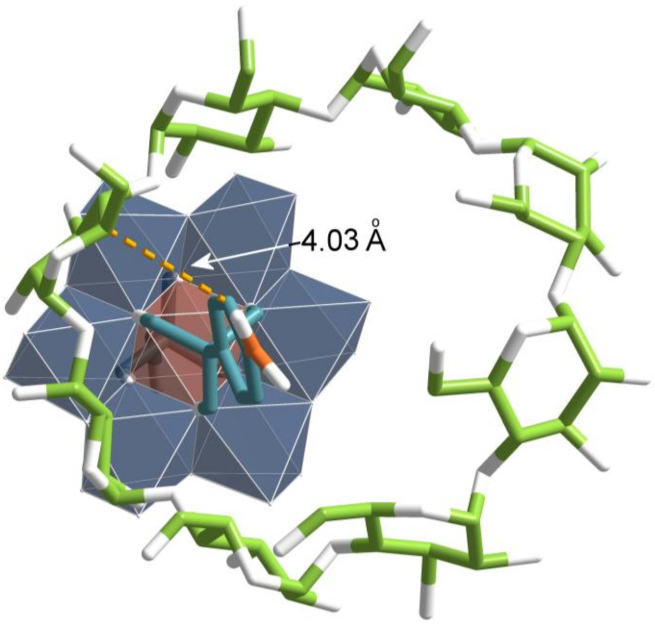

Figure S13. The structure of **1**@γ-CD showing the close distance between C_b of **1** and C_5 of γ-CD.

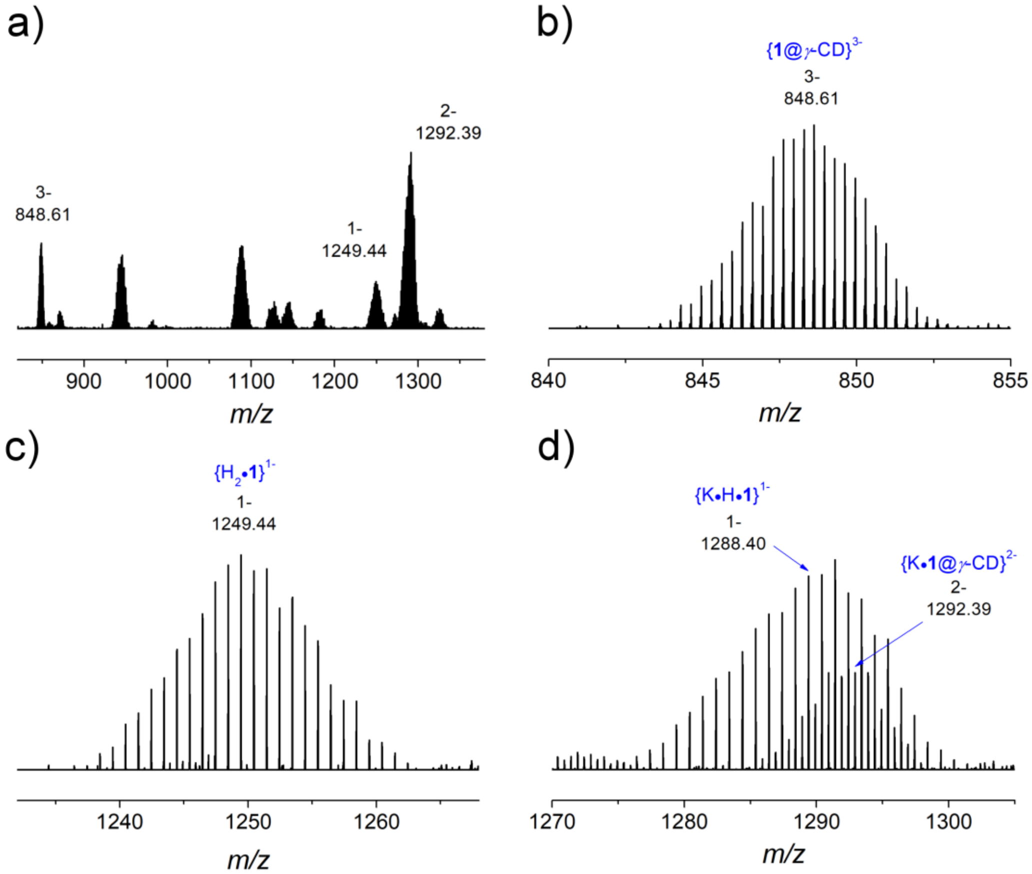

Figure S14. The ESI-TOF-MS spectra of **1**@γ-CD in water in negative mode.

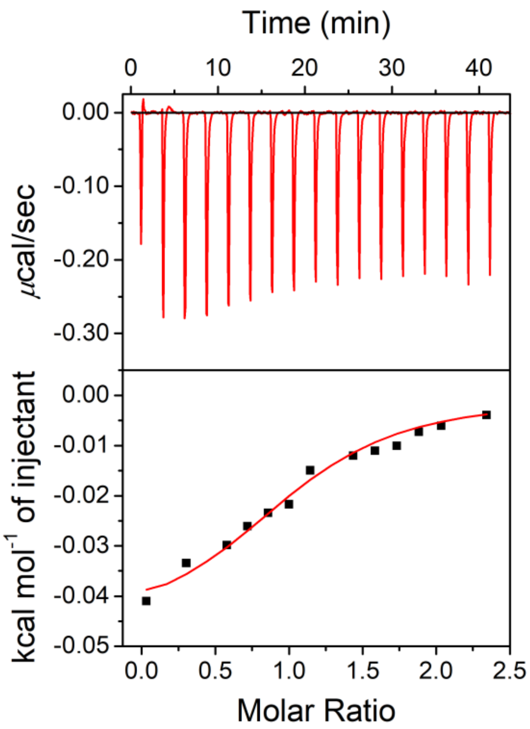

Figure S15. The ITC thermogram (top) and isotherm (bottom) of **1**@γ-CD. Dots and lines correspond to experimental and theoretical heat values, respectively.

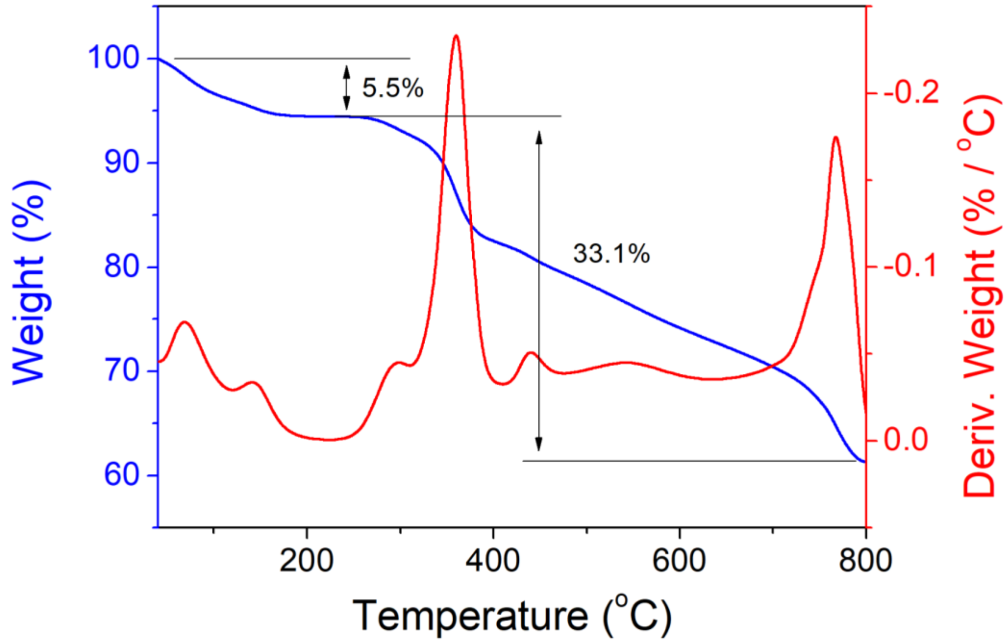

Figure S16. The TG-DTG curve of complex **1**@γ-CD. The first stage weight loss of 5.5% is due to the loss of 8 crystalline H_2O. The rest weight loss of 33.1% is due to the part loss of organic component.

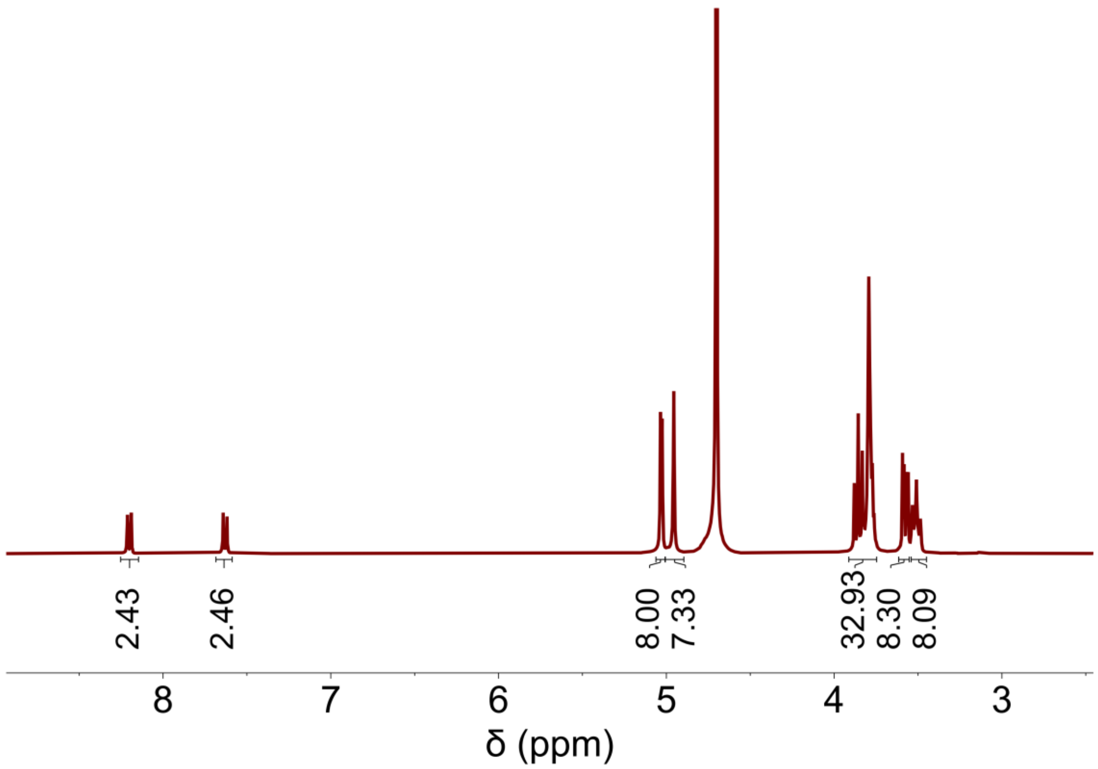

Figure S17. The ^{1}H-NMR spectrum of **1s**@γ-CD in D_2O.

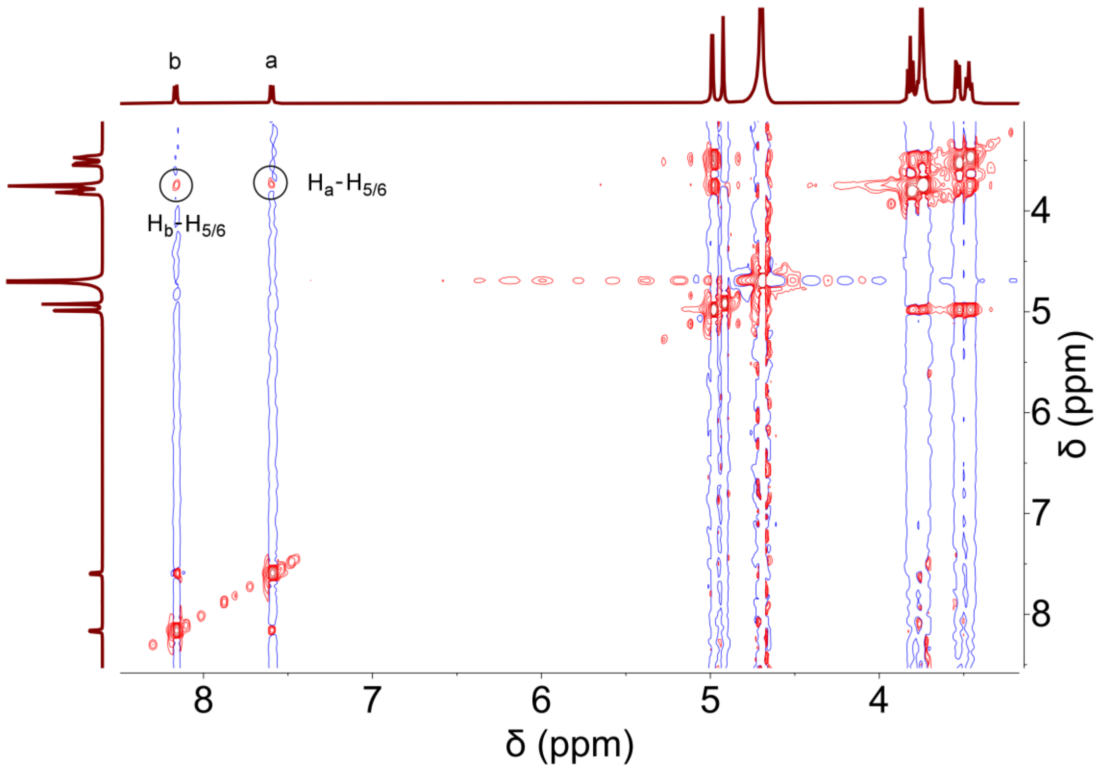

Figure S18. The 2D NOESY ^{1}H NMR spectrum of **1s**@γ-CD in D_2O.

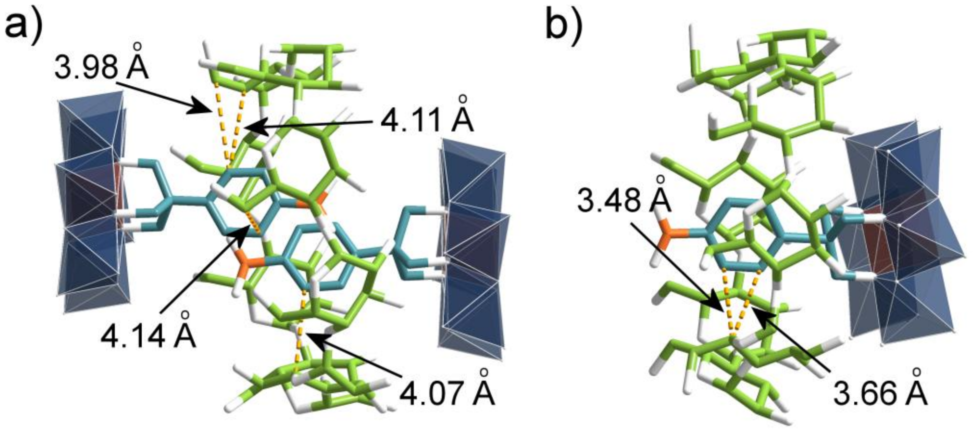

Figure S19. The crystal structure of **1s**@γ-CD showing the close interaction between H_a/H_b of **1s** and $H_{5/6}$ of γ-CD.

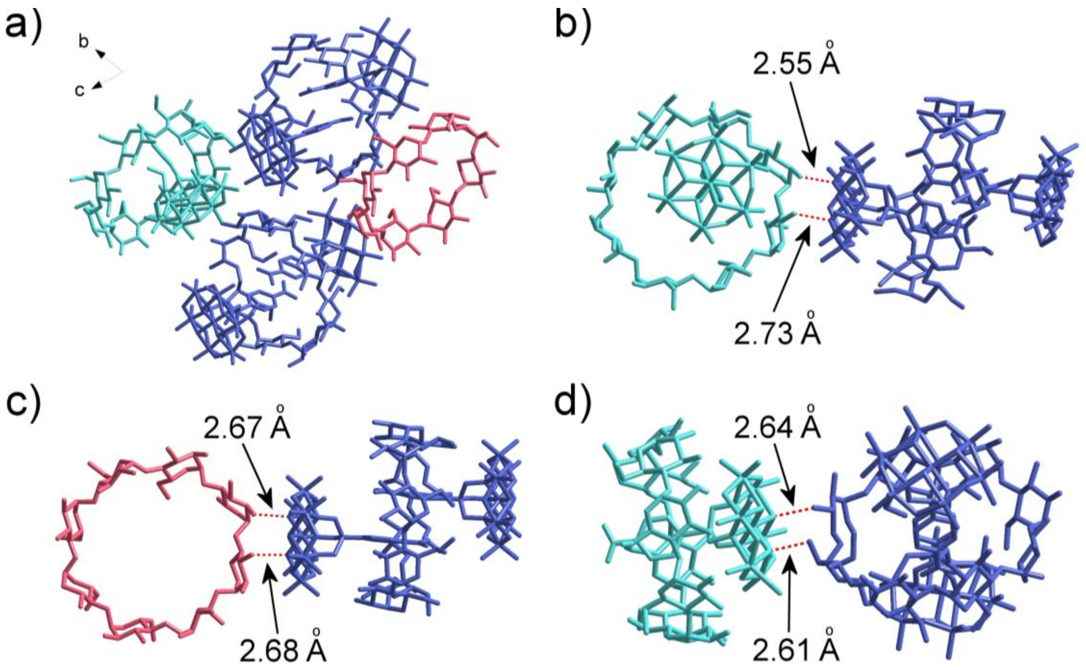

Figure S20. a) The asymmetric unit of **1s**@γ-CD viewing along the *a* axis, b) the intermolecular O•••O distances between hydroxy groups of γ-CD of the *pseudo*-[2]rotaxane and μ_3-O atoms of the *pseudo*-[3]rotaxane, c) the intermolecular O•••O distances between hydroxy groups of the free γ-CD and μ_3-O atoms of the *pseudo*-[3]rotaxane, and d) the intermolecular O•••O distances between hydroxy groups of γ-CD of the *pseudo*-[3]rotaxane and μ_3-O atoms of the *pseudo*-[2]rotaxane. The free γ-CDs are depicted in pink, the *pseudo*-[2]rotaxanes are in cyan, and the *pseudo*-[3]rotaxanes are in blue. The O•••O distances are depicted in red dotted lines.

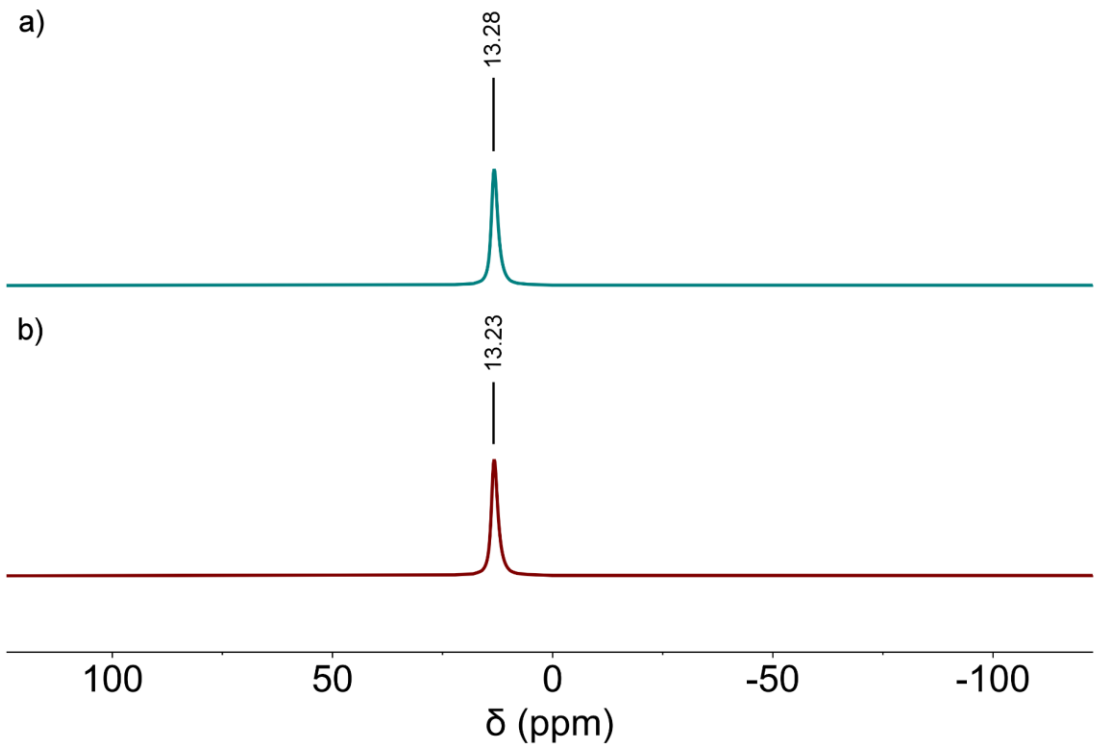

Figure S21. The ^{27}Al NMR of a) **1**@γ-CD and b) Na-**1** in D_2O.

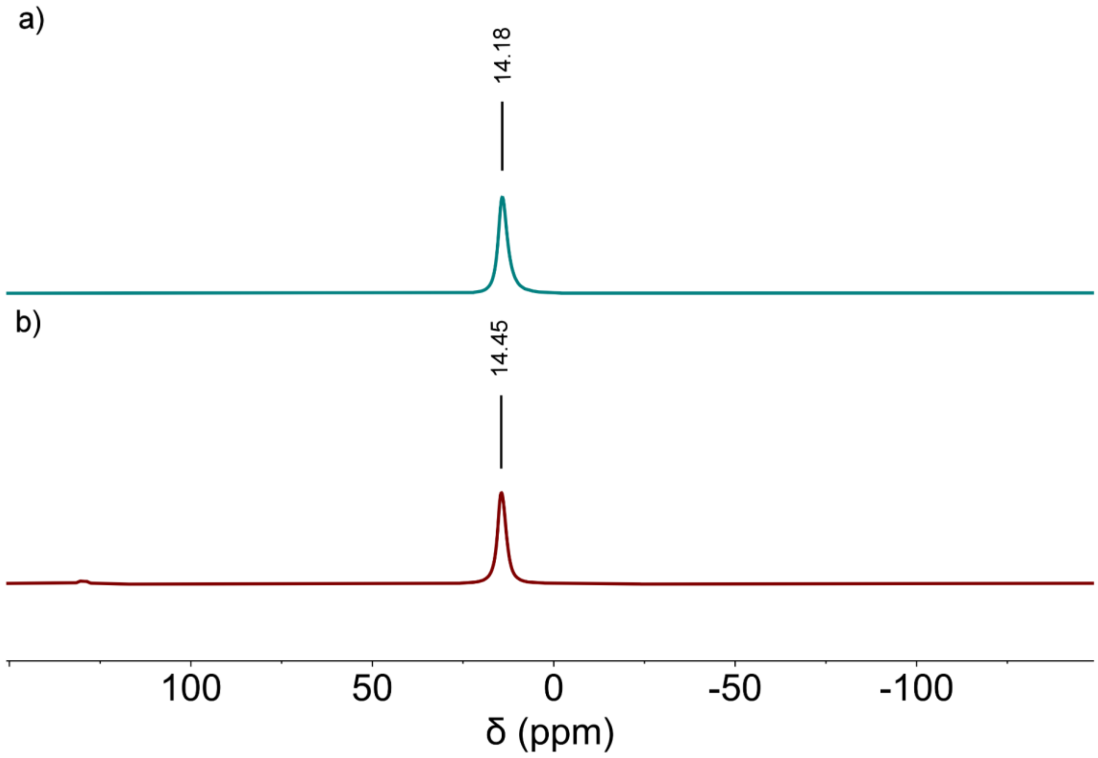

Figure S22. The ^{27}Al NMR of a) **1s**@γ-CD and b) Na-**1s** in D_2O.

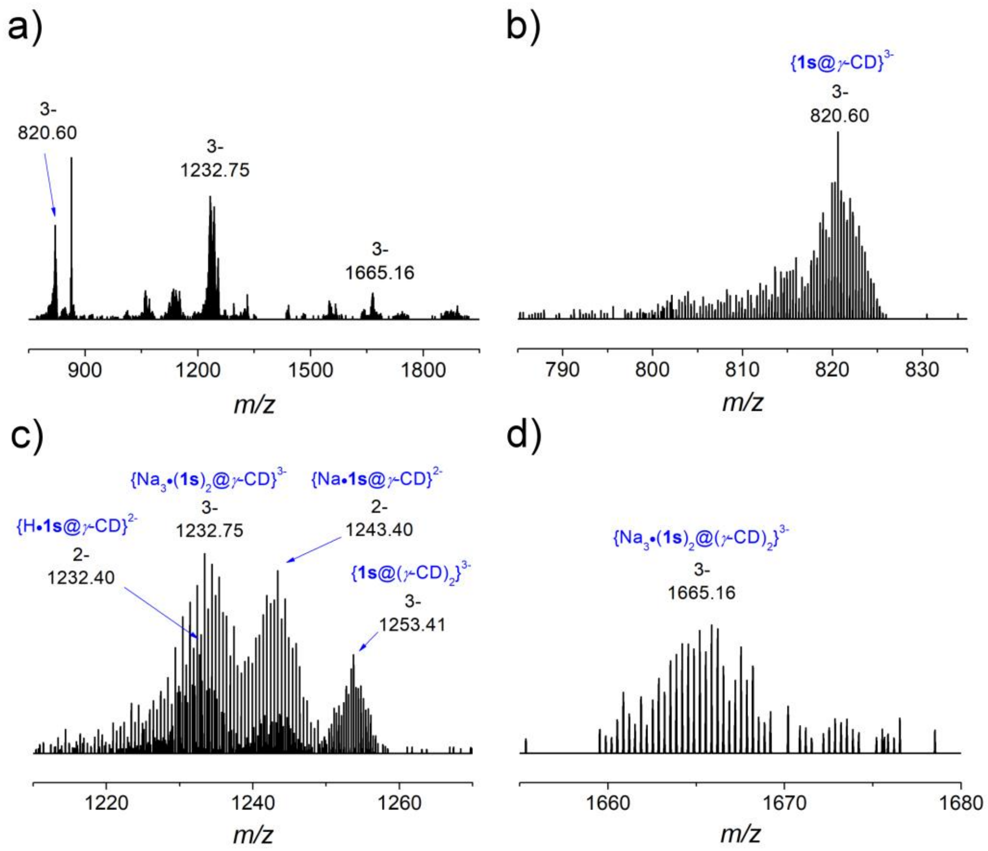

Figure S23. The ESI-TOF-MS spectra of **1s**@γ-CD in water in negative mode.

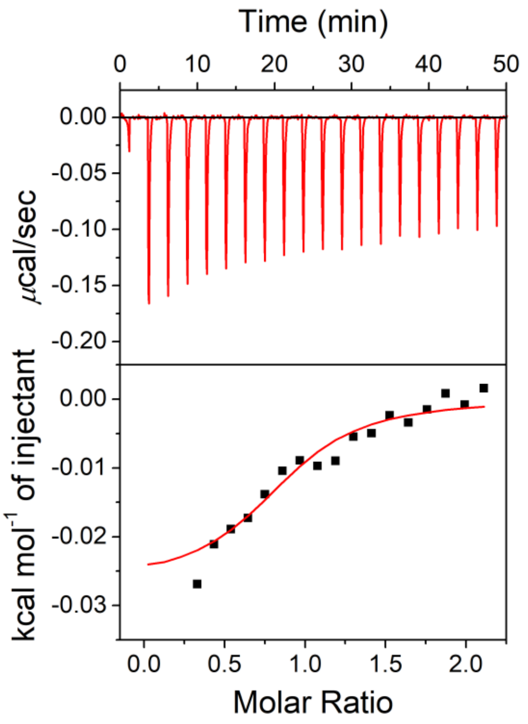

Figure S24. The ITC thermogram (top) and isotherm (bottom) of **1s**@γ-CD. Dots and lines correspond to experimental and theoretical heat values, respectively.

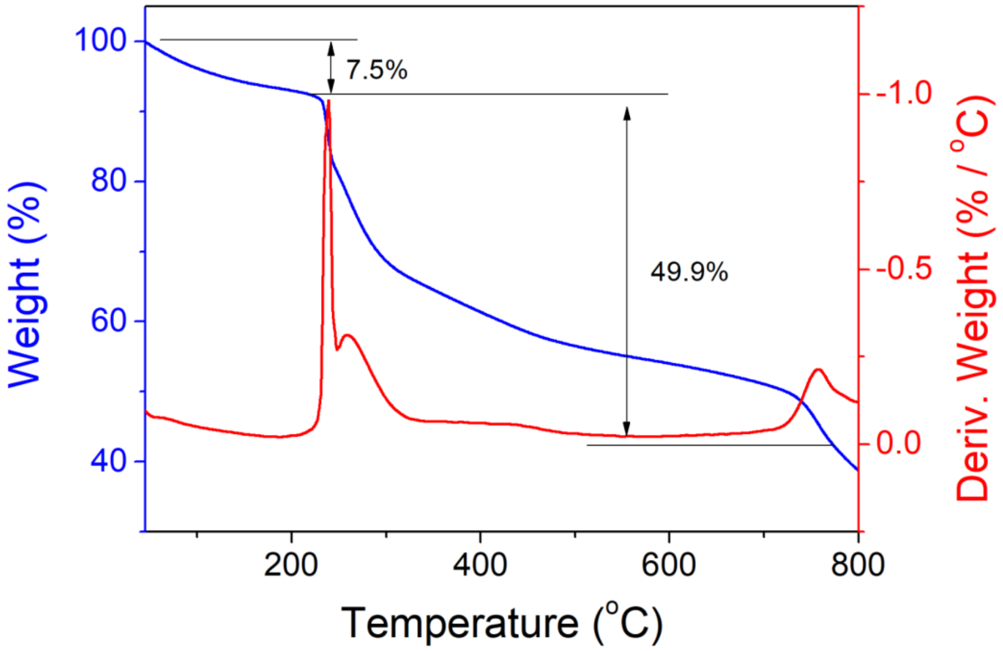

Figure S25. The TG-DTG curve of complex **1s**@γ-CD. The first stage weight loss of 7.5% is due to the loss of 50 crystalline H_2O. The rest weight loss of 49.9% (calcd 49.6%) is due to the loss of organic component.

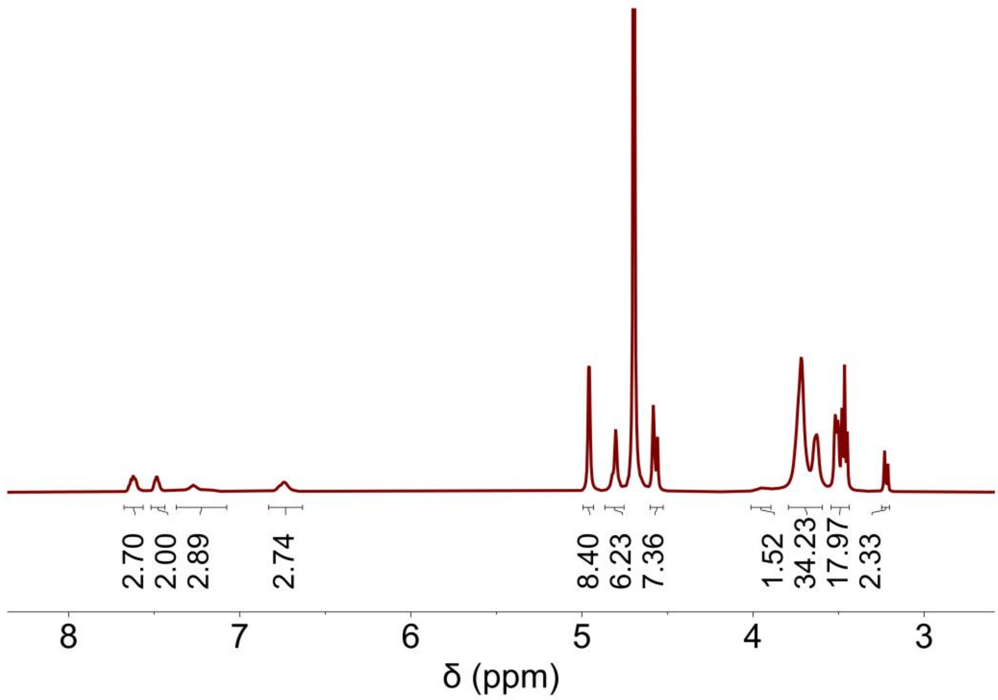

Figure S26. The ^{1}H-NMR spectrum of **2**@γ-CD in D_2O.

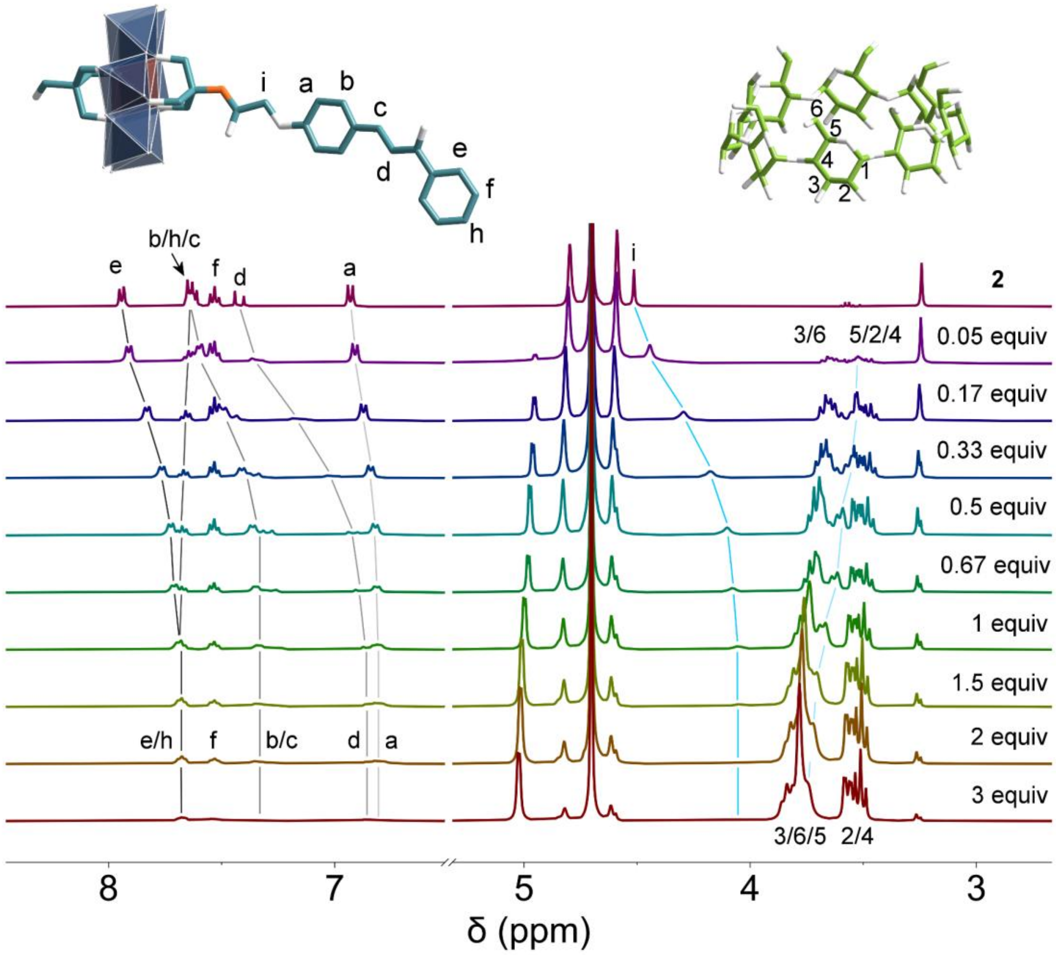

Figure S27. The ^{1}H-NMR titration of the supramolecular assembly of γ-CD and **2** upon successive addition of γ-CD into **2** in D_2O (c = 6 mmol/L). The equivalent amounts of γ-CD to **2** are labelled.

Table S1. Binding constants of γ-CD with the asymmetric Anderson hybrids and associated thermodynamic parameters of ITC.

	N (Sites)	*K* (M^{-1})	Δ_rH_m (cal/mol)	Δ_rS_m (cal/mol/°C)	Δ_rG_m (cal/mol)
Na-**1**	1.04 ± 0.0809	$3.09 \times 10^3 \pm 1.04 \times 10^3$	-46.95 ± 5.00	15.8	-441.95 ± 5.00
Na-**1s**	0.864 ± 0.0800	$7.36 \times 10^3 \pm 3.55 \times 10^3$	-26.65 ± 3.895	17.6	-466.65 ± 3.895
Na-**2**	0.975 ± 0.222	$1.38 \times 10^3 \pm 0.422 \times 10^3$	-2155 ± 718.3	7.13	-2333.25 ± 718.3
Na-**2s**	0.818 ± 0.0524	$1.57 \times 10^4 \pm 5.94 \times 10^3$	-525.9 ± 53.61	17.5	-963.4 ± 53.61
Na-**3**	0.908 ± 0.0626	$1.15 \times 10^4 \pm 1.96 \times 10^3$	-1204 ± 110.3	14.5	-1566.5 ± 110.3
Na-**3s**	0.971 ± 0.0750	$1.04 \times 10^4 \pm 2.06 \times 10^3$	-1536 ± 214.2	13.3	-1868.5 ± 214.2

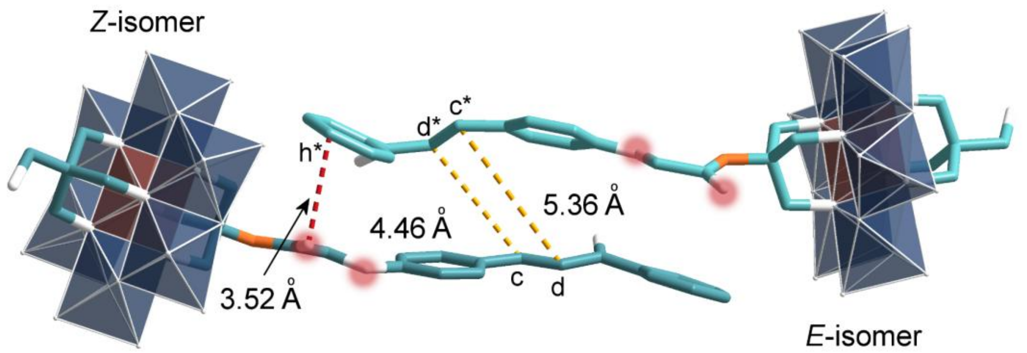

Figure S28. The spatial distances of the *α*, *β*-unsaturated carbonyl bonds of the *Z*- and *E*-isomers in **2**@*γ*-CD.

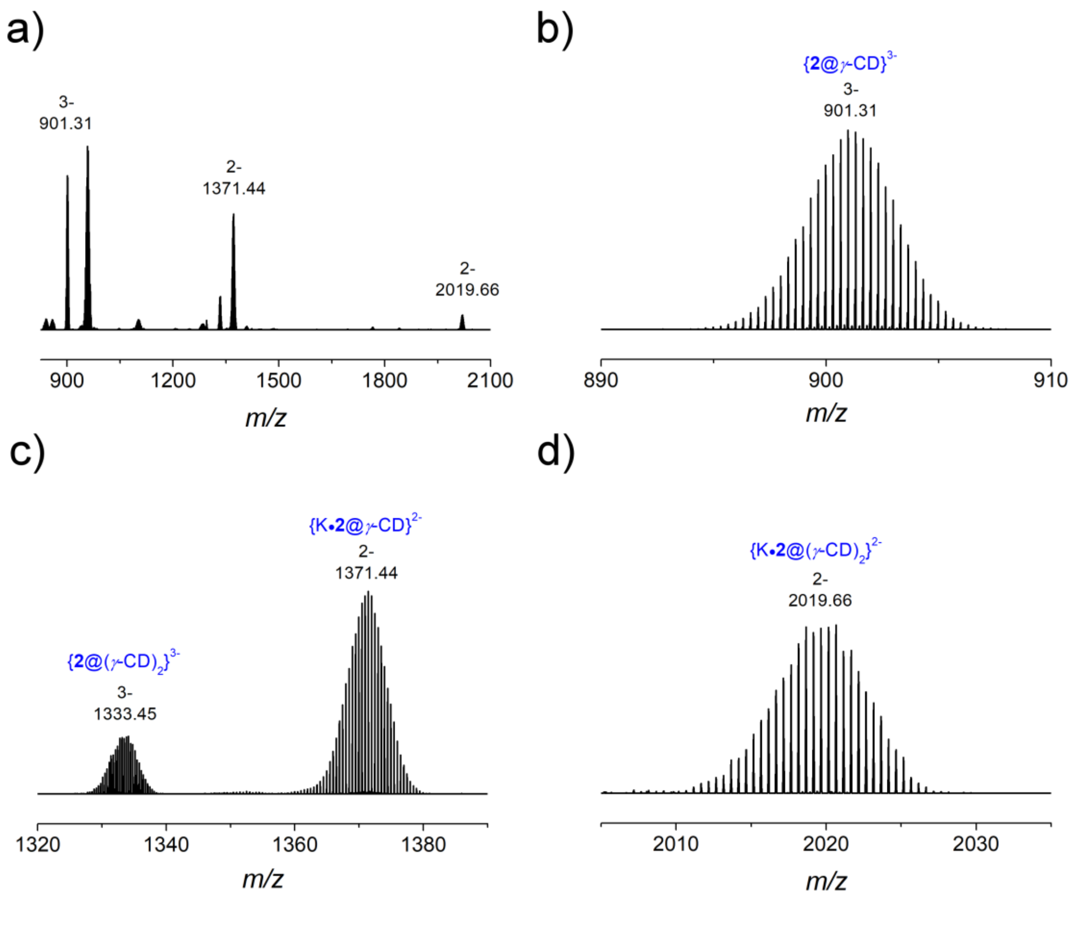

Figure S29. The ESI-TOF-MS spectra of **2**@*γ*-CD in water in negative mode.

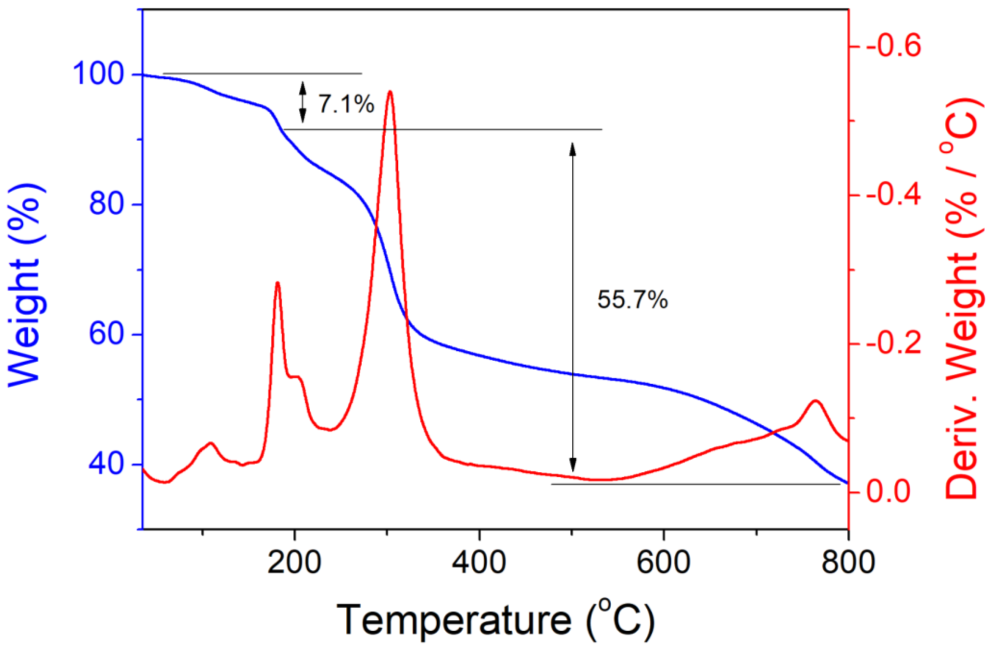

Figure S30. The TG-DTG curve of complex **2**@γ-CD. The first stage weight loss of 7.1% is due to the loss of 12 crystalline H_2O. The rest weight loss of 55.7% (calcd 56.5%) is due to the loss of organic component.

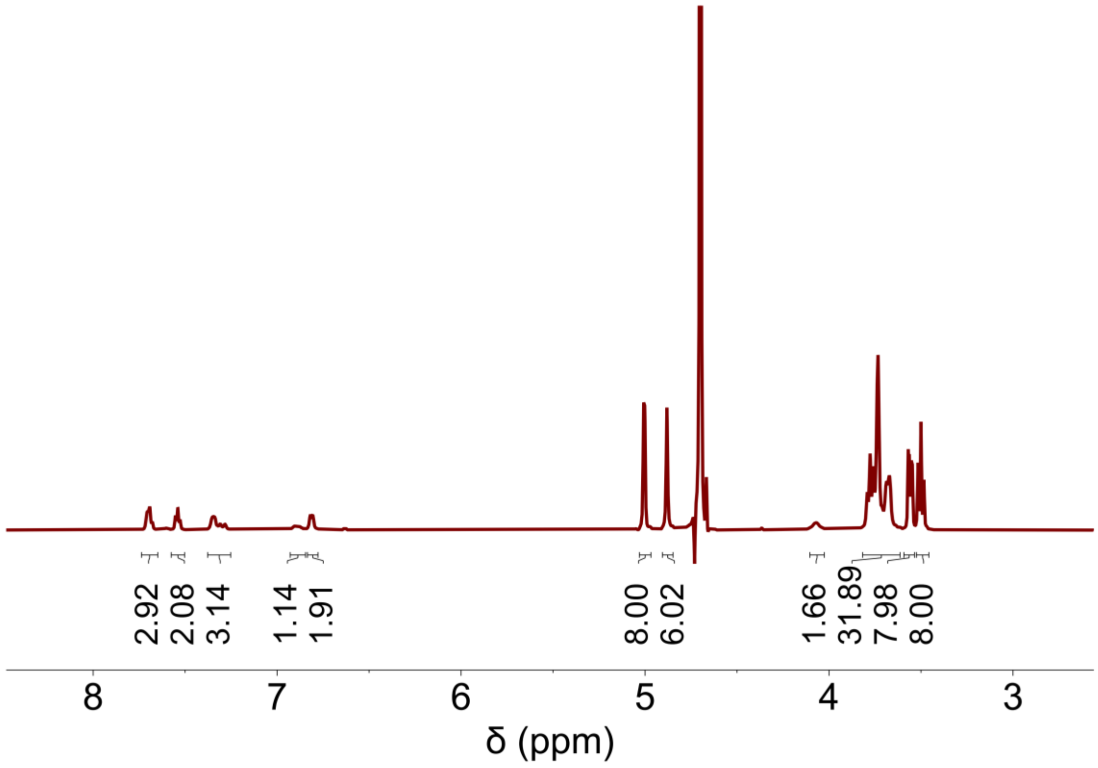

Figure S31. The ^{1}H-NMR spectrum of **2s**@γ-CD in D_2O.

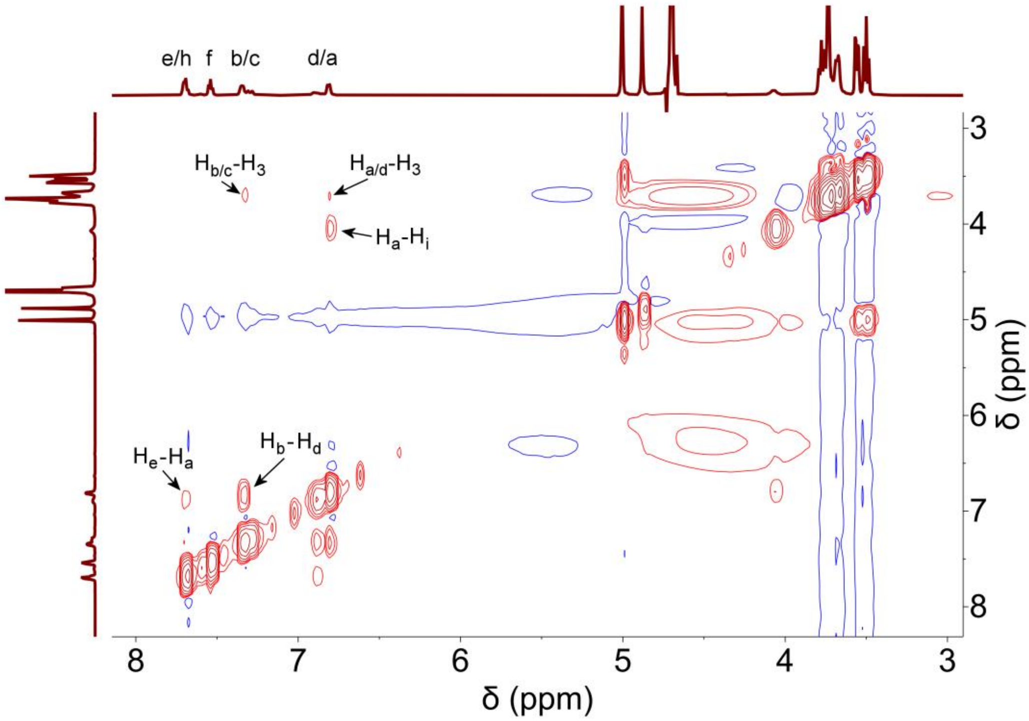

Figure S32. The 2D NOESY ^{1}H NMR spectrum of **2s**@γ-CD in D_2O.

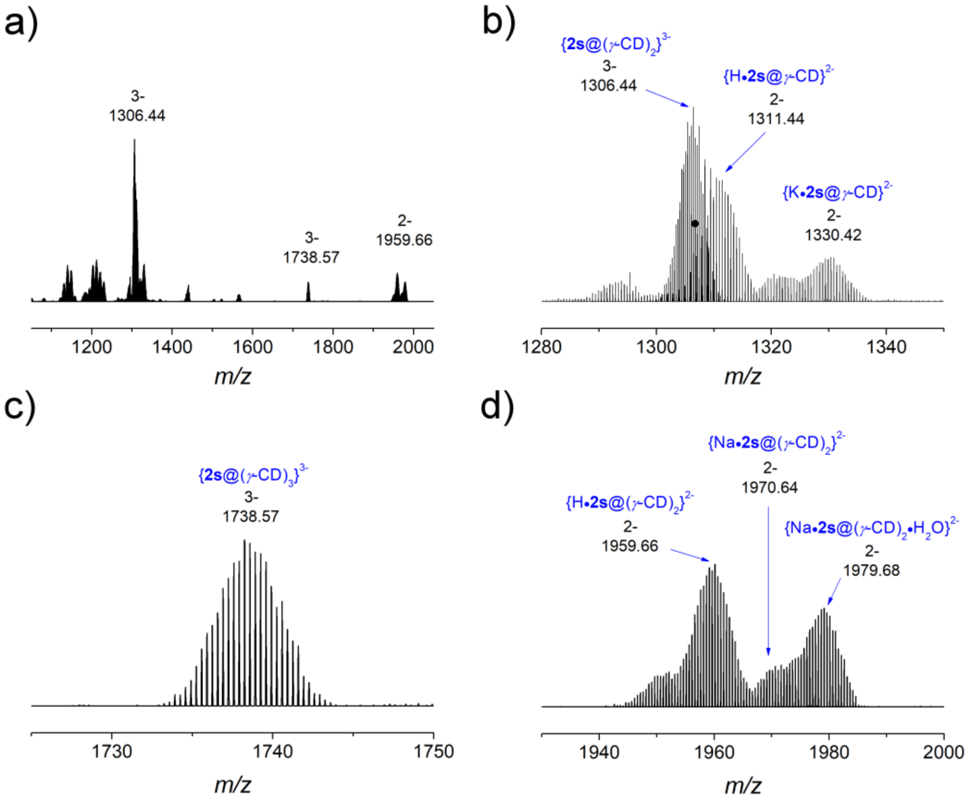

Figure S33. The ESI-TOF-MS spectra of **2s**@γ-CD in water in negative mode. The presence of sodium ions is due to the calibration of the instrument using 5 mM sodium iodide solution in 90:10 2-propanol: water.

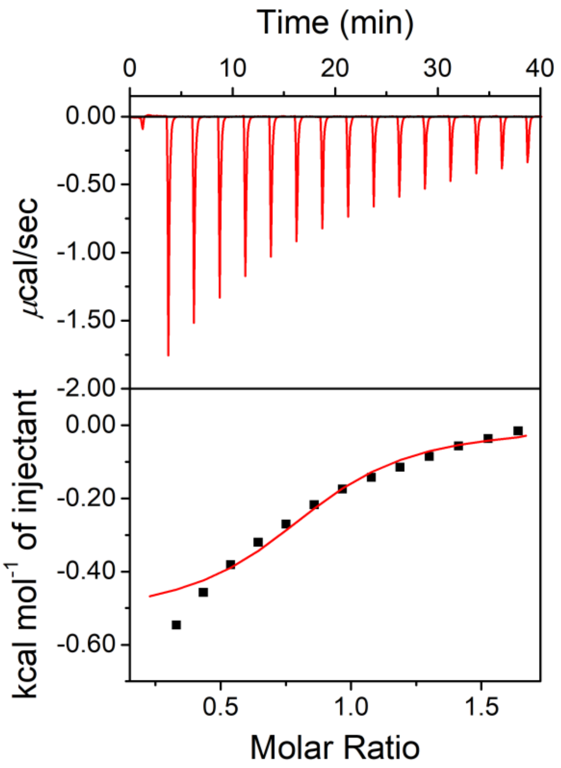

Figure S34. The ITC thermogram (top) and isotherm (bottom) of **2s**@γ-CD. Dots and lines correspond to experimental and theoretical heat values, respectively.

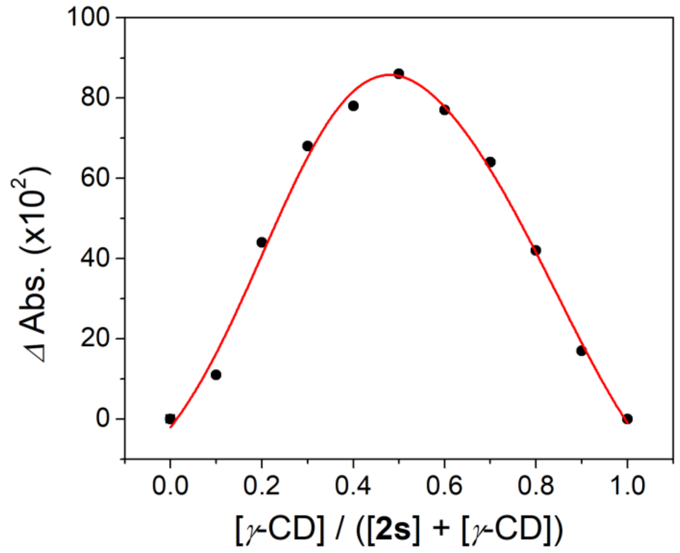

Figure S35. The Job plot of the UV-Vis absorbance changes at 340 nm showing the molar ratio of γ-CD and **2s** upon complexation.

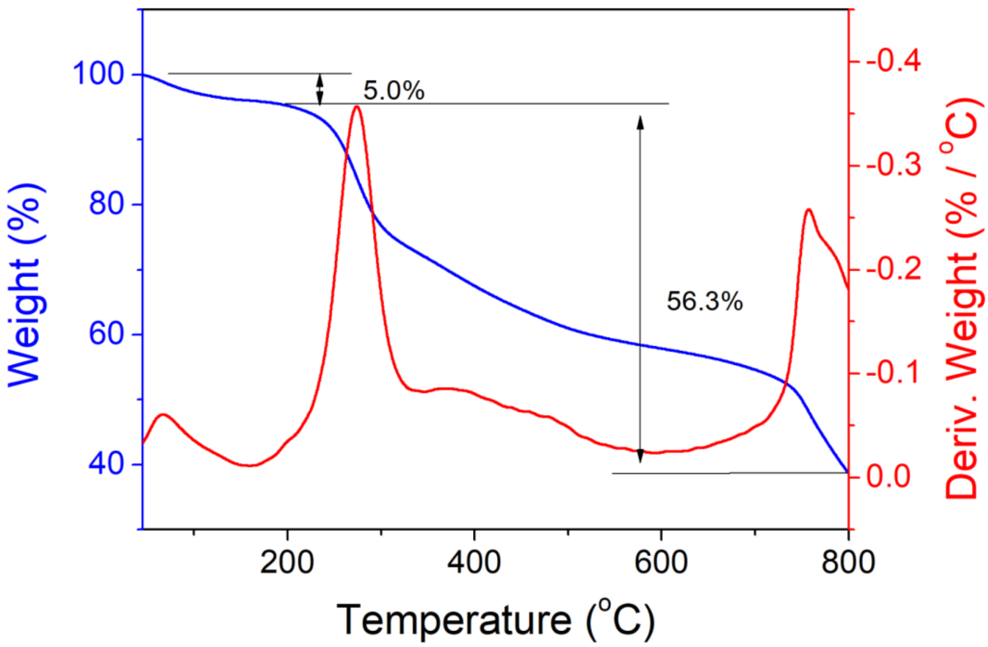

Figure S36. The TG-DTG curve of complex **2s**@γ-CD. The first stage weight loss of 5.0% is due to the loss of 8 crystalline H_2O. The rest weight loss of 56.3% (calcd 56.7%) is due to the loss of organic component.

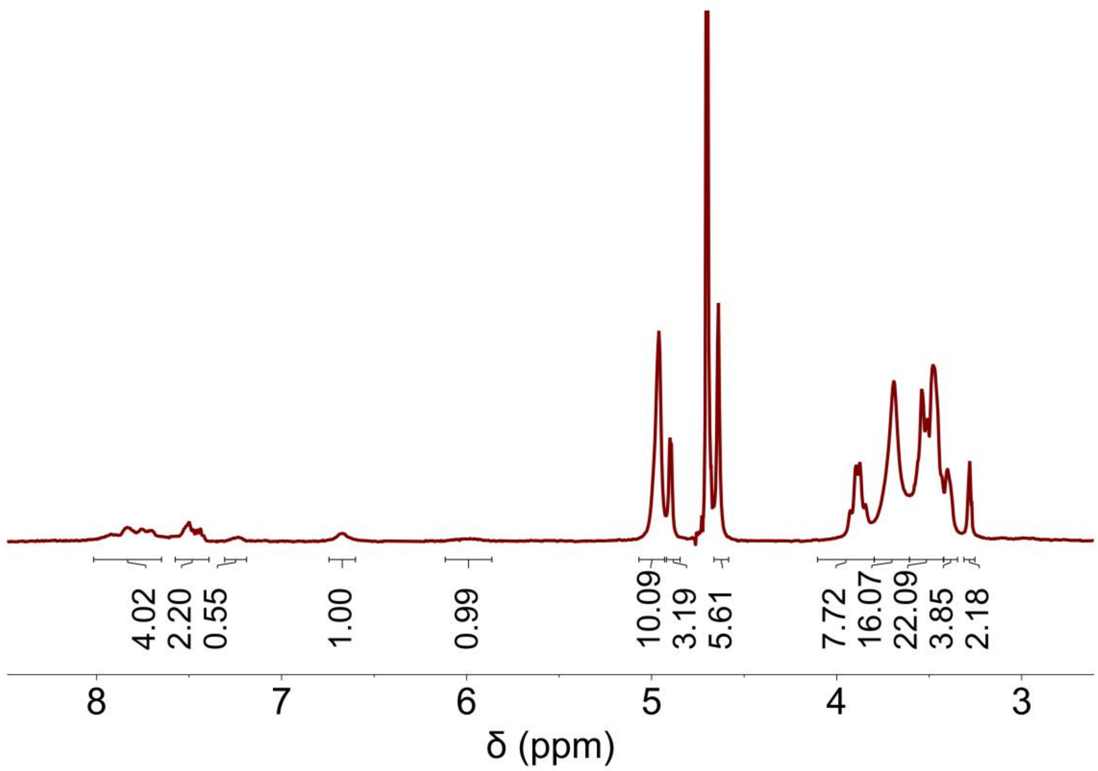

Figure S37. The ^{1}H-NMR spectrum of **3**@γ-CD in D_2O.

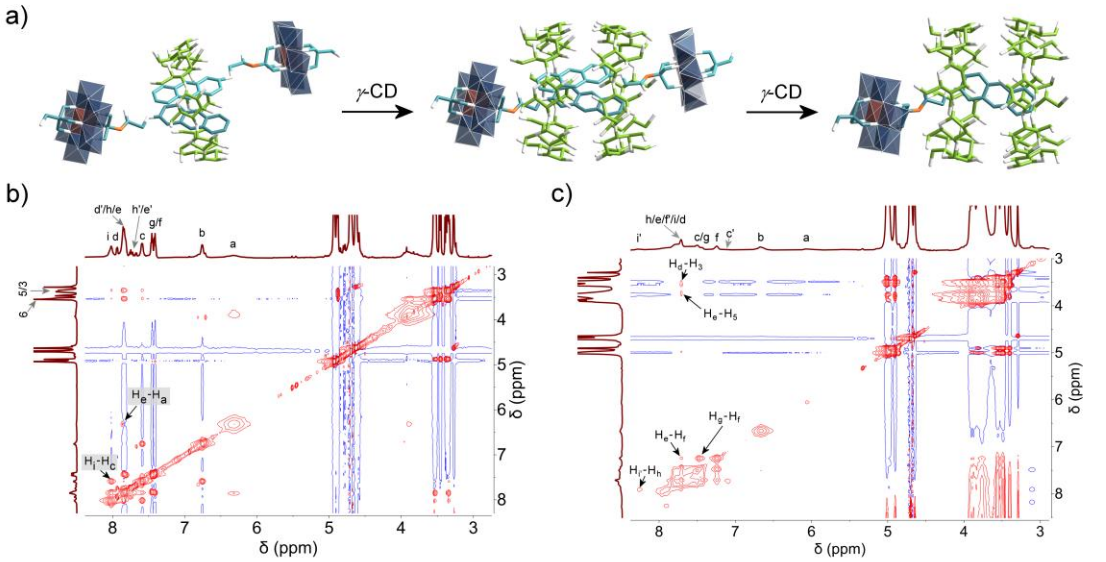

Figure S38. a) The schematic representation of the complexation process between **3** and *γ*-CD with the successive addition of *γ*-CD into **3**, b) the 2D NOESY ^{1}H NMR spectrum of the mixture of **3** and *γ*-CD at 3:1 molar ratio, and c) the 2D NOESY ^{1}H NMR spectrum of the mixture of **3** and *γ*-CD at 1:3 molar ratio.

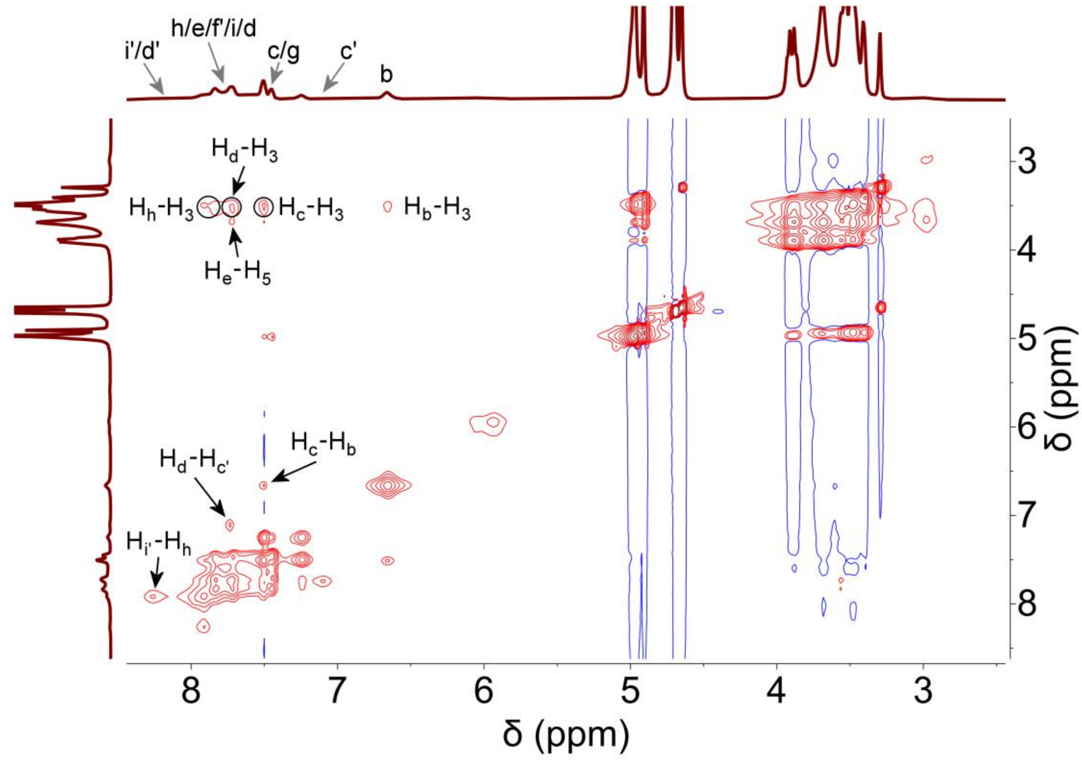

Figure S39. The 2D NOESY ^{1}H NMR spectrum of **3**@*γ*-CD in D_2O.

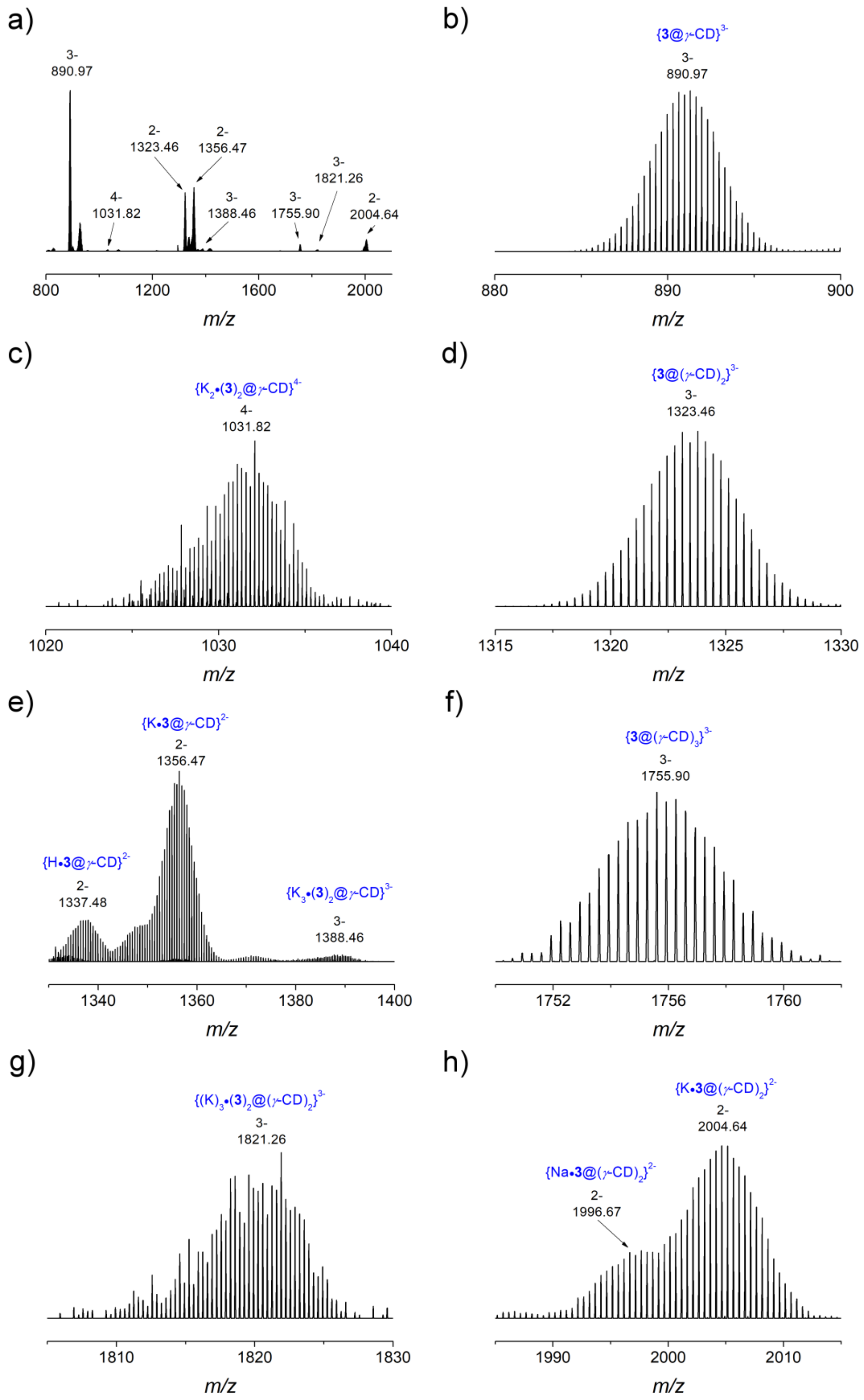

Figure S40. The ESI-TOF-MS spectra of **3**@γ-CD in water in negative mode.

Table S2 The observed and calculated ESI-TOF-MS peaks of **3**@γ-CD.

Assignment	z	code	*m*/*z* (obv.)	*m*/*z* (calcd.)
$\{AlMo_6O_{18}[(OCH_2)_3CCH_2OH][(OCH_2)_3CNHCOCH_2OC_{14}H_9]\}(C_{48}H_{80}O_{40})$	3-	$\{$**3**@γ-CD$\}^{3-}$	890.97	891.11
$K_2\{AlMo_6O_{18}[(OCH_2)_3CCH_2OH][(OCH_2)_3CNHCOCH_2OC_{14}H_9]\}_2(C_{48}H_{80}O_{40})$	4-	$\{K_2$•(**3**)$_2$@γ-CD$\}^{4-}$	1031.82	1031.94
$\{AlMo_6O_{18}[(OCH_2)_3CCH_2OH][(OCH_2)_3CNHCOCH_2OC_{14}H_9]\}(C_{48}H_{80}O_{40})_2$	3-	$\{$**3**@(γ-CD)$_2\}^{3-}$	1323.46	1323.49
$H\{AlMo_6O_{18}[(OCH_2)_3CCH_2OH][(OCH_2)_3CNHCOCH_2OC_{14}H_9]\}(C_{48}H_{80}O_{40})$	2-	$\{H$•**3**@γ-CD$\}^{2-}$	1337.48	1337.46
$K\{AlMo_6O_{18}[(OCH_2)_3CCH_2OH][(OCH_2)_3CNHCOCH_2OC_{14}H_9]\}(C_{48}H_{80}O_{40})$	2-	$\{K$•**3**@γ-CD$\}^{2-}$	1356.47	1356.22
$K_3\{AlMo_6O_{18}[(OCH_2)_3CCH_2OH][(OCH_2)_3CNHCOCH_2OC_{14}H_9]\}_2(C_{48}H_{80}O_{40})$	3-	$\{K_3$•(**3**)$_2$@γ-CD$\}^{3-}$	1388.46	1388.95
$\{AlMo_6O_{18}[(OCH_2)_3CCH_2OH][(OCH_2)_3CNHCOCH_2OC_{14}H_9]\}(C_{48}H_{80}O_{40})_3$	3-	$\{$**3**@(γ-CD)$_3\}^{3-}$	1755.90	1755.86
$K_3\{AlMo_6O_{18}[(OCH_2)_3CCH_2OH][(OCH_2)_3CNHCOCH_2OC_{14}H_9]\}_2(C_{48}H_{80}O_{40})_2$	3-	$\{K_3$•(**3**)$_2$@(γ-CD)$_2\}^{3-}$	1821.26	1821.32
$Na\{AlMo_6O_{18}[(OCH_2)_3CCH_2OH][(OCH_2)_3CNHCOCH_2OC_{14}H_9]\}(C_{48}H_{80}O_{40})_2$	2-	$\{Na$•**3**@(γ-CD)$_2\}^{2-}$	1996.67	1996.73
$K\{AlMo_6O_{18}[(OCH_2)_3CCH_2OH][(OCH_2)_3CNHCOCH_2OC_{14}H_9]\}(C_{48}H_{80}O_{40})_2$	2-	$\{K$•**3**@(γ-CD)$_2\}^{2-}$	2004.64	2004.78

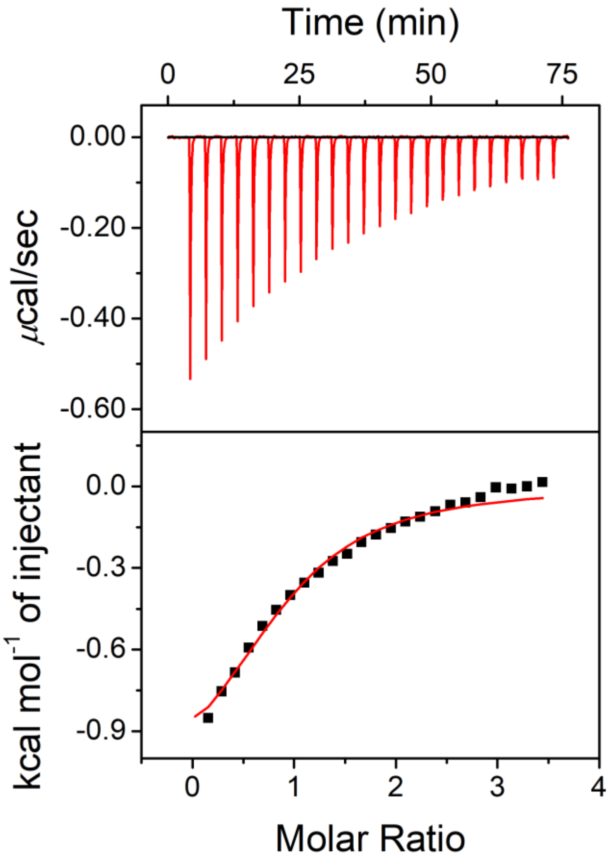

Figure S41. The ITC thermogram (top) and isotherm (bottom) of **3**@γ-CD. Dots and lines correspond to experimental and theoretical heat values, respectively.

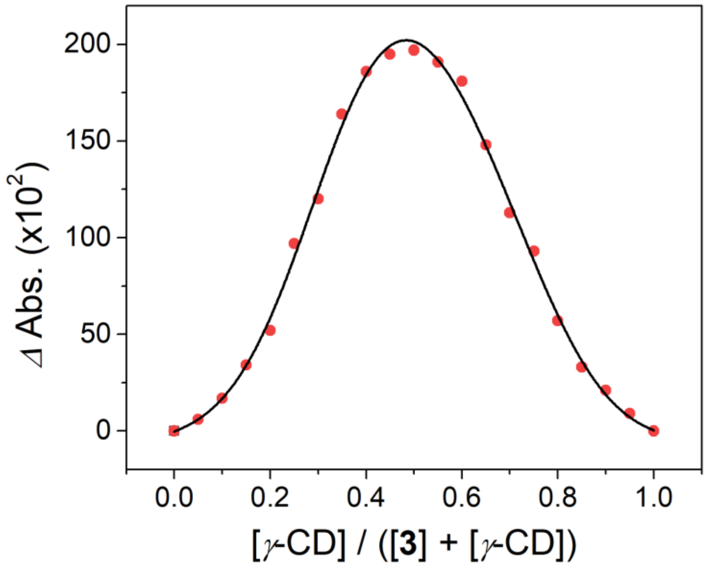

Figure S42. The Job plot of the UV-Vis absorbance changes at 255 nm showing the molar ratio of γ-CD and **3** upon complexation.

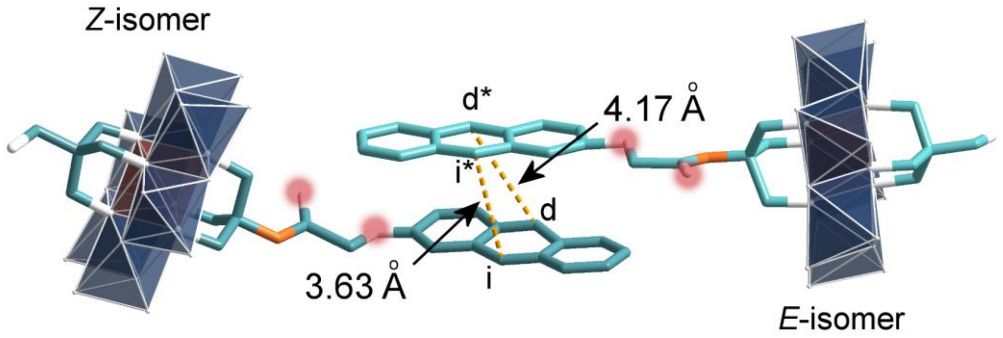

Figure S43. The spatial distances at the 9 (C_d-C_{d*}) and 10 (C_i-C_{i*}) positions of the *Z*- and *E*-isomers in **3**@γ-CD.

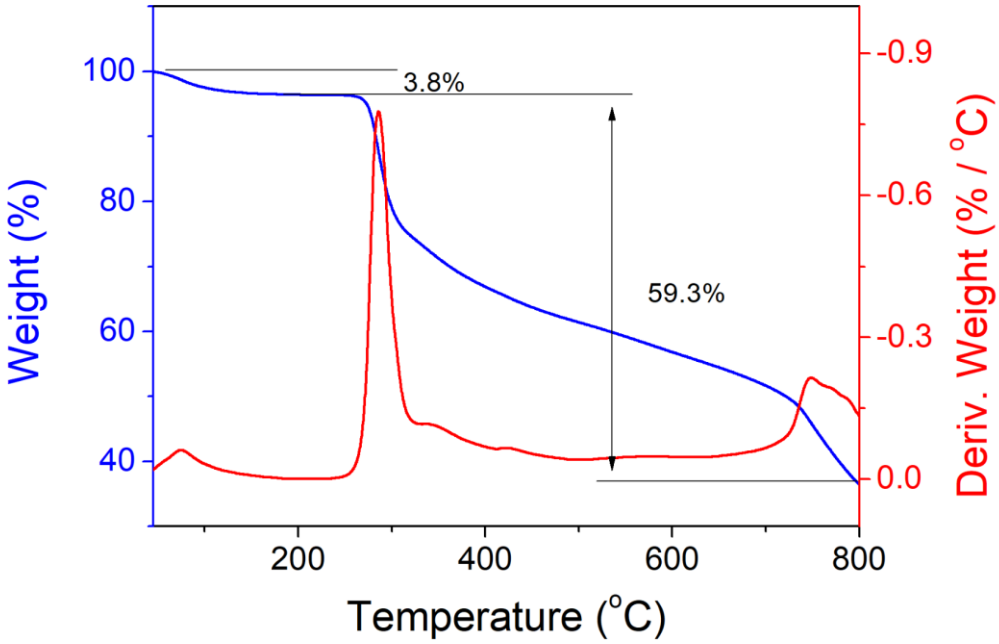

Figure S44. The TG-DTG curve of complex **3**@γ-CD. The first stage weight loss of 3.8% is due to the loss of 6 crystalline H_2O. The rest weight loss of 59.3% (calcd 58.3%) is due to the loss of organic component.

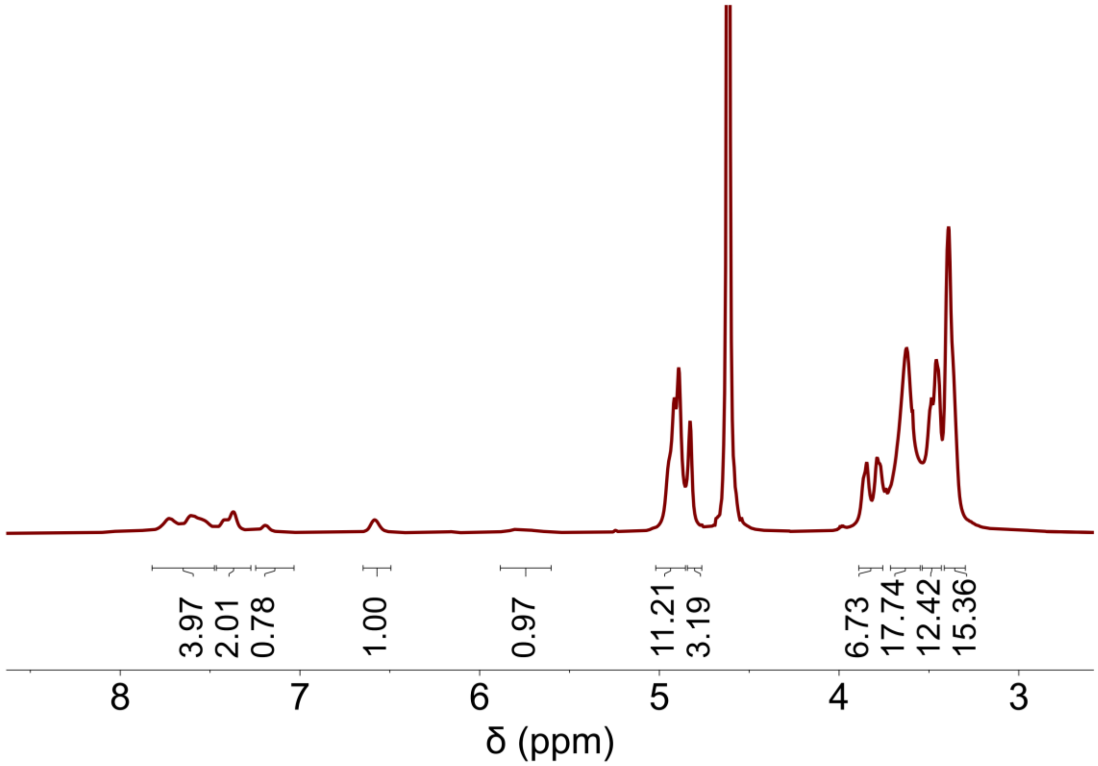

Figure S45. The ^{1}H-NMR spectrum of **3s**@γ-CD in D_2O.

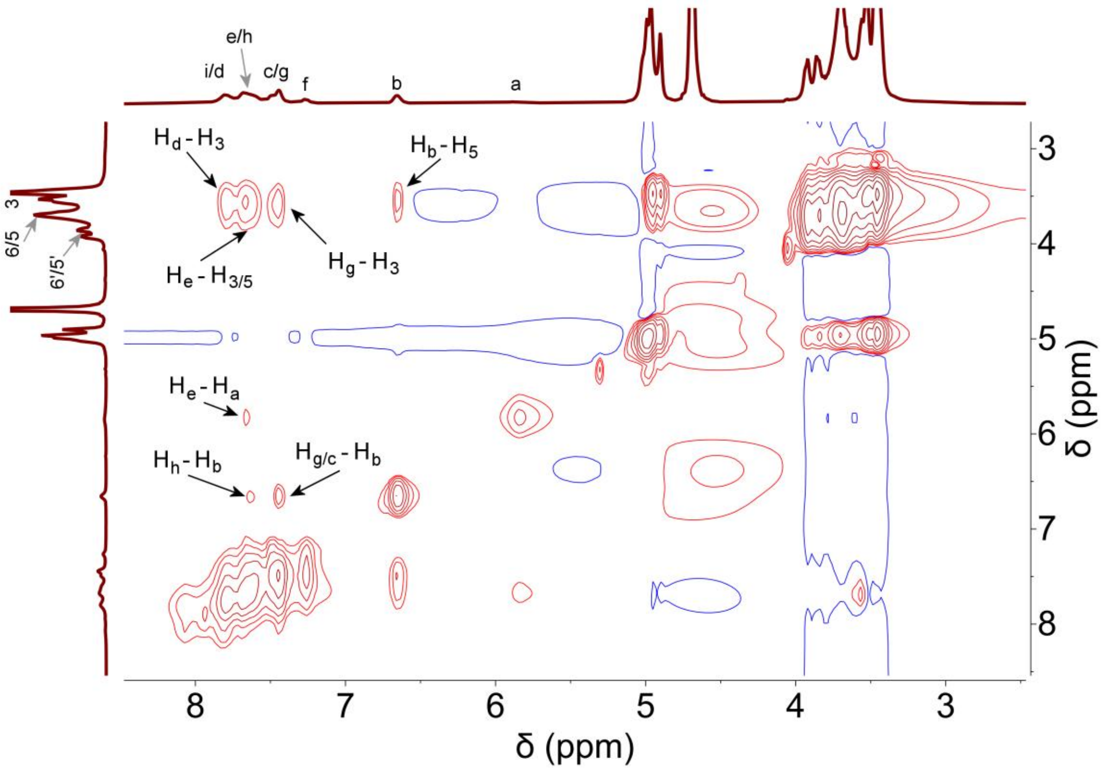

Figure S46. The 2D NOESY ^{1}H NMR spectrum of **3s**@*γ*-CD in D_2O.

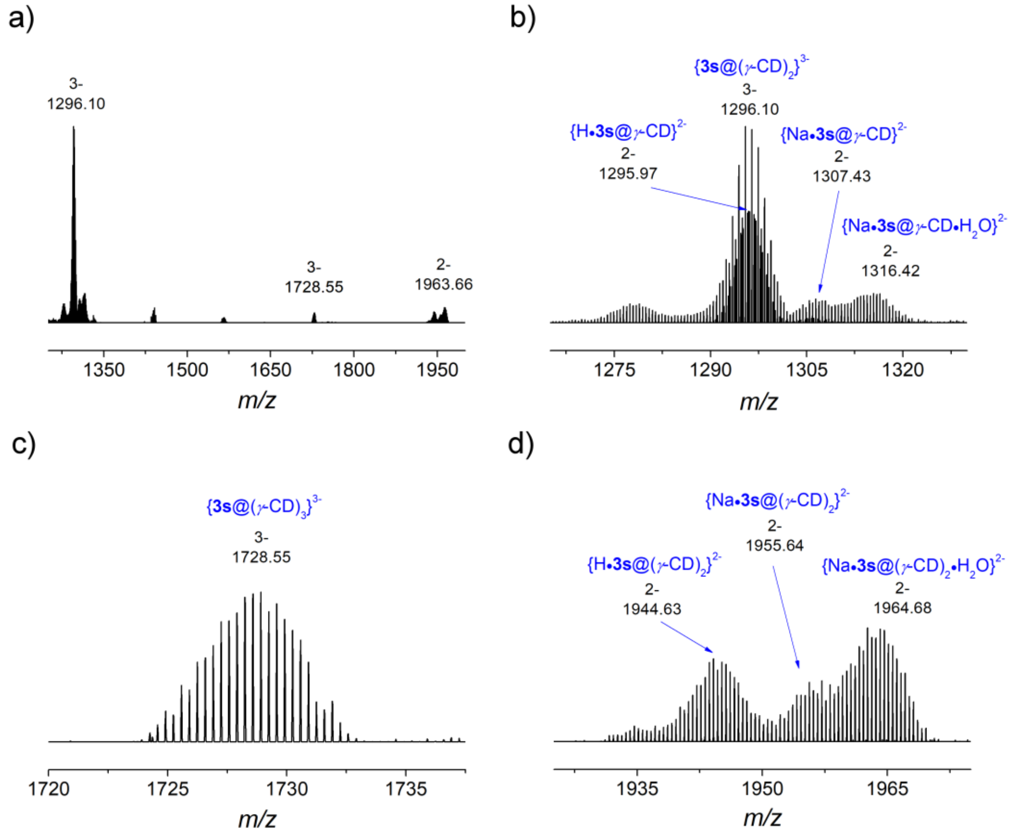

Figure S47. The ESI-TOF-MS spectra of **3s**@*γ*-CD in water in negative mode.

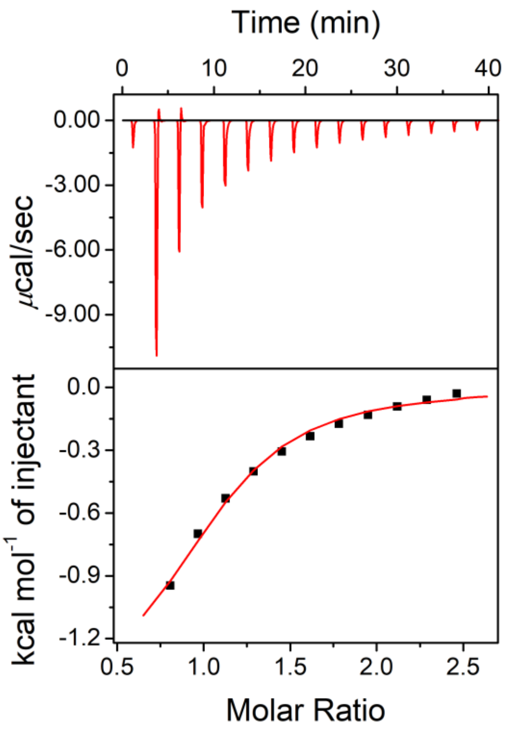

Figure S48. The ITC thermogram (top) and isotherm (bottom) of **3s**@γ-CD. Dots and lines correspond to experimental and theoretical heat values, respectively.

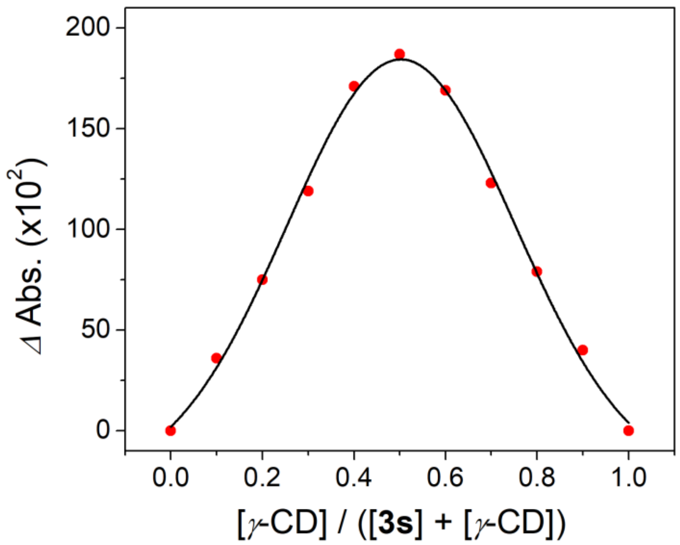

Figure S49. The Job plot of the UV-Vis absorbance changes at 255 nm showing the molar ratio of γ-CD and **3s** upon complexation.

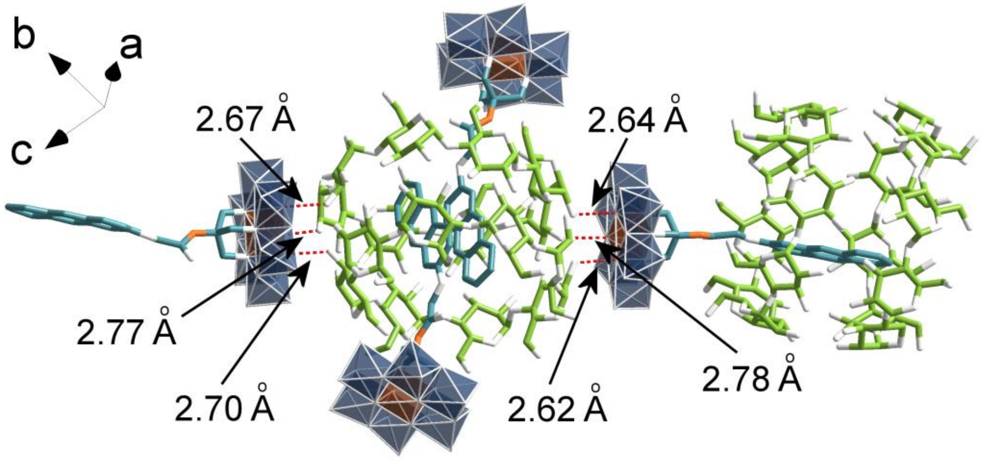

Figure S50. The close intermolecular O•••O distances (in red dotted lines) between the hydroxy groups of *γ*-CDs and the *μ*3-O atoms of the single-sided Anderson hybrids **3s** in complex **3s**@*γ*-CD.

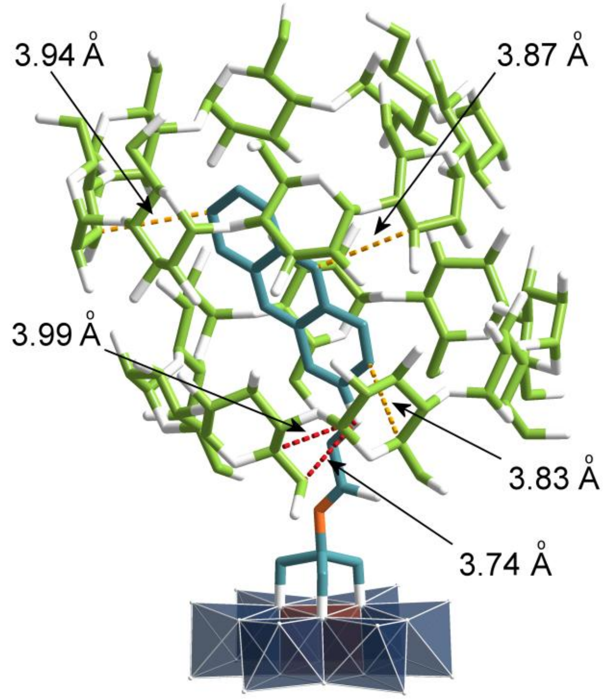

Figure S51. The crystal structure of **3s**@*γ*-CD showing the close spatial interactions between **3s** and *γ*-CD.

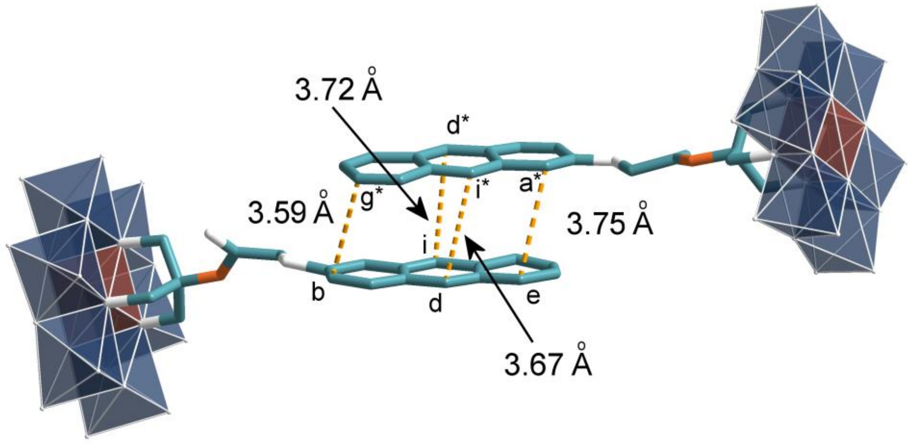

Figure S52. The close spatial distances between two **3s** molecules in **3s**@γ-CD.

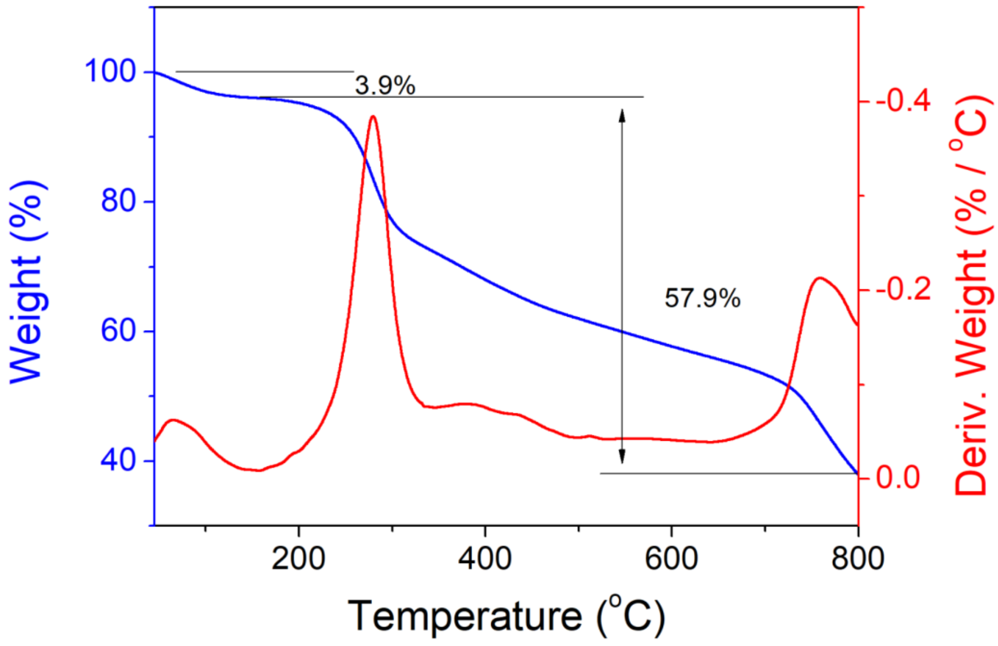

Figure S53. The TG-DTG curve of complex **3s**@γ-CD. The first stage weight loss of 3.9% is due to the loss of 6 crystalline H_2O. The rest weight loss of 57.9% (calcd 58.0%) is due to the loss of organic component.

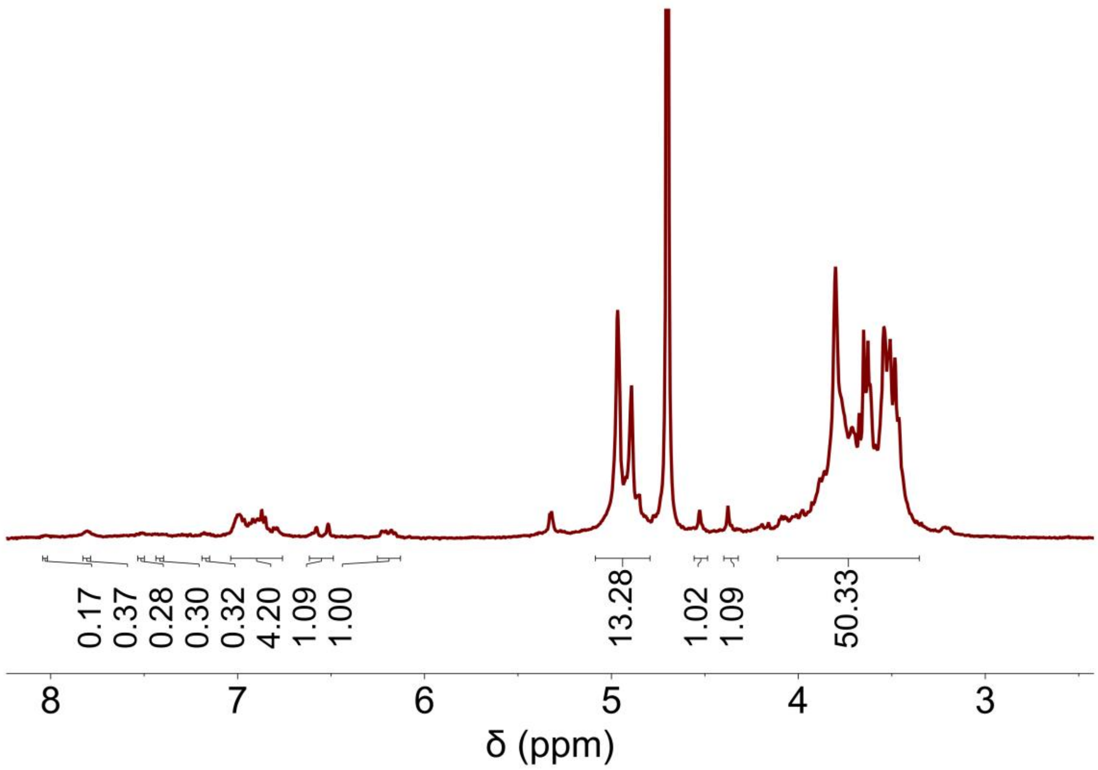

Figure S54. The ^{1}H-NMR spectrum of **4**@γ-CD in D_2O. Bulk samples of complex **3s**@γ-CD were used for UV irradiation for 16 hours to generate **4**@γ-CD. Integration of the NMR signals suggested there were nearly 12% of **3s**@γ-CD existed in **4**@γ-CD.

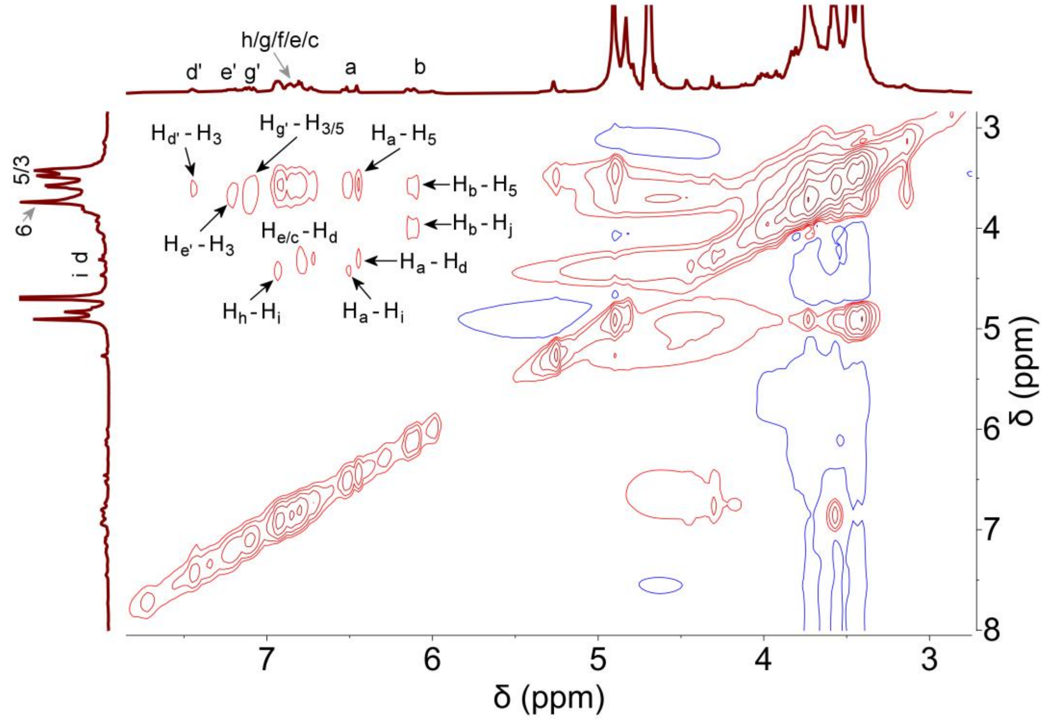

Figure S55. The 2D NOESY ^{1}H NMR spectrum of **4**@γ-CD in D_2O.

Section 4. The UV-Vis and fluorescence spectra of [3]rotaxane **4**@γ-CD

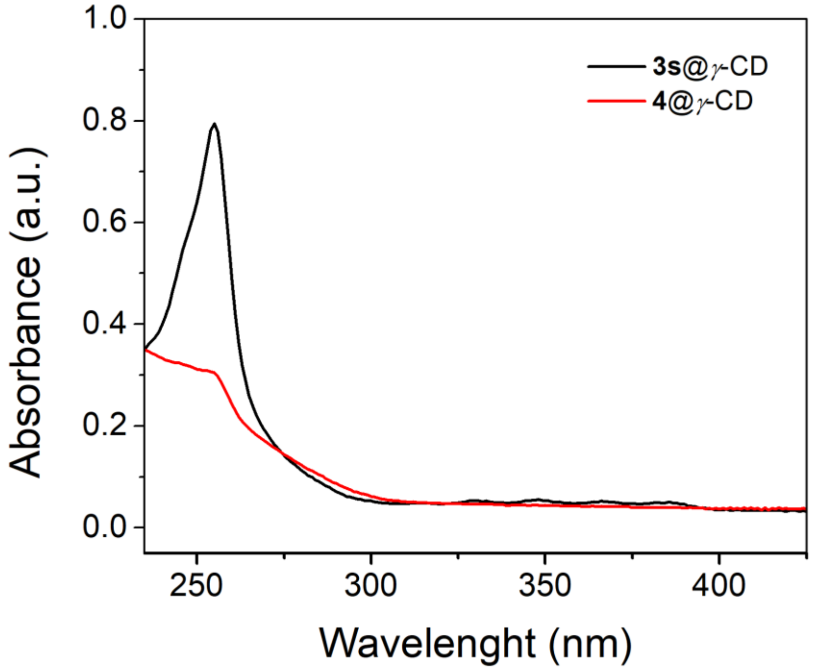

Figure S56. The UV-Vis spectra of **3s**@γ-CD and **4**@γ-CD.

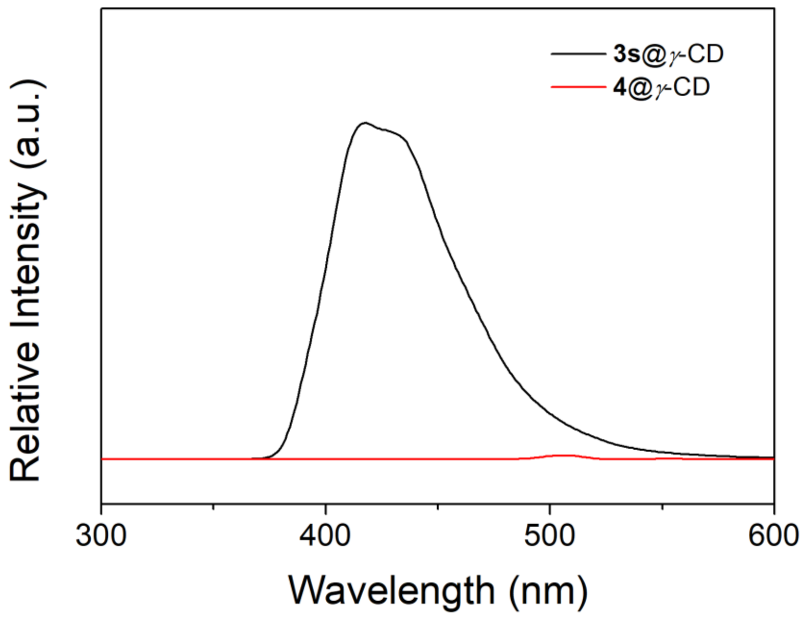

Figure S57. The fluorescence spectra of **3s**@γ-CD and **4**@γ-CD.

Section 5. Single-crystal images of all the hybrid (*pseudo*-)rotaxanes

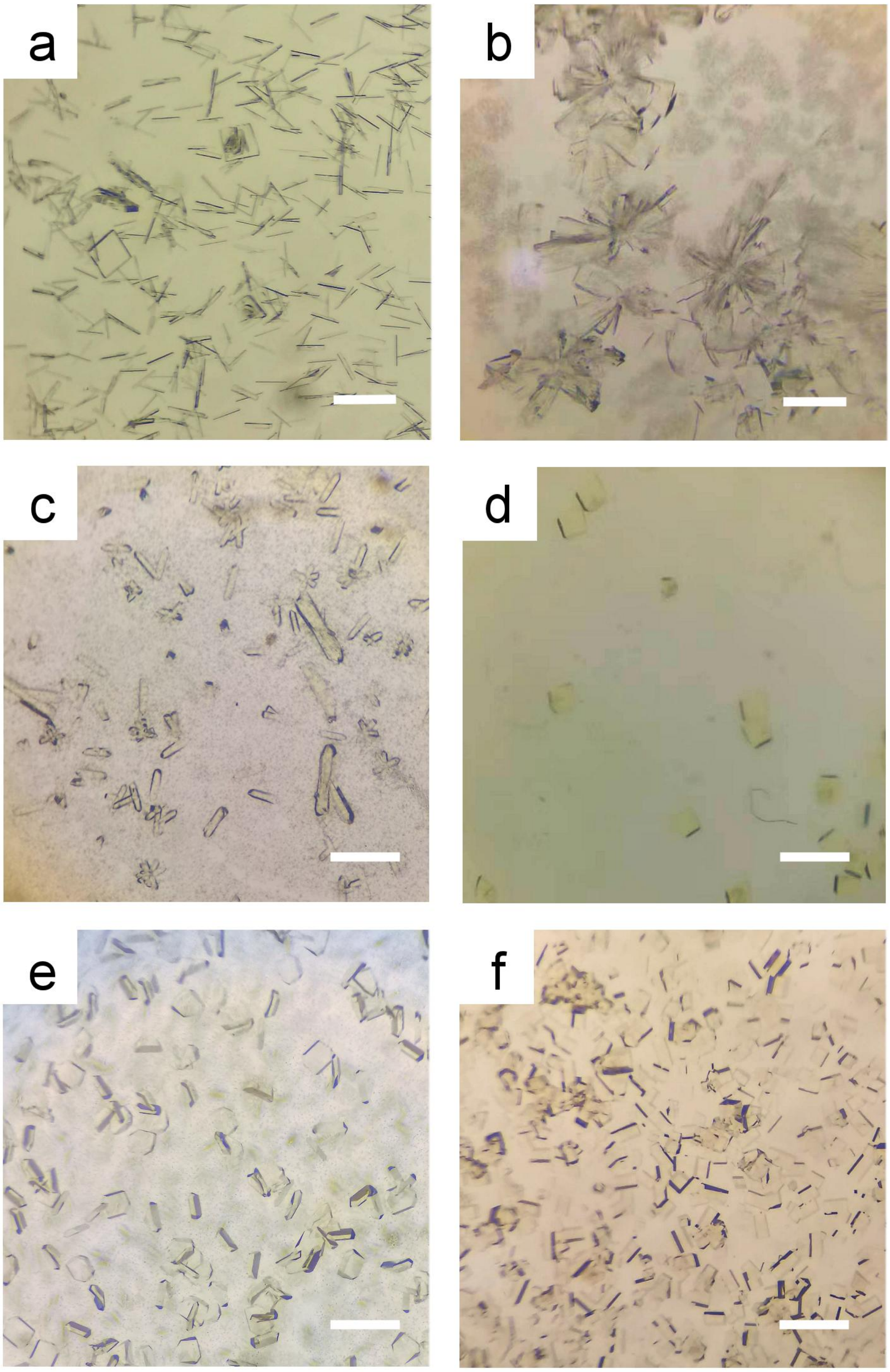

Figure S58. The single-crystal images of a) **1**@γ-CD, b) **1s**@γ-CD, c) **2**@γ-CD, d) **2s**@γ-CD, e) **3**@γ-CD, and f) **3s**@γ-CD. Scale bar: 100 μm.

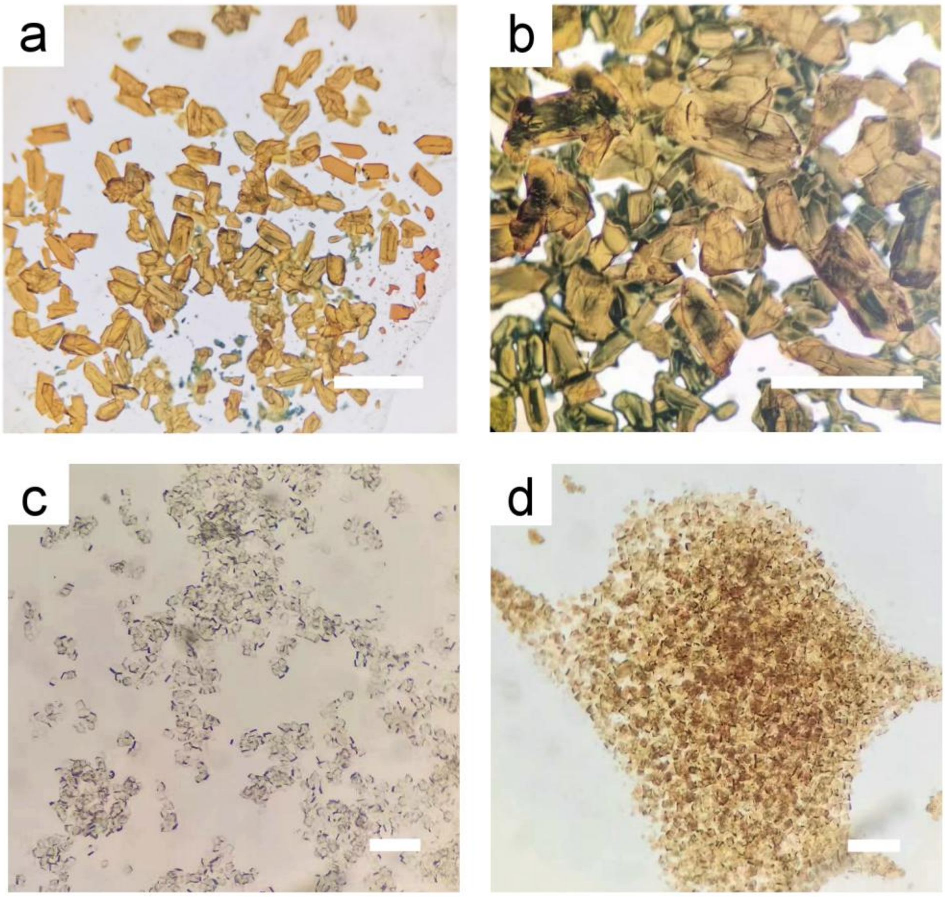

Figure S59. The crystal images of **3**@γ-CD in Paratone oil after irradiated at 365 nm for a) 8 hours, and b) 16 hours. The images of **3s**@γ-CD in Paratone oil c) before and d) after irradiated at 365 nm for 8 hours. Scale bar: 100 μm.

Section 6. The reversible transition of **4**@γ-CD to **3s**@γ-CD

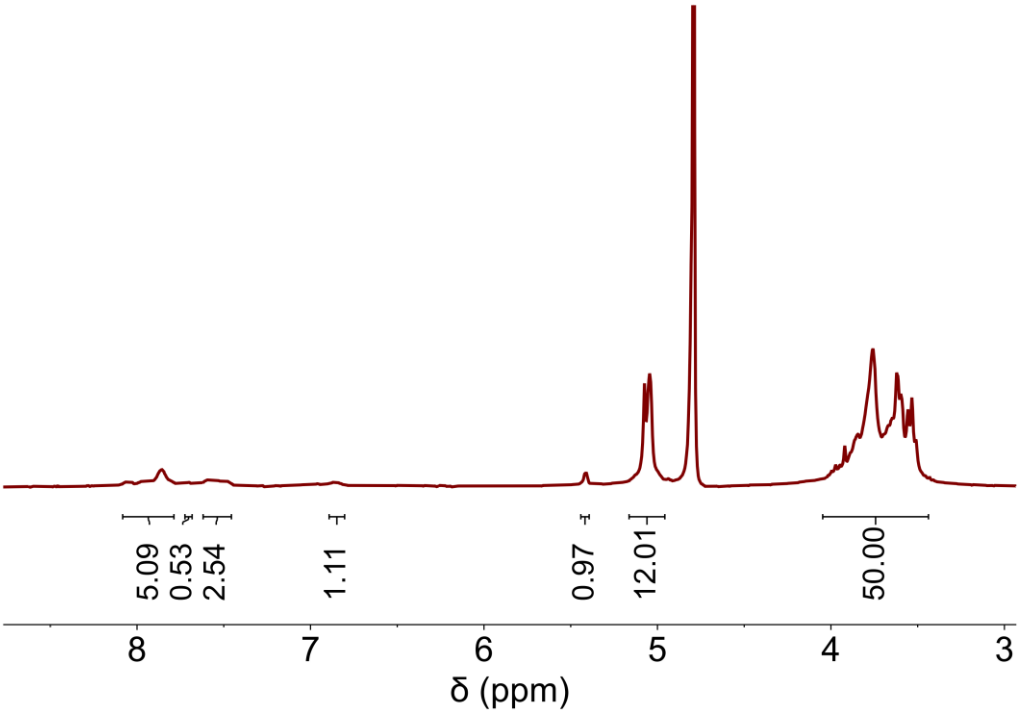

Figure S60. The ^{1}H-NMR spectrum of sample **4**@γ-CD in D_2O after thermal treatment at 150 ºC for 2 hours.

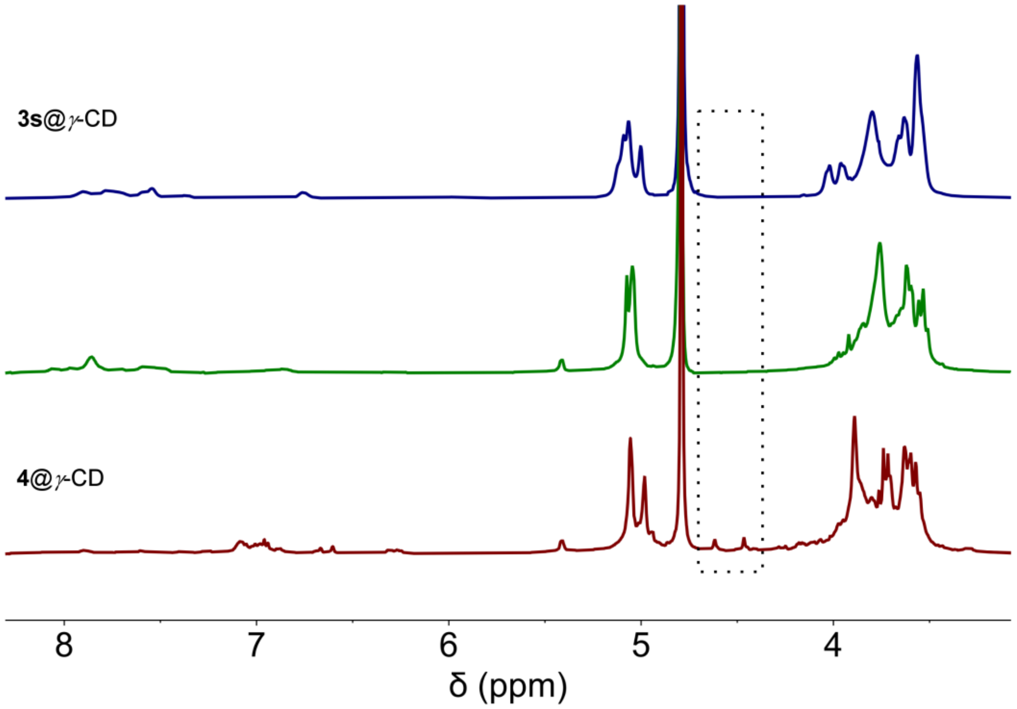

Figure S61. The ^{1}H-NMR spectra comparison of complex **3s**@γ-CD (top), **4**@γ-CD after thermal treatment (middle), and complex **4**@γ-CD (bottom) in D_2O.

Section 7. Crystallographic data

Table S3 Summary of all the single crystal data.

	**1**@*γ*-CD	**1s**@*γ*-CD	**2**@*γ*-CD	**3**@*γ*-CD	**3s**@*γ*-CD	**4**@*γ*-CD
Empirical formula	$C_{63}H_{175.13}AlK_3Mo_6NO_{122.23}$	$C_{232}H_{379.5}Al_5Mo_{30}N_5Na_{15}O_{362}$	$C_{74}H_{83.5}AlK_3Mo_6NO_{75}$	$C_{73}H_{81.5}AlK_{2.5}Mo_6NNa_{0.5}O_{71}$	$C_{68}H_{97}AlMo_6NNa_3O_{87.5}$	$C_{68}H_{73}AlMo_6NNa_3O_{76}$
Formula weight	3622.81	12388.83	2906.83	2820.75	3000.05	2791.86
Temperature/*K*	200.0	220.0	200.0	200.00	290.59	193.0
Crystal system	monoclinic	triclinic	orthorhombic	orthorhombic	orthorhombic	orthorhombic
Space group	*C*2	*P*1	$P2_12_12_1$	$P2_12_12_1$	$P2_12_12_1$	$P2_12_12_1$
a / Å	37.5560(14)	14.7761(2)	25.6327(6)	25.5816(7)	24.7051(5)	24.6852(8)
b / Å	46.9830(15)	27.0556(5)	30.4836(7)	30.3816(9)	27.7438(6)	27.9765(9)
c / Å	29.5236(10)	33.4587(5)	31.2430(7)	31.1857(8)	38.6548(10)	39.4067(13)
α / °	90	67.472(2)	90	90	90	90
β / °	128.027(2)	88.2170(10)	90	90	90	90
γ / °	90	83.9940(10)	90	90	90	90
V / Å^3	41036(3)	12286.9(4)	24412.6(10)	24237.8(12)	26494.5(10)	27214.5(15)
Z	12	1	8	8	8	8
ρ g/cm^3	1.759	1.674	1.582	1.546	1.504	1.363
GOOF on F^2	0.848	1.014	1.026	0.994	0.997	1.048
Final *R* indexes [I>=2σ (I)]	R_1 = 0.0701, wR_2 = 0.1706	R_1 = 0.0924, wR_2 = 0.2357	R_1 = 0.0466, wR_2 = 0.1108	R_1 = 0.0851, wR_2 = 0.2327	R_1 = 0.0890, wR_2 = 0.2186	R_1 = 0.0944, wR_2 = 0.2525
Final *R* indexes [all data]	R_1 = 0.1527, wR_2 = 0.2163	R_1 = 0.1374, wR_2 = 0.2849	R_1 = 0.0604, wR_2 = 0.1194	R_1 = 0.1286, wR_2 = 0.2649	R_1 = 0.1745, wR_2 = 0.2803	R_1 = 0.1269, wR_2 = 0.2869
Flack parameter	0.202(10)	0.21(3)	-0.009(8)	-0.683(13)	0.03(2)	0.21(5)
Extinction Coefficient	-	-	-	0.00107(11)	-	-
Largest diff. peak/hole e Å^{-3}	0.46/-0.53	2.75/-1.13	1.04/-0.87	1.78/-0.91	1.58/-0.58	1.65/-0.79

Section 8. References

1. Manikumari, S.; Shivaiah, V.; Das, S. K. Identification of a near-linear supramolecular water dimer, $(H_2O)_2$, in the channel of an inorganic framework material. *Inorg. Chem.* **2002**, *41*, 6953-6955.
2. Zhang, J.; Long, Y.; Xuan, W.; Lin, C.-G.; Song, Y.-F. Preparation of polyoxometalate-chalcone hybrid and its photo-polymerization property (in Chinese). *Chin. Sci. Bull.* **2017**, *62*, 685-692.
3. Lin, C.-G.; Hutin, M.; Busche, C.; Bell, N. L.; Long, D.-L.; Cronin, L. Elucidating the paramagnetic interactions of an inorganic-organic hybrid radical-functionalized Mn-Anderson cluster. *Dalton Trans.* **2021**, *50*, 2350-23553.
4. Zhang, M.-M.; Yin, Y.-A.; Chen, W.-J.; Lin, C.-G.; Wei, Y.; Song, Y.-F. Asymmetric modification of Anderson-type polyoxometalates towards organic-inorganic homo- and hetero-cluster oligomers. *Inorg. Chem. Front.* **2023**, *10*, 1712-1720.
5. Sheldrick, G. M. SHELXT-integrated space-group and crystal-structure determination. *Acta Cryst. A*, **2015**, *71*, 3-8.
6. Sheldrick, G. M. Crystal structure refinement with SHELXL. *Acta Cryst. C*, **2015**, *71*, 3-8.
7. Dolomanov, O. V.; Bourhis, L. J.; Gildea, R. J.; Howard, J. A. K.; Puschmann, H. OLEX2: a complete structure solution, refinement and analysis program. *J. Appl. Cryst.* **2009**, *42*, 339-341.
